# Identification of SARS-CoV-2 Mpro inhibitors through deep reinforcement learning for *de novo* drug design and computational chemistry approaches†

**DOI:** 10.1039/d4md00106k

**Published:** 2024-04-29

**Authors:** Julien Hazemann, Thierry Kimmerlin, Roland Lange, Aengus Mac Sweeney, Geoffroy Bourquin, Daniel Ritz, Paul Czodrowski

**Affiliations:** a Physical Chemistry, Chemistry Department, Johannes Gutenberg University Duesbergweg 10-14 55128 Mainz Germany czodpaul@uni-mainz.de; b Drug Discovery Chemistry, Idorsia Pharmaceuticals Ltd. Hegenheimermattweg 91 4123 Allschwil Switzerland

## Abstract

Severe acute respiratory syndrome coronavirus 2 (SARS-CoV-2) has caused a global pandemic of coronavirus disease (COVID-19) since its emergence in December 2019. As of January 2024, there has been over 774 million reported cases and 7 million deaths worldwide. While vaccination efforts have been successful in reducing the severity of the disease and decreasing the transmission rate, the development of effective therapeutics against SARS-CoV-2 remains a critical need. The main protease (Mpro) of SARS-CoV-2 is an essential enzyme required for viral replication and has been identified as a promising target for drug development. In this study, we report the identification of novel Mpro inhibitors, using a combination of deep reinforcement learning for *de novo* drug design with 3D pharmacophore/shape-based alignment and privileged fragment match count scoring components followed by hit expansions and molecular docking approaches. Our experimentally validated results show that 3 novel series exhibit potent inhibitory activity against SARS-CoV-2 Mpro, with IC_50_ values ranging from 1.3 μM to 2.3 μM and a high degree of selectivity. These findings represent promising starting points for the development of new antiviral therapies against COVID-19.

## Abstract 2

A pragmatic approach to the discovery of new SARS-COV-2 Mpro inhibitors by combining generative chemistry and computational chemistry approaches.

## Graphical abstract

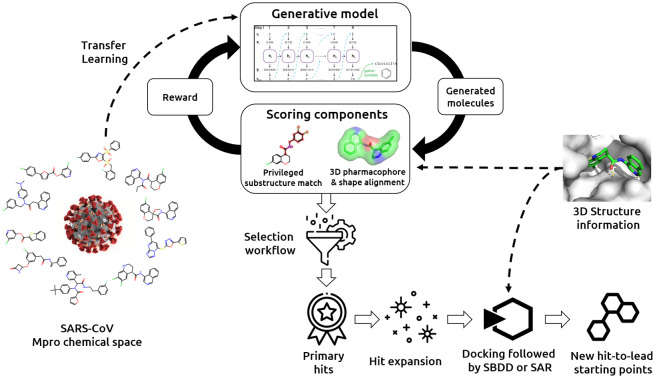

## Introduction

Identified in China in December 2019, the severe acute respiratory syndrome coronavirus 2 (SARS-CoV-2) quickly escalated into a global pandemic as the infectious agent of COVID-19, spreading rapidly across the world, resulting in more than 774 million reported cases and causing 7 million deaths.^1^ In parallel, vaccines and monoclonal antibody treatments became accessible within a year to combat the severe coronavirus disease 2019 (COVID-19). However, the urgent need persisted for therapeutic solutions for individuals who were unvaccinated, immunocompromised, or experiencing diminished vaccine immunity.^2^ One promising approach is the development of small molecule inhibitors targeting the main protease of the virus, also known as Mpro. This enzyme plays a critical role in the replication of the virus, making it an attractive target for drug discovery.^3,4^ Nirmatrelvir–ritonavir (Paxlovid) and ensitrelvir are Mpro inhibitors which were approved for the treatment of COVID-19 in 2021 and 2022, respectively. Both drugs showed no inhibitory activity against host–cell proteases, such as cathepsin L, suggesting their high selectivity for coronavirus proteases.^5–7^ Moreover, the growing emergence of drug resistance underscores the necessity for the continued development of Mpro inhibitors, despite the already significant progress.^8,9^

In this context, the Covid Moonshot effort is a collaborative and open-science initiative^10^ aimed at discovering drugs to target the SARS-CoV-2 main protease. The campaign utilized crowdsourcing, high-throughput structural biology, machine learning (ML), and molecular simulations to identify new chemical series with potent nanomolar activity. Through this effort, a comprehensive understanding of the structural plasticity of the main protease was obtained, along with extensive structure–activity relationships for multiple chemotypes and a large dataset of biochemical activities. Notably, the initiative achieved a significant milestone by openly sharing all compound designs, crystallographic data, assay data, and synthesized molecules, creating a large knowledgebase for future anti-coronavirus drug discovery that is accessible and free from intellectual property restrictions. The Covid Moonshot efforts have been highly beneficial to our research. They discovered several potent chemical series and provided valuable insights into the structure of the SARS-CoV-2 main protease. The open sharing of data has created a valuable resource for our anti-coronavirus drug discovery and helped accelerating our progress.

In addition, in recent years, ML methods have gained popularity in the field of drug discovery due to their ability to accelerate the identification of novel drug candidates.^11,12^ One such approach is deep reinforcement learning for *de novo* drug design, which involves a trained neural network, scoring components and a reward function. The trained neural network or deep generative model (DGM) generates novel chemical structures, the generated molecules are ranked by scoring components and a reward function combines all the scores. The generative model iteratively adapts its policy to maximize the reward and therefore generates, over time, molecules with desirable properties, such as high affinity, good pharmacokinetics, and low toxicity.

Researchers have applied a similar approach to the identification of epidermal growth factor (EGFR) inhibitors, using a generative model pre-trained with ChEMBL^13^ molecules and an EGFR predictive ML model. The generated structures were experimentally validated and led to the identification of several new bioactive molecules.^14^ Another similar approach employing reinforcement learning has been applied retrospectively to demonstrate that a DGM could generate active molecules where these molecules were not included in the training dataset.^15^ Finally, *de novo* design approaches, without reinforcement learning, were applied to discover new SARS-COV-2 Mpro inhibitors for which two approaches were experimentally validated^16,17^ while two others were not.^18,19^

In this study, we focused on the design of non-covalent inhibitors and have used, to this end, REINVENT 2.0,^20^ an AI tool for *de novo* drug design developed by Thomas Blaschke *et al.* The generative engine of the tool is directed by a reinforcement learning (RL) module. We customized REINVENT by two additional components which we developed ourselves: a 3D pharmacophore/shape-alignment scoring component and a substructure match count scoring component. After the generation process, we employed several filtering steps to prune the number of hits. These primary hits underwent a hit expansion followed 3D structure-based selection through molecular docking.

As a result of these efforts, four new Mpro inhibitors were identified. One of them underwent optimization using a 3D-structure based approach, while another one was optimized using a classical structure activity relation (SAR) approach. The affinities of the compounds were measured by a Mpro FRET IC_50_ assay, with values ranging from 1.3 μM to 2.3 μM. However, one of the four compounds exhibited close similarity to known Mpro inhibitors and was therefore excluded from further consideration.

Based on these findings, the three remaining compounds were selected as starting points for additional refinement in the Idorsia hit-to-lead pipeline.

While our approach could probably be used for the discovery of covalent inhibitors, we decided to solely concentrate our effort in the identification of non-covalent inhibitors.

## Results and discussion

**Fig. 1:**
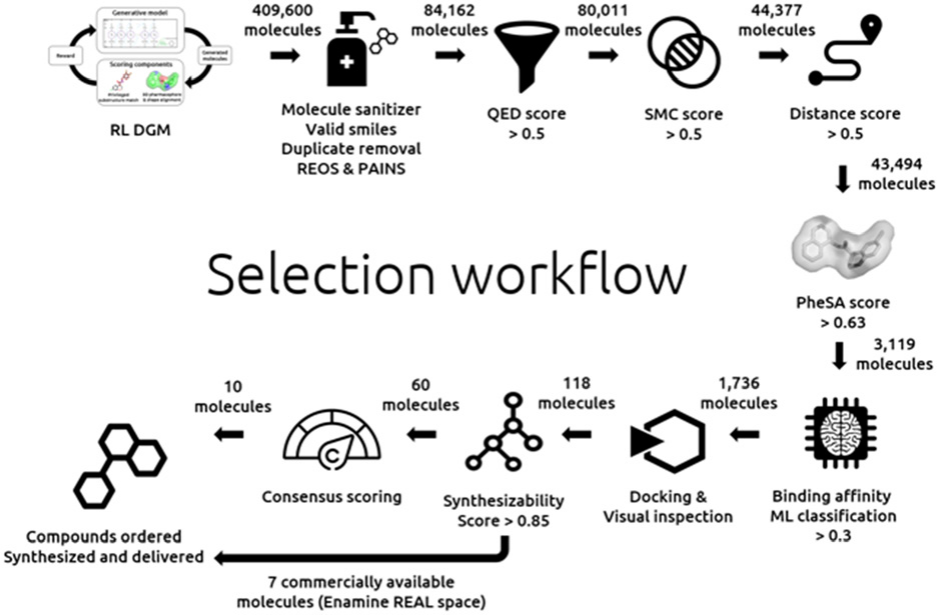
Design and selection workflow.

The details of our selection workflow can be found in the “Experimental section”. However, the different steps can be identified in Fig. 1 to which we will frequently refer in the remaining manuscript. A hit expansion and structure-based approaches follow this selection workflow.

### Scoring components for the reinvent RL

A 3D pharmacophore and shape alignment scoring component from single active conformer queries was adapted as a REINVENT scoring component. This component was derived from PheSA^21^ (pharmacophore-enhanced shape alignment), a tool developed at Idorsia. The reference Mpro inhibitor dataset was used to query the PDB files (obtained from the https://fragalysis.diamond.ac.uk/ website) and to extract the active conformers of the exact same ligand of the 225 reference inhibitors. 69 active ligands were identified and extracted from PDB files. PheSA descriptors were generated for these query active conformers. The 69 inhibitors were clustered, and non-covalent representatives of each cluster were selected. In total, 23 query compounds were selected. The 23 PheSA queries (single active conformers) were used to screen the test set made of 69 active and 6000 compounds considered inactive as randomly picked from the enamine HTS collection. Molecules with a PheSA score > = 0.63 were considered predicted “active”.

PheSA validation results:

• Precision: 0.168.

• Sensitivity: 0.532.

• Kappa: 0.238.

• ROC AUC: 0.88.

The SMC component is a privileged fragment substructure match scoring component. A matched molecular pair analysis was performed on the reference Mpro inhibitor dataset. Fragments that allowed a 2-fold gain in potency were selected. Fragments containing covalent warheads were discarded. The selected fragments were then combined with the noncovalent fragment hits discovered in the frame of the Covid Moonshot crystallographic screen.^22^ Only fragments with Mw < = 350 g mol^−1^ were retained and duplicates were removed. In total a collection of 265 privileged fragments were obtained.

The following analysis was performed with the Knime analytical platform^23^ combined with Python:

The COVID Moonshot FRET activity dataset was split into two groups:

• 354 active molecules < = 10 μM (FRET IC_50_).

• 586 inactive molecules > 10 μM (FRET IC_50_).

A substructure match was performed using these compounds against a set of selected privileged fragments.

The Python script counts how many fragments would match a COVID Moonshot compound.

Again, the reference compounds were split into two groups.

• 815 matching counts < = 15 fragments.

• 125 matching counts > 15 fragments.

The results of this validation are depicted in Fig. S1 of the ESI† and demonstrate the beneficial impact on inhibitory activity for molecules displaying a high SMC as we can observe an enrichment of “active” molecules for compounds with a matching count >15 fragments. All datasets are described in the “Experimental section” and are electronically available as part of the ESI.†

The Python script was written to allow generated molecules to be evaluated against a list of privileged fragment substructures. The script counts how many privileged fragments match a molecule. Then a membership trapezoidal scoring function is applied as depicted in Fig. S2 of the ESI.†

The script can score molecules based on their substructure match count against privileged fragments. The script was implemented as a scoring component in the DGM RL allowing the generation of molecules containing many privileged fragments.

### AI generated molecules

The quantitative estimate of the drug-likeness QED^24^ scoring component was used to help the DGM in generating “drug-like” molecules. The Jaccard distance component was employed to assist the model in generating molecules dissimilar to already known Mpro inhibitors. Two additional self-developed scoring components, the 3D pharmacophore/shape alignment (PheSA) component and the SMC scoring component, were applied to help the DGM to generate Mpro bioactive molecules.

Two DGM scenarios were considered for generating molecules, for the first scenario, “exploration”, the pre-trained DGM was used as such to explore the chemical space for the generation of new Mpro inhibitors. For the second scenario, “exploitation”, the DGM was retrained with a set of known Mpro inhibitors collected from the Open Science consortium COVID Moonshot and the ChEMBL database to exploit the known Mpro chemical space.

The two scenarios were then used in an RL fashion employing the scoring components described above. Both systems were trained for 500 epochs for the exploitation mode and 1000 epochs for the exploration mode until the scores of each individual scores plateaued out.

During the exploitation approach, the originally trained prior with ChEMBL molecules was retrained for 40 epochs with the DGM-TL dataset containing 338 known Mpro inhibitors. The purpose of this “Transfer Learning” approach is to bias the original DGM with compounds from the Mpro chemical space with the expectation that the generated molecules will be similar to the ones it was trained for.

Once the prior was retrained, the system was used in a RL context for 500 epochs where the PheSA, Jaccard distance, SMC, and QED scoring components were utilized to score the generated molecules and assist the system in understanding the desired properties. The best RL agent was obtained with the following parameters:

• Epochs: 500.

• Scoring components:

∘ QED weight: 1.0.

∘ PheSA weight: 4.0.

∘ SMC weight: 1.0.

∘ Jaccard distance weight: 1.0.

The individual scores of the exploitation mode were monitored with Tensorboard as depicted in Fig. S7 of the ESI.† The graphs show a score increase across all scoring components. However, a plateau is reached for PheSA after 350 epochs and for QED, while unstable, after 150 epochs.

During the exploration approach, we utilized the original pre-trained prior based on ChEMBL molecules without any retraining. This model was directly applied in a reinforcement learning setting, incorporating scoring components like PheSA, Jaccard distance, SMC, and QED. We ran this setup for 1000 epochs to help the system grasp the targeted molecular properties. The aim of this second approach was to ascertain if the system could produce the desired molecules with reduced bias. The best RL agent was obtained with the following parameters:

• Epochs: 1000.

• Scoring components:

○ QED weight: 1.0.

○ PheSA weight: 20.0.

○ SMC weight: 2.0.

○ Jaccard distance weight: 1.0.

The individual scores of the exploration mode were monitored with Tensorboard as depicted in Fig. S8 of the ESI.† The plots show an increase in learning for PheSA and SMC scores. A plateau is reached after 600 epochs for PheSA and 400 epochs for SMC. For the Jaccard distance, a steep increase was observed then a decline and again an increase. For QED, the score improved until 400 epochs and then deteriorated.

By comparing the 2 approaches, focused and exploration, it is noticeable that the focused approach allowed an overall better and quicker learning process. It is also interesting to see that for PheSA and Jaccard distance a similar score can be obtained for both approaches. For QED, it was difficult to improve or even maintain the original scores while using the exploration approach. Finally, the transfer learning significantly helped the system to generate structure containing privileged fragments as we can see by comparing both SMC score figures.

The above trained agents were used to generate about 400 000 molecules. This design step is depicted in the top left of Fig. 1.

### AI generated molecule selection workflow

A machine learning model for bioactivity predictions was developed and validated with the Knime analytical platform for the selection of the AI generated molecules. The ML dataset was labelled as follows:

• Label 1 (active) < = 10 000 nM.

• Label 0 (inactive) > 10 000 nM.

Validation method: a random molecule (arbitrarily assigned as compound 1) was picked, and its closest analogue (highest Tanimoto/Morgan FP) is identified. The identified closest analogue is assigned to compound 2 and, again, its closest analogue (excluding its predecessor) is identified as the following compound. The same logic is applied until all molecules got a compound number. Since there is no time stamp on this dataset, a virtual chronology was simulated by sorting the compounds by similarity. Molecules were sorted from 1 to 739 and a 10-fold linear sampling was applied. Random Forest and XGBoost were evaluated with either all possible RDKit^25^ fingerprints (FPs) or calculated physico-chemical properties. DeepChem^26^ graph convolutional neural network (GCNN) was also evaluated with 25, 50, 75 and 100 epochs.

List of the best performers:

• XGBoost-Global_chiral_ECFP6.

• GCNN-global_70_epochs.

• XGBoost-Global_ECFP6.

• RF-Global_Torsion.

Then the following split was applied 80/10/10 for training/validation/testing of a model ensemble (see the list above).

ML performance on the validation set:

• ROC AUC: 0.881.

• Kappa: 0.517.

• Class probability threshold: 0.215 (adjusted threshold).

ML performance with the test set:

• ROC AUC: 0.506.

• Kappa: −0.027.

• Class probability threshold: 0.5.

The above results suggest that a ML (RF-FPs, XGBoost-FPs and GCNN) approach can predict compounds being similar to compounds in the training set (ESI,† Fig. S3) but is not able to make useful prediction for dissimilar compounds (ESI,† Fig. S4). In these conditions, a ML classification applied to RL or even post-processed generated molecules would be suboptimal.

In order to identify better ML models, the extended 3-dimensional fingerprint^27^ (E3FP default parameters) was evaluated on the previously assembled ML dataset. A similarity sorting split 80/10/10 was performed with an ensemble of models (XGBoost and RF).

ML performance on the validation set:

• ROC AUC: 0.898.

• Kappa: 0.761.

• Class probability threshold: 0.5.

ML performance with the test set:

• ROC AUC: 0.642.

• Kappa: 0.231.

• Class probability threshold: 0.359 (adjusted threshold).

The model ensemble XGBoost-E3FP and RF-E3FP shows better predictive performance (ESI,† Fig. S5 and S6) compared to the previous ML model ensemble (RF-FPs, XGBoost-FPs and GCNN). While the performance, still weak, would not speak in favor of using this model in RL, it has been considered that a post-filtering of DGM generated molecules could be useful with a class probability threshold >0.359 as it would help to exclude compounds with a higher probability of being inactive.

The generated molecules were filtered with the following filter cascade:

• Initial sampling: 409 600 molecules.

• Molecule sanitizer (valid smiles, duplicate filter, and SMARTS filters): 84 162 molecules.

• QED >0.5: 80 011 molecules.

• SMC >0.5: 44 377 molecules.

• Jaccard distance >0.5: 43 494 molecules.

• PheSA score >0.63: 3119 molecules.

• Bioactivity ML model predictions >0.359: 1736 molecules.

The SMARTS filters were employed to remove molecules containing reactive functions or to remove molecules that are in general not desired by medicinal chemists. These SMARTS strings were assembled in a previous work.^28^

**Fig. 2:**
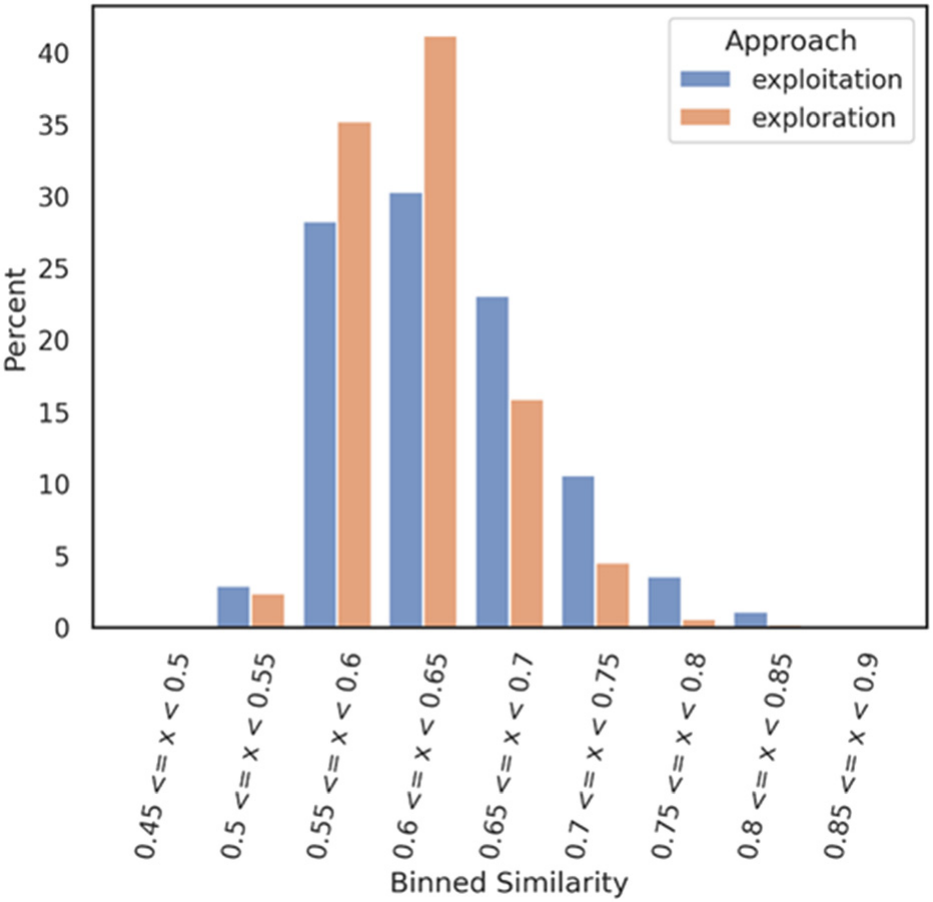
Nearest neighbour similarity of the exploration and exploitation datasets against the DGM-TL dataset. Each subset (exploration and exploitation) was normalized independently.

An analysis was realized right after the bioactivity ML model prediction filter where two smaller datasets of 1007 molecules for the exploration mode and 729 for the exploitation mode were obtained. The two datasets (1736 molecules) were compared to each other as follows: a Tanimoto nearest neighbor analysis was performed on both datasets; exploitation and exploration. Each molecule of each dataset was compared with each molecule of a Mpro reference compound dataset (DGM-TL). Each comparison is based on the Tanimoto similarity coefficient (1-Jaccard distance) and allows the identification of the most similar molecule in the DGM-TL. The similarity values were then binned with a range of 0.05 and a distribution bar chart was plotted (Fig. 2) with the Python library seaborn.^29^ This analysis gives a solid understanding of how similar or dissimilar a dataset compared to a reference dataset looks like.

Fig. 2 shows that the exploration dataset exhibits a greater abundance of dissimilar compounds when compared to the DGM-TL compounds, highlighting the capacity of the exploration mode to generate more novel compounds. This discrepancy can be attributed to the absence of transfer learning in the exploration approach, which encourages greater diversity in the generated molecules. Conversely, the exploitation mode involves retraining the generative model on the Mpro chemical space, resulting in a reduced potential for novelty. In this mode, the generative model predominantly produces molecules that align with its retraining, limiting the exploration of new chemical space.

Then, a Tanimoto self-nearest neighbor analysis was performed on both datasets, exploitation and exploration respectively. Each molecule of each dataset was compared against each other molecules of the same dataset to evaluate the self-diversity of the dataset. Each comparison is based on the Tanimoto similarity coefficient (1-Jaccard distance) and allows identification of the most similar molecule within the same training set. The similarity values were then binned with a range of 0.05 and a distribution bar chart was plotted with the Python library seaborn. This analysis allows assessment of the self-diversity of a dataset.

**Fig. 3:**
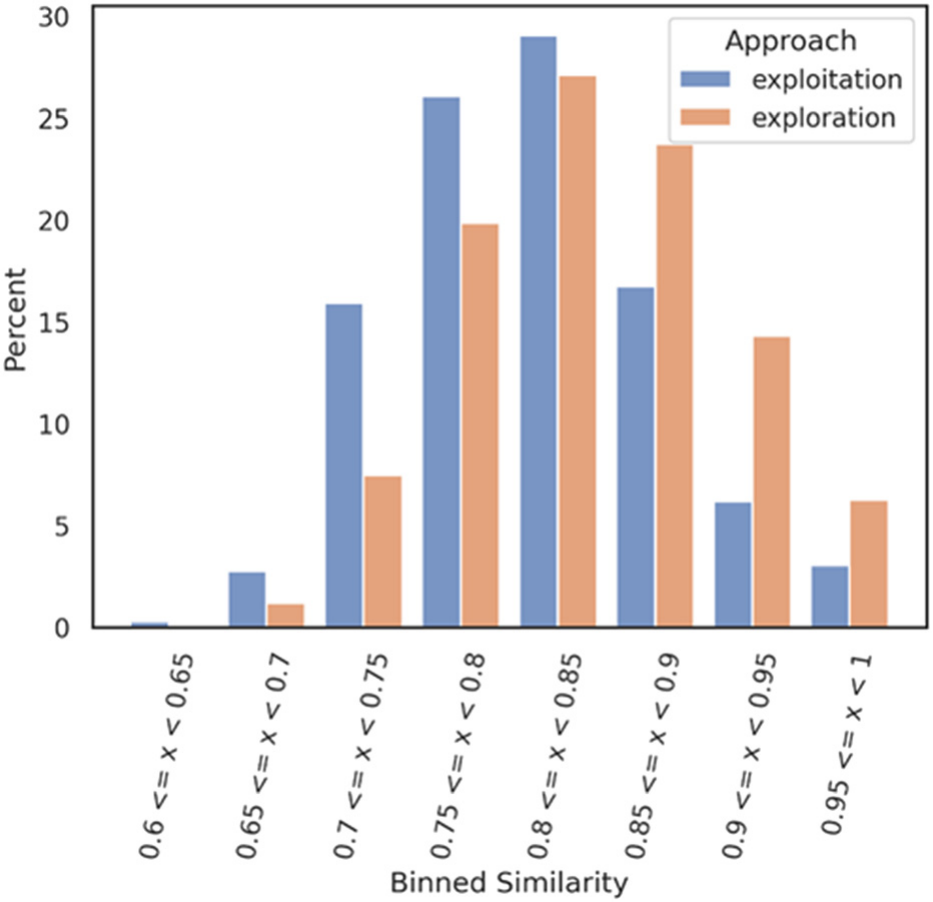
In orange, self-nearest neighbor similarity of the exploration dataset against itself. In blue, self-nearest neighbor similarity of the exploitation dataset against itself. Each subset (exploration and exploitation) was normalized independently.

Fig. 3 illustrates a distribution shift between the two datasets, revealing an interesting finding. The exploitation datasets demonstrate a reasonable level of self-diversity, while surprisingly, the exploration mode appears to have achieved a lower level of self-diversity. One potential explanation for this unexpected result is that the exploration generative model tends to generate compounds that are dissimilar to the Mpro DGM-TL dataset but exhibit self-similarity. However, further investigation is required to confirm this hypothesis and gain a better understanding of the underlying factors at play.

According to the selection process of Fig. 1, all steps until the bottom right have been described and discussed.

### Docking with SeeSAR and GOLD

Three crystal structures corresponding to three non-covalent chemical series were selected for the molecular docking (SeeSAR and GOLD):

• MPro-×11294 (quinolone).

• MPro-×12692 (3-amino-pyridine).

• MPro-P0009 (Ugi-non-covalent).

These three crystal structures were selected for the diversity of their ligands covering the main non-covalent series identified by the Covid Moonshot and also to help overcome the high plasticity of Mpro.^30^

The docking validation dataset was docked into MPro-×12692, MPro-P0009 and MPro-×11294. As observed from the crystal structure, pharmacophore constraints (acceptor spheres) were added to guide the docking for key H-bond interactions with the conserved H163 and E166 residues (MPro-×12692 and MPro-P0009) or with the H163 and either N142 and/or G143 residues (MPro-×11294).

After docking, a visual inspection allowed us to filter out poses for which one or two of the key interactions were missing.

The compounds with acceptable poses were predicted to be active, and the other compounds (either non-acceptable binding poses or when SeeSAR could not generate poses) were predicted as negative.

The evaluation of the predictive power led to the following results:

• Precision: 0.64 (for 22 predicted active, 14 were actually active).

• Kappa: 0.24.

• ROC AUC: 0.62.

As we can see from the results, a docking approach does not represent an ideal classifier nevertheless, it allows, among other filters (PheSA, ML, SMC), enrichment of the final dataset with possible new inhibitors.

Filtering: the combined 1736 compounds were docked with SeeSAR following the above description (validation). Approximately 188 compounds were selected after visual inspection and according to their binding poses. Then, a hard cut-off was applied on the SeeSAR estimated affinity and compounds below 3500 μM were retained. Finally, 118 were selected after docking.

These 118 molecules were docked again into Mpro with the software Gold,^31^ this time not to exclude any further molecules but in order to get an additional score which was used in a final consensus scoring. Aizynthfinder^32^ was then employed to assess the synthesizability of these same 118 compounds. 60 compounds were considered synthesizable. Out of these 60 molecules, 7 compounds found to be commercially available (in the Enamine REAL space^33^) were directly selected and ordered. Finally, a consensus scoring, budget considerations and synthesizability constraints led to a selection of 10 additional compounds. In total 17 compounds were ordered and 16 could be synthesized, delivered, and biologically assessed.

Below is the selection workflow from the docking to the final selection as depicted in Fig. 1:

• Docking: 118 molecules.

• Synthesizability assessment: 60 molecules.

• 17 compounds selected: 7 commercially available (Enamine REAL space) compounds and 10 additional compounds with a high consensus score.

### Biological results of the AI generated molecules

**Fig. 4:**
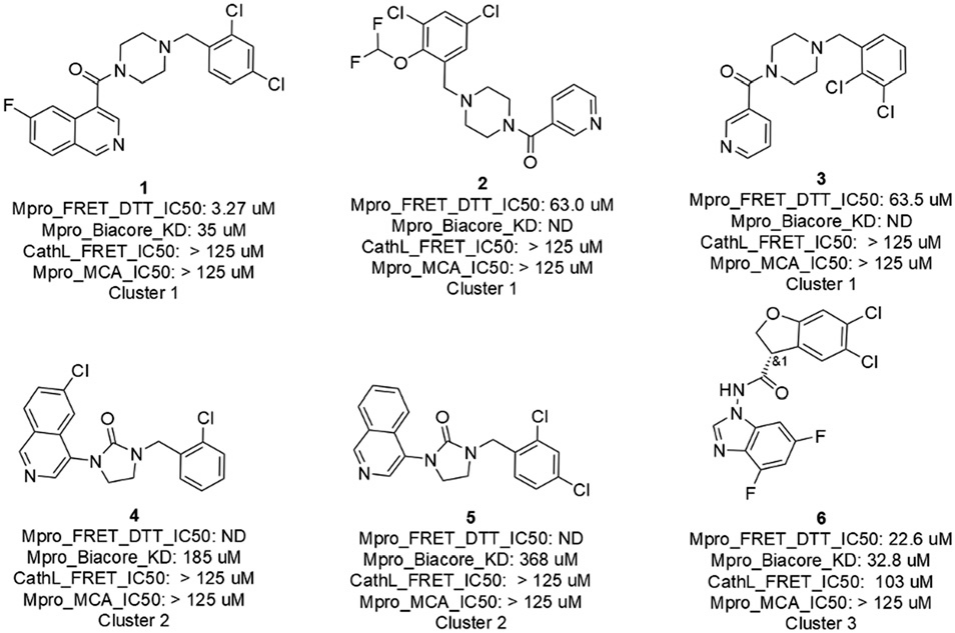
Primary hits with biological results of molecules originated from AI generative models, prioritized with a selection workflow, synthesized and delivered.

The 16 AI generated, synthesized, and delivered molecules were measured (ESI,† Table S1) in Mpro FRET (1 μM DTT), cathepsin L FRET, MCA quenching and for some cases in SPR (Biacore) assays. As depicted in Fig. 4 and 6 hits belonging to 3 clusters were identified. Cluster 1 (piperazine) showed activities between 3.3 and 63.5 μM. For cluster 2 (cyclized urea) the Mpro FRET IC_50_ could not be determined but a *K*_d_ of 185 and 368 μM could be measured by SPR demonstrating a weak binding affinity. Finally, the single compound in cluster 3 (*N*-benzoimidazol-1-yl-acetamide) showed an IC_50_ of 22.6 μM. Compound 21, depicted in the ESI† Table S1 and displaying a Mpro FRET IC_50_ of 66.9 μM, was not further considered due to a late delivery from our provider. All compounds (the 6 hits described above, compound 21 and the inactive compounds) and their biological data are shared in Table S1 of the ESI.†

While the 6 hits showed good to low affinity, they all, except for compounds 4 and 5 (not conclusive at this point), demonstrated a good selectivity profile as indicated by the cathepsin L IC_50_ values. All hits turned up not being auto-fluorescent as demonstrated by the MCA quenching assay results.

### Hit expansion and docking with glide

As mentioned above, these 6 hits were categorized in 3 clusters which we defined as 3 hit series: a piperazine, a cyclized urea and an *N*-benzoimidazol-1-yl-acetamide series. The 2D similarity was evaluated between the 6 hits and known Mpro inhibitors from ChEMBL and Covid Moonshot. Overall, a limited novelty was observed as compared to known Mpro inhibitors, therefore, to bring additional novelty, a hit expansion was performed for the 3 hit series.

Analogues, based on 2D similarity, were identified in either the Chemspace^34^ stock screening compounds or in the Idorsia corporate collection. The analogues were docked with H-bond constraints using grids generated from publicly available crystal structures.

The hit expansion (2D similarity analogue search) was performed for the 3 hit series (piperazine, cyclized urea and *N*-benzoimidazol-1-yl-acetamide). The hit expansion was done by searching the Idorsia corporate collection for the piperazine (cluster 1) and the *N*-benzoimidazol-1-yl-acetamide compound (cluster 3). For the cyclized ureas (cluster 2) a hit expansion was performed in the Chemspace in stock screening compounds as no analogues could be identified from the corporate collection.

• 1400 analogues of cluster 1 (positive control included) were identified.

• 4402 analogues of cluster 2 were identified.

• 128 analogues of cluster 3 were identified.

Positive control: 3 internal reference compounds + 2 Moonshot active compounds synthesized internally.

For cluster 1, the 1400 analogues were docked with Schrodinger Glide.^35^

The fragalysis complex Mpro-×11812 (co-crystalized ligand) was selected for its similarity with the cluster 1 hits and used to generate a docking grid.

The analogues were docked with grid-based constraints (at least two constraints):

• H-bond with H163.

• H-bond with G143.

• H-bond with C145.

• The docking poses were inspected, the best poses were starred, and 115 compounds were selected.

The positive control molecules were present in the top docking score list and provided some *in silico* evidence on the validity of the approach.

110 compounds were selected from the Idorsia corporate collection and submitted to the biological measurements.

For cluster 2, 4402 Chemspace analogues were docked with Schrodinger Glide.

The fragalysis complex Mpro-×11513 (co-crystalized ligand) was used for its similarity with the cluster 2 hits.

The docking (SP followed by XP) was performed on the analogues with grid-based constraints:

• H-bond with H163.

• H-bond with E166.

The docking poses were inspected, and the best poses were starred, and finally 43 compounds were selected, and a quote was requested at Chemspace.

38 compounds were delivered and submitted to biological measurements.

For cluster 3, 128 compounds were docked with Schrodinger Glide.

The fragalysis complex Mpro-×11612 (co-crystalized ligand) was used for its relative similarity with the cluster 3 hit.

The analogues were docked (SP followed by XP) with grid-based constraints:

• H-bond with H163.

• H-bond with E166.

The docking poses were inspected, and the best poses were starred, and finally 25 compounds were selected, and ordered from the Idorsia store for a biological assessment.

After visual inspection of the docking poses, the best compounds were selected, ordered, and measured in a FRET assay.

### Biological results of the compound selected after the hit expansion and glide docking

**Fig. 5:**
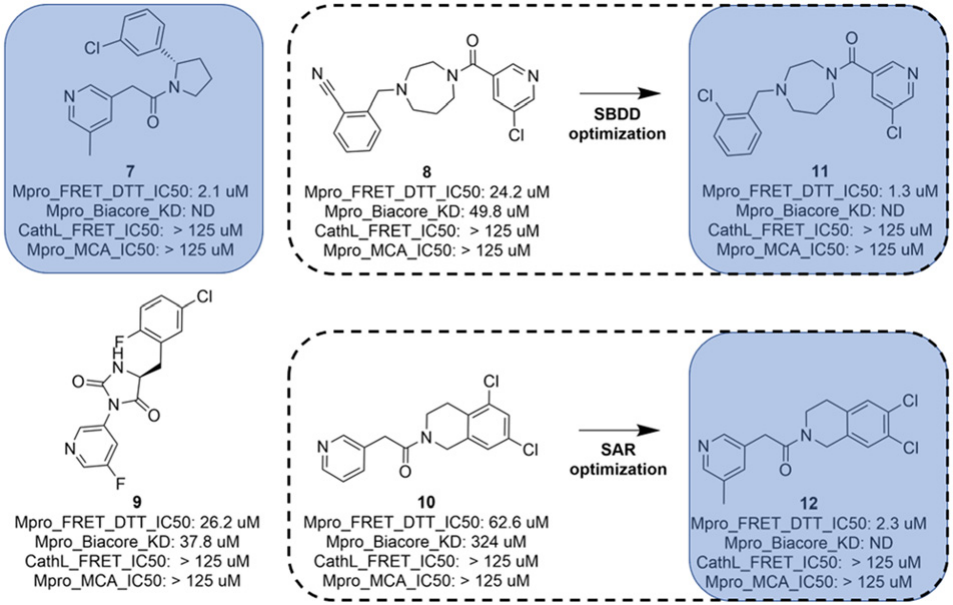
Compounds 7, 8, 9 and 10 with biological results were identified through hit expansion of the primary hits and molecular docking. Compounds 11 and 12 underwent a first round of SAR or SBDD of optimization.

In total, 173 compounds were considered for a biological assessment and then measured (ESI,† Table S2) in Mpro FRET (1 μM DTT), cathepsin L FRET, Mpro MCA quenching and for some cases in SPR (Biacore) assays. This hit expansion led to the identification of 4 interesting and diverse inhibitors (compounds 7, 8, 9 and 10) as depicted in Fig. 5. Compounds 8 and 10 originated from the hit expansion of cluster 1, compound 7 originated from the hit expansion of cluster 2 and compound 9 originated from the hit expansion of cluster 3. Additional Mpro inhibitors were identified with the approach but cannot be disclosed due to corporate restrictions.

**Fig. 6:**
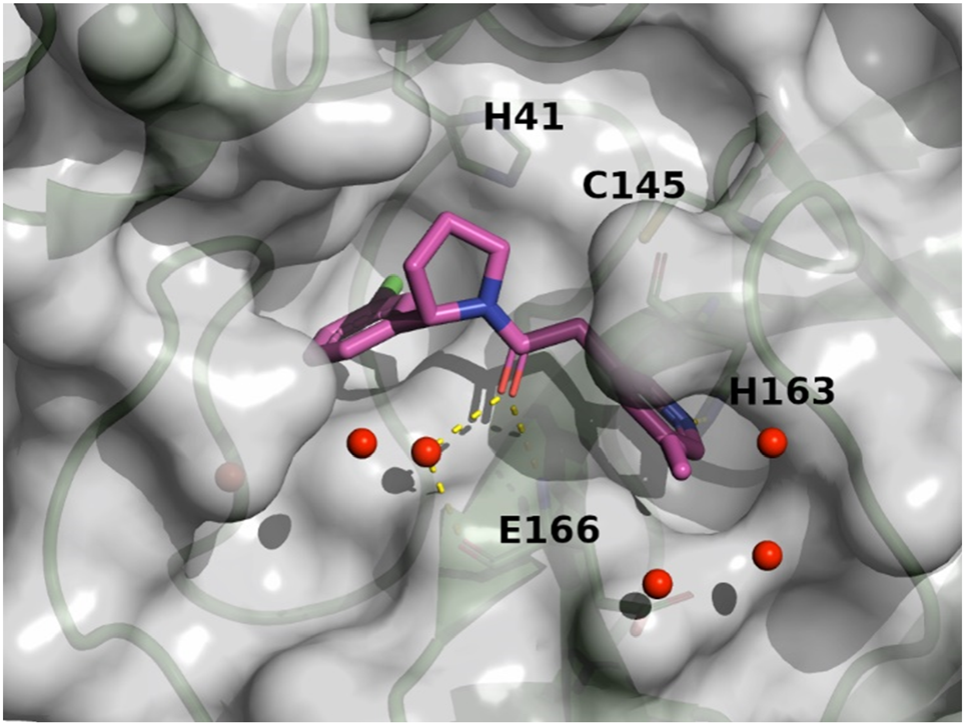
X-ray crystal structure of compound 7 (PDB code: 8r11) in the SARS-CoV-2 Mpro active site.

The X-ray structures of SARS-CoV-2 Mpro in complex with compounds 7 and 8 (PDB code 8r11 and 8r12 respectively) were solved and utilized to further optimise compound 8 into compound 11. The binding mode of compound 7 is depicted in Fig. 6. The following observation can be made with this figure, the *m*-methylpyridine sits in the S1 pocket where the pyridine nitrogen makes a key H-bond interaction with H163. The amide carbonyl makes a key interaction with the E166 backbone-NH and a water-mediated interaction with the E166 backbone-carbonyl. The *m*-chlorophenyl sits in the hydrophobic S2 pocket making face-to-edge aryl interaction with H41.

**Fig. 7:**
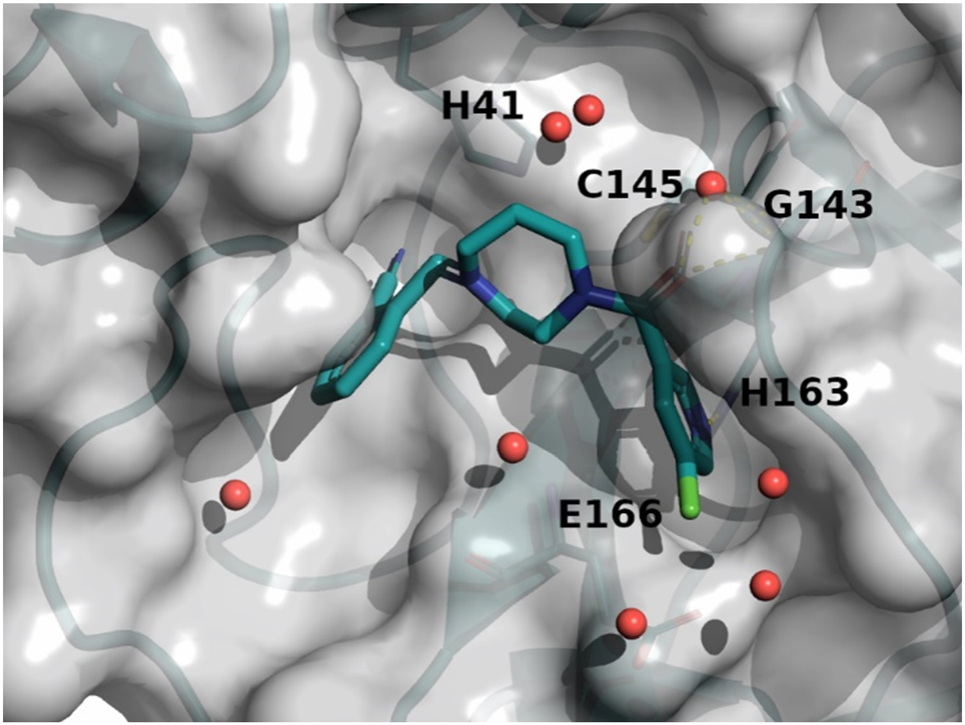
X-ray crystal structure of compound 8 (PDB code: 8r12) in the SARS-CoV-2 Mpro active site.

The binding mode of compound 8 is depicted in Fig. 7. The following observation can be made with this figure: the *m*-chloropyridine sits in the S1 pocket where the pyridine nitrogen makes a key H-bond interaction with H163. The amide carbonyl makes a key interaction with G143. The *m*-chlorophenyl sits in the hydrophobic S2 pocket making face-to-edge aryl interaction with H41.

The diazepane molecule (compound 8) was optimized following a structure-based approach (SBDD).

**Fig. 8:**
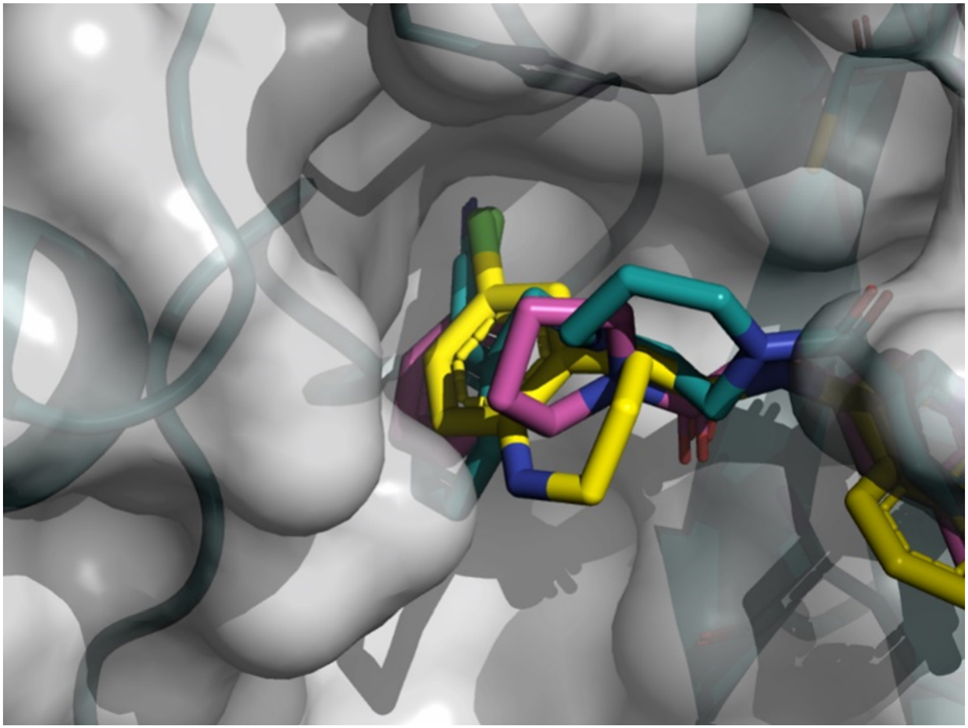
X-ray crystal structure of compound 8, 7 and COVID Moonshot EDJ-MED-92e193ae-1 superimposed in the SARS-CoV-2 Mpro active site.

As depicted in Fig. 8, the 3D structure observations allowed us to understand the importance of a chlorine atom in the S2 pocket. Indeed, for example, compound 7 (magenta) has an Mpro FRET IC_50_ of 5.6 μM or the COVID Moonshot compound EDJ-MED-92e193ae-1 (yellow) has an Mpro FRET IC_50_ of 0.230 μM for which available crystal structures were aligned with the crystal structure of compound 8 (cyan). This picture shows the possibility of replacing the cyano moiety of compound 8 with a chlorine in the *ortho* position. Furthermore, the nitrile bore by compound 8 should engage in polar interactions while the S2 pocket is known as a hydrophobic pocket.^36^ Therefore, the substitution by a chlorine atom should be beneficial as this latter should form hydrophobic interactions, hence the potency should be improved. This SBDD suggestion was then synthesized and led to compound 11 with an Mpro FRET IC_50_ of 1.3 μM (28-fold improvement).

**Fig. 9:**
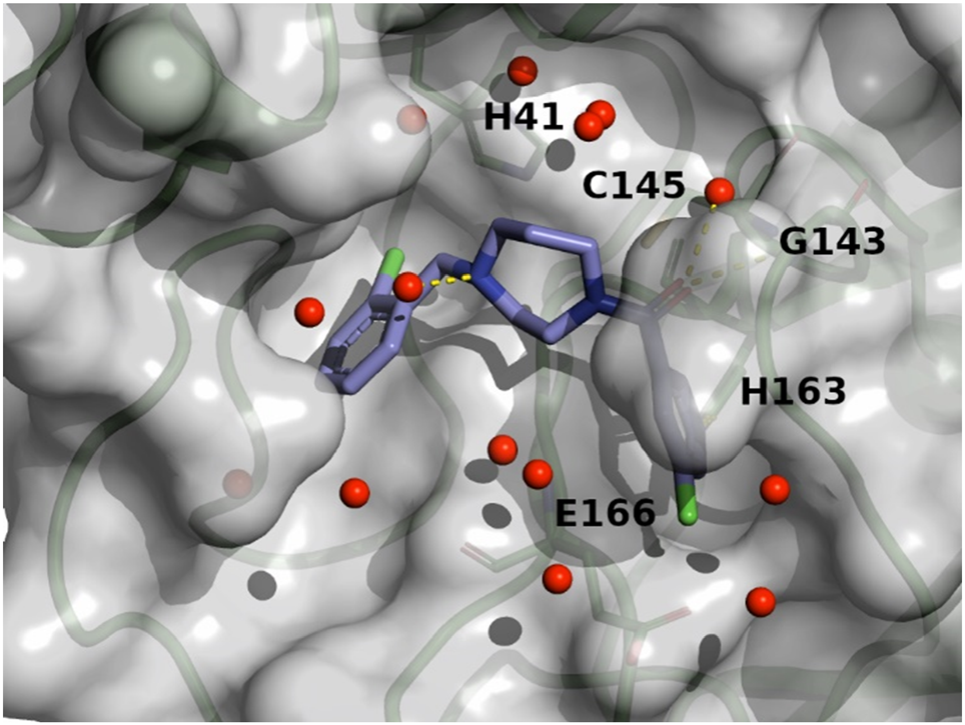
X-ray crystal structure of compound 11 (PDB code: 8r14) in the SARS-CoV-2 Mpro active site.

The crystal structure of compound 11 (PDB code: 8r14) underlines the relevance of the chlorine atom and is depicted in Fig. 9. The following observation can be made with this figure: the *m*-chloropyridine sits in the S1 pocket where the pyridine nitrogen makes a key H-bond interaction with H163. The amide carbonyl makes a key interaction with G143. The *m*-cyanophenyl sits in the hydrophobic S2 pocket making face-to-edge aryl interaction with H41.

The tetrahydroisoquinoline molecule (compound 10) was improved by a standard SAR approach which consisted of exploring a substitution (methyl) in position 5 of the pyridine as this position was substituted for all other hits (compounds 7, compound 8 and compound 9) of Fig. 5, and additionally, the SAR consisted of the synthesis of a chemical library of several bi-substituted tetra-hydroisoquinolines. This first round of optimization allowed a 28-fold improvement for the diazepane series to 1.3 μM and a 27-fold improvement for the tetrahydroisoquinoline series to 2.3 μM (compound 12).

**Fig. 10:**
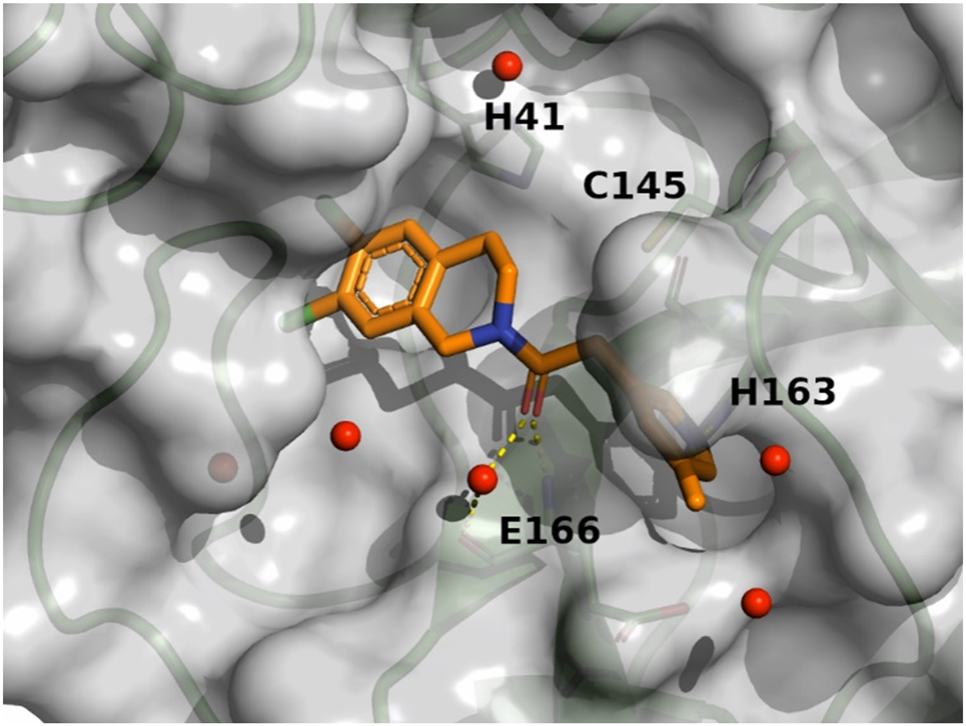
X-ray crystal structure of compound 12 (PDB code: 8r16) in the SARS-CoV-2 Mpro active site.

The crystal structure of compound 12 (PDB code: 8r16) is shown in Fig. 10. The following observation can be made with this figure: the *m*-methylpyridine sits in the S1 pocket where the pyridine nitrogen makes a key H-bond interaction with H163. The amide carbonyl makes a key interaction with E166 as well as water mediated interaction with G143. The two chlorine atoms of the tetrahydroisoquinoline sit in the hydrophobic S2 pocket.

Eventually, the AI generated hits followed-up by hit expansion, docking and a first round of optimization for compound 8 and 10 resulted in the identification of 3 novel Mpro inhibitors, a diazepane (compound 11), a pyrrolidine (compound 7) and a tetrahydroisoquinoline (compound 12) with IC_50_s ranging from 1.3 to 2.3 μM. Compound 9, while more potent than its queries (compound 4 and 5), was considered too similar to the compounds identified in the frame of the COVID Moonshot.

Our work showcases the potential of combining deep reinforcement learning for *de novo* drug design with additional computational chemistry techniques, as we successfully identified three novel Mpro inhibitors that display high potential for further development. We believe that our stepwise approach is key for slowly but surely identifying new inhibitors.

There are, however, several limitations and challenges that need to be addressed to improve the efficiency and effectiveness of RL-based *de novo* molecule design.

One of the main limitations is the similarity of the generated molecules with reference Mpro molecules. Since the retrained agent of our exploitation approach learns from existing molecules, it tends to generate molecules that resemble the training set. Additionally, both for the exploitation or exploration, the SMC scoring component certainly influenced the training of the agent towards the known Mpro chemical space. As a result, the generated molecules may not be sufficiently diverse or novel, leading to limited success in identifying new lead compounds.

To overcome this issue, training the generative model without the SMC scoring component in full exploration mode might constitute an interesting perspective or considering a docking scoring component instead of the SMC scoring components might also have the potential to lead to additional novelty.

Another significant challenge is the synthetic accessibility of the generated molecules. The models may generate molecules that are structurally complex or contain building blocks that are difficult to synthesize, making them impractical for further experimental evaluation. Despite the aid of Aizynthfinder in eliminating challenging or infeasible molecules, we failed to fully consider the true availability and cost of the building blocks for the interesting molecules we considered. Indeed, Aizynthfinder allows one to make its own collection of building blocks. This stock collection is then used by the tool when it recursively breaks down the molecule into available precursors. In our situation, we used the building block collection of aggregators (regrouping many vendors) which made the approach not fully effective. Indeed, the molecules were successfully considered synthesizable by Aizynthfinder but as the building blocks were available from a plethora of vendors it made it very difficult for one Contract Research Organization (CRO) to make a reasonable offer. Lesson learned: make an Aizynthfinder stock from a preferred vendor/CRO and not from an aggregator. Consequently, some of the compounds that were considered turned out to be excessively costly.

## Conclusion

In summary, we have used a *de novo* method for the generation of molecules with the goal of identifying new SARS-COV-2 Mpro inhibitors. The method uses a reinforcement learning approach that guides the generative model in creating desired molecules. It is based on the open-source code REINVENT 2.0, an AI tool for *de novo* drug design. The presented method is designed to generate molecules which contain key features of known Mpro inhibitors such as 3D pharmacophore/shape and privileged fragments. Additional computational chemistry techniques, used as post filters, such as molecule sanitization, QED, SMC, Jaccard distance, PheSA, ML affinity, Aizynthfinder, molecular diversity, molecular docking, and final consensus scoring, were employed to further enrich the generated molecules toward higher likelihood of being active against SARS-COV-2 Mpro.

We believe that the identification of SARS-CoV-2 Mpro inhibitors through reinforcement learning *de novo* design combined with 3D pharmacophore modeling, shape-based alignment, hit expansion, and molecular docking approaches represents a promising avenue for the identification of new starting points that could lead to new therapies for COVID-19. We are convinced that combining modern ML techniques together with state-of-the-art computational chemistry techniques has the potential to accelerate the identification of effective treatments for this severe disease.

The success of our workflow speaks for itself: six AI generated primary hits demonstrated weak to decent affinity. These molecules could be easily and quickly improved by hit expansion and showed novel Mpro chemotypes.

In our case study, we show that analogues picked out in the frame of the hit expansion and further prioritized by molecular docking led to the identification of three novel inhibitors. Two of them underwent a first round of optimization in the lab.

We then additionally report the identification of three novel SARS-COV-2 inhibitors displaying inhibitory activity ranging from 1.3 μM to 2.3 μM fulfilling our initial goal.

## Experimental section

### Datasets

• Reference Mpro inhibitors: a set of 225 Mpro inhibitors (FRET or FRET and RapidFire IC_50_ < 5 μM) was collected from various sources (ChEMBL, COVID Moonshot, and literature). The dataset contains compounds inhibiting SARS, SARS-CoV-2 and MERS Mpro. The dataset contains covalent and noncovalent inhibitors.

• ML dataset: ChEMBL and COVID Moonshot molecules with IC_50_ data (FRET and RapidFire assay data) were pooled together. For molecules with several measurements, a standard deviation was calculated together with the difference between the max and min value. Measurements showing a difference greater than 2 times standard deviation were discarded. The ML dataset was made up of 739 datapoints.

• DGM-TL dataset: a set of 225 Mpro inhibitors (FRET or FRET and RapidFire IC_50_ < 5 μM) previously collected was combined with 113 additional inhibitors (FRET or FRET and RapidFire 5 μM < IC_50_ < 15 μM) from various sources (ChEMBL, Covid Moonshot, and literature).

• COVID Moonshot FRET activity dataset: the dataset was downloaded from https://covid.postera.ai/covid/activity_data and contains all measured compounds (24.03.2021) by FRET assay for which an IC_50_ value is available. The dataset contains 940 molecules.

• Privileged fragments: a list of privileged fragments was assembled as described in the following SMC scoring component validation section.

• Docking validation set: 25 “active” (IC_50_ ≤ 0.5 μM) non-covalent inhibitors and 25 “inactive” (IC_50_ ≥ 99 μM) compounds were randomly picked from the COVID Moonshot dataset and combined.

### Docking with GOLD

To get the input molecules ready for docking, their three-dimensional (3D) structure needs to be determined. This step was accomplished using the OEChem package.^37^ Similarly, the proteins were prepared with the same package. This process involved removing any atoms not part of the protein, reconstructing missing side chains and loops, and adding hydrogen atoms to the protein structure. The ligands, which are smaller molecules that bind to proteins, were retrieved from Protein Data Bank (PDB) files using the Biopython package. These ligands were then processed using the rdkit module, a process that involved removing any salts and neutralizing charges in the ligands.

The idea behind the following workflow is that compounds with similar 3D structures would perturb the binding pocket in the same way. To identify a reference compound that closely resembles the ligands, the PheSA program was used. PheSA was employed to calculate the similarity between all the ligands to evaluate and the active conformations of reference compounds.

For the docking process, hydrogen atoms were also added to the input molecules. The docking itself was conducted using the GOLD docking program, which allows for automatic non-covalent docking. This procedure was integrated into a script for efficiency. The docking utilized both the prepared PDB files of the ligands most similar to the reference compounds and the prepared input molecules.

### Consensus scoring (CS)

It has been reported that CS, which combines multiple scoring functions in binding affinity estimation, leads to higher hit rates in virtual library screening studies.^38^ It has also been demonstrated that consensus scoring outperforms, for statistical reason, any single scoring.^39^

A consensus scoring was developed taking PheSA, SeeSAR & Gold docking, and SMC scores into account.

• The SMC and PheSA scores were already normalised from 0 to 1 so the scores were kept unchanged.

• ChemScore was normalized from 0 to 1.

• SeeSAR Hyde_IC_50_ was converted to pIC_50_ = −log(Hyde_IC_50_) then the score was normalized from 0 to 1.

• The CS was calculated with the following equation:
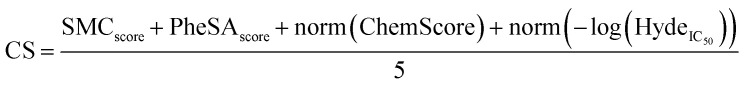
The CS score was used to make the final selection of compounds to be purchased.

### Synthetic details for the preparation of compounds

Commercially available starting materials were used as received without further purification. Flash column chromatography was performed using Biotage SNAP cartridges (10–340 g) and elution was with a Biotage Isolera system. Merck pre-coated thin layer chromatography (TLC) plates were used for TLC analysis. Final compounds were purified to >95% purity (UV and NMR) by reverse phase preparative HPLC using a Waters XBridge column (10 μm, 75 × 30 mm). Conditions: MeCN [eluent A]; water + 0.5% NH_4_OH (25% aq.) [eluent B]; gradient: 90% B → 5% B over 6.5 min (flow: 75 mL min^−1^). Detection: UV/vis + MS. Racemates can be separated into their enantiomers by chiral HPLC using a ChiralPaK IC column (5 μm, 250 × 4.6 mm). Conditions: heptane + 0.05% DEA [eluent A]; EtOH + 0.05% DEA [eluent B]; isocratic elution with 50% eluent B (flow: 1 mL min^−1^), or a CHIRALCEL OZ-H column (5 μm, 250 × 4.6 mm). Conditions: CO_2_ [eluent A]; EtOH + 0.1% DEA [eluent B]; isocratic elution with 50% eluent B (flow: 4 mL min^−1^).

Mass spectrometry data were recorded by one of the following methods:

#### LC–MS with acidic conditions

Method A: Agilent 1100 series with mass spectrometry detection (MS: Finnigan single quadrupole). Column: Zorbax SB-aq (3.5 μm, 4.6 × 50 mm). Conditions: MeCN [eluent A]; water + 0.04% TFA [eluent B]. Gradient: 95% B → 5% B over 1.5 min (flow: 4.5 mL min^−1^). Detection: UV/vis + MS.

Method B: Agilent 1100 series with mass spectrometry detection (MS: Finnigan single quadrupole). Column: Waters XBridge C18 (2.5 μm, 4.6 × 30 mm). Conditions: MeCN [eluent A]; water + 0.04% TFA [eluent B]. Gradient: 95% B → 5% B over 1.5 min (flow: 4.5 mL min^−1^). Detection: UV/vis + MS.

#### LC–MS with basic conditions

Method C: Agilent 1100 series with mass spectrometry detection (MS: Finnigan single quadrupole). Column: Zorbax Extend C18 (5 μm, 4.6 × 50 mm). Conditions: MeCN [eluent A]; 13 mmol L^−1^ NH_3_ in water [eluent B]. Gradient: 95% B → 5% B over 1.5 min (flow: 4.5 mL min^−1^). Detection: UV/vis + MS.

Method D: Agilent 1100 series with mass spectrometry detection (MS: Finnigan single quadrupole). Column: Waters XBridge C18 (5 μm, 4.6 × 50 mm). Conditions: MeCN [eluent A]; 13 mmol L^−1^ NH_3_ in water [eluent B]. Gradient: 95% B → 5% B over 1.5 min (flow: 4.5 mL min^−1^). Detection: UV/vis + MS.

LC-HRMS parameters were the following: analytical pump Waters Acquity binary, Solvent Manager, MS, SYNAPT G2 MS, source temperature of 150 °C, desolvation temperature of 400 °C, desolvation gas flow of 400 L h^−1^; cone gas flow of 10 L h^−1^, extraction cone of 4 RF; lens 0.1 V; sampling cone 30; capillary 1.5 kV; high resolution mode; gain of 1.0, MS function of 0.2 s per scan, 120–1000 amu in full scan, centroid mode. Lock spray: leucine enkephalin, 2 ng mL^−1^ (556.2771 Da), scan time of 0.2 s with interval of 10 s and average of 5 scans; DAD: Acquity UPLC PDA detector. Column was an Acquity UPLC BEH C18 1.7 μm, 2.1 mm × 50 mm from Waters, thermostated in the Acquity UPLC column manager at 60 °C. Eluents were the following: water + 0.05% formic acid; B, acetonitrile + 0.05% formic acid. Gradient was 2–98% B over 3.0 min. Flow was 0.6 mL min^−1^. Detection was at UV 214 nm.

^1^H NMR spectra were recorded on a Bruker (400 or 500 MHz) spectrometer in the indicated deuterated solvent. Chemical shifts are reported in ppm relative to solvent peaks as the internal reference.

### Compound 1; (4-(2,4-dichlorobenzyl)piperazin-1-yl)(6-fluoroisoquinolin-4-yl)methanone

Commercially available material. LC-HRMS: *t*_R_ = 0.875 min; [M + H]/*z* = 418.0889 found = 418.2800.

### Compound 2; (4-(3,5-dichloro-2-(difluoromethoxy)benzyl) piperazin-1-yl)(pyridin-3-yl) methanone

Commercially available material. LC-HRMS: *t*_R_ = 0.916 min; [M + H]/*z* = 416.0744 found = 416.2700.

### Compound 3; (4-(2,3-dichlorobenzyl)piperazin-1-yl)(pyridin-3-yl)methanone

Commercially available material. LC-HRMS: *t*_R_ = 0.631 min; [M + H]/*z* = 350.0827 found = 350.2200.

### Compound 4; 1-(2-chlorobenzyl)-3-(6-chloroisoquinolin-4-yl)imidazolidin-2-one

Commercially available material. LC-HRMS: *t*_R_ = 1.102 min; [M + H]/*z* = 372.0670 found = 372.2600.

### Compound 5; 1-(2,4-dichlorobenzyl)-3-(isoquinolin-4-yl)imidazolidin-2-one

Commercially available material. LC-HRMS: *t*_R_ = 1.037 min; [M + H]/*z* = 372.0670 found = 372.2300.

### Compound 6; *rac*-(*R*)-5,6-dichloro-*N*-(4,6-difluoro-1*H*-benzo[*d*]imidazol-1-yl)-2,3-dihydrobenzofuran-3-carboxamide

Commercially available material. LC-HRMS: Not available.

### Compound 13; *rac*-(*R*)-4,5-dichloro-*N*-(1-(cyclopropylmethyl)-1*H*-imidazol-5-yl)-2,3-dihydrobenzofuran-3-carboxamide

Commercially available material. LC-HRMS: *t*_R_ = 0.616 min; [M + H]/*z* = 352.0620 found = 352.2000.

### Compound 14; *rac*-(*R*)-*N*-(1-(3-chloro-4-fluorophenyl)-3-hydroxypropyl)-*N*-methyl-2-(2,4,5-trifluorophenyl)acetamide

Commercially available material. LC-HRMS: *t*_R_ = 1.104 min; [M + H]/*z* = 390.0884 found = 390.1900.

### Compound 15; *N*-(4,5-dichloro-2-(1*H*-1,2,4-triazol-1-yl)benzyl)-2-(3,5-difluorophenyl)acetamide

Commercially available material. LC-HRMS: *t*_R_ = 1.043 min; [M + H]/*z* = 397.0434 found = 397.1400.

### Compound 16; 1-(3,4-dichlorophenyl)-*N*-(4,5-difluoro-2-(pyridin-4-yl)benzyl)cyclopropane-1-carboxamide

Commercially available material. LC-HRMS: *t*_R_ = 1.126 min; [M + H]/*z* = 433.0686 found = 433.2900.

### Compound 17; (4-(3,5-dichloro-2-fluorobenzyl)piperazin-1-yl)(furan-3-yl)methanone

Commercially available material. LC-HRMS: *t*_R_ = 0.854 min; [M + H]/*z* = 357.0573 found = 357.2000.

### Compound 18; 1-(4-(3,4-dichlorobenzyl)piperazin-1-yl)-2-(1*H*-1,2,4-triazol-1-yl)ethan-1-one

Commercially available material. LC-HRMS: *t*_R_ = 0.526 min; [M + H]/*z* = 354.0888 found = 354.2300.

### Compound 19; *N*-(3,4-dichlorobenzyl)-2-(1*H*-tetrazol-1-yl) acetamide

Commercially available material. LC-HRMS: *t*_R_ = 0.854 min; [M + H]/*z* = 286.0262 found = 286.1700.

### Compound 20; 2-(4-cyclopropyl-1*H*-1,2,3-triazol-1-yl)-*N*-(3,4-dichlorobenzyl)acetamide

Commercially available material. LC-HRMS: *t*_R_ = 0.966 min; [M + H]/*z* = 325.0623 found = 325.2100.

### Compound 21; 6,8-dichloro-*N*-(1-ethyl-1*H*-imidazol-5-yl)-3-methylchromane-4-carboxamide

Commercially available material. LC-HRMS: *t*_R_ = 0.675 min; [M + H]/*z* = 354.0776 found = 354.4000.

### Compound 22; *rac*-(*R*)-4-chloro-*N*-(2*H*-pyrazolo[3,4-*c*]pyridin-4-yl)bicyclo[4.2.0]octa-1(6),2,4-triene-7-carboxamide

Commercially available material. LC-HRMS: *t*_R_ = 0.656 min; [M + H]/*z* = 299.0700 found = 299.2500.

### Compound 7; (*S*)-1-(2-(3-chlorophenyl)pyrrolidin-1-yl)-2-(5-methylpyridin-3-yl)ethan-1-one

DIPEA (0.0524 mL, 0.3 mmol, 3 eq.) was added to a solution of 2-(5-methylpyridin-3-yl)acetic acid hydrochloride (0.1 mmol, 1 eq.) in DMF p.a (0.20 mL), followed by a solution of (2*S*)-2-(3-chlorophenyl)pyrrolidine (20.7 mg, 0.105 mmol, 1.05 eq.) and DIPEA (0.0349 mL, 0.2 mmol, 2 eq.) in DMF p.a (0.4 mL). Finally, a 0.5 M HATU stock solution was added to DMF (220 μL, 0.11 mmol, 1.1 eq.) and stirred overnight at RT. The product was isolated by basic preparative HPLC (29 mg, 90% yield). LC-HRMS: *t*_R_ = 0.633 min; [M + H]/*z* = 315.1264 found = 315.4000; ^1^H NMR: presence of 2 stable conformational isomers in DMSO at RT, ratio 2 : 1, *δ*H(500 MHz, DMSO): 8.28 (0.67H, dd, *J* = 2.0, 0.9 Hz), 8.24 (0.67H, d, *J* = 2.1 Hz), 8.19 (0.33H, dd, *J* = 2.1, 0.9 Hz), 7.95 (0.33H, d, *J* = 2.0 Hz), 7.45–7.18 (3.34H, m), 7.17–7.08 (1.66H, m), 5.28 (0.33H, dd, *J* = 8.1, 2.3 Hz), 5.02 (0.67H, dd, *J* = 8.1, 3.1 Hz), 3.88–3.77 (1.33H, m), 3.74–3.62 (1.67H, m), 3.59–3.48 (0.67H, m), 3.18–3.14 (0.33H, m), 2.42–2.36 (0.33H, m), 2.28 (2H, d, *J* = 0.7 Hz), 2.26–2.23 (0.33H, m), 2.20 (1H, d, *J* = 0.8 Hz), 1.97–1.67 (3.34H, m).

### Compound 8; 2-((4-(5-chloronicotinoyl)-1,4-diazepan-1-yl)methyl)benzonitrile

#### Step 1: *tert*-butyl 4-(5-chloronicotinoyl)-1,4-diazepane-1-carboxylate

*N*-(*t*-Butyloxycarbonyl)-homopiperazine hydrochloride (947 mg, 4 mmol, 1 eq.), 5-chloronicotinic acid (715 mg, 4.4 mmol, 1.1 eq.) and DIPEA (2.05 mL, 12 mmol, 3 eq.) were dissolved in DCM (40 mL). HATU (1725 mg, 4.4 mmol, 1.1 eq.) was added and the mixture was stirred at RT for 30 min. Aq. NaHCO_3_ was added, and the product was extracted 2× with DCM. The combined organic phases were dried over MgSO_4_ and concentrated under reduced pressure. The product was isolated by flash chromatography (1.565 g, 115% yield). LC–MS D: *t*_R_ = 0.82 min; [M + H]+ = 340.17.

#### Step 2: (5-chloropyridin-3-yl)(1,4-diazepan-1-yl)methanone hydrochloride

*Tert*-butyl 4-(5-chloronicotinoyl)-1,4-diazepane-1-carboxylate (1565 mg, 3.9 mmol, 1 eq.) was dissolved in MeOH (30 mL) and HCl 4 M in dioxane (5 mL, 20 mmol, 5.134 eq.) was added. The mixture was stirred at RT for 4 h. Some extra HCl (2 eq.) was added, and the mixture was stirred at RT for 1 h to reach full completion of the reaction. The solvents were evaporated under reduced pressure. A white solid was obtained. LC–MS D: *t*_R_ = 0.48 min; [M + H]+ = 240.13.

#### Step 3: 2-((4-(5-chloronicotinoyl)-1,4-diazepan-1-yl)methyl)benzonitrile

(5-Chloropyridin-3-yl)(1,4-diazepan-1-yl)methanone hydrochloride (102 mg, 0.3 mmol, 1 eq.), 2-cyanobenzaldehyde (48.2 mg, 0.36 mmol, 1.2 eq.) and DIPEA (0.154 mL, 0.9 mmol, 3 eq.) were dissolved in DCM (3 mL). Sodium triacetoxyborohydride 97% (167 mg, 0.75 mmol, 2.5 eq.) was added and the mixture was stirred overnight at RT. Saturated aqueous NaHCO_3_ (0.5 mL) was added, the organic phase was filtered through a phase separator and evaporated in a Genevac overnight. The product was isolated by RP prep HPLC, basic condition, polar gradient. LC-HRMS: *t*_R_ = 0.527 min; [M + H]/*z* = 355.1264 found = 355.4000.

### Compound 9; (*S*)-5-(5-chloro-2-fluorobenzyl)-3-(5-fluoro pyridin-3-yl)imidazolidine-2,4-dione

Commercially available material. LC-HRMS: *t*_R_ = 0.926 min; [M + H]/*z* = 338.0508 found = 338.4000.

### Compound 10; 1-(5,7-dichloro-3,4-dihydroisoquinolin-2(1*H*)-yl)-2-(pyridin-3-yl)ethan-1-one

Commercially available material. LC-HRMS: *t*_R_ = 0.746 min; [M + H]/*z* = 321.0561 found = 321.3000.

### Compound 11; (4-(2-chlorobenzyl)-1,4-diazepan-1-yl)(5-chloropyridin-3-yl)methanone

#### Step 1: *tert*-butyl 4-(5-chloronicotinoyl)-1,4-diazepane-1-carboxylate

*N*-(*t*-Butyloxycarbonyl)-homopiperazine hydrochloride (947 mg, 4 mmol, 1 eq.), 5-chloronicotinic acid (715 mg, 4.4 mmol, 1.1 eq.) and DIPEA (2.05 mL, 12 mmol, 3 eq.) were dissolved in DCM (40 mL). HATU (1725 mg, 4.4 mmol, 1.1 eq.) was added and the mixture was stirred at RT for 30 min. Aq. NaHCO_3_ was added, and the product was extracted 2× with DCM. The combined organic phases were dried over MgSO_4_ and concentrated under reduced pressure. The product was isolated by flash chromatography (1.565 g, 115% yield). LC–MS D: *t*_R_ = 0.82 min; [M + H]+ = 340.17.

#### Step 2: (5-chloropyridin-3-yl)(1,4-diazepan-1-yl)methanone hydrochloride

*T*ert-butyl 4-(5-chloronicotinoyl)-1,4-diazepane-1-carboxylate (1565 mg, 3.9 mmol, 1 eq.) was dissolved in MeOH (30 mL) and HCl 4 M in dioxane (5 mL, 20 mmol, 5.134 eq.) was added. The mixture was stirred at RT for 4 h. Some extra HCl (2 eq.) was added, and the mixture was stirred at RT for 1 h to reach full completion of the reaction. The solvents were evaporated under reduced pressure. A white solid was obtained. LC–MS D: *t*_R_ = 0.48 min; [M + H]+ = 240.13.

#### Step 3: (4-(2-chlorobenzyl)-1,4-diazepan-1-yl)(5-chloropyridin-3-yl)methanone

(5-Chloropyridin-3-yl)(1,4-diazepan-1-yl)methanone (34.0 mg, 0.10 mmol) was dissolved in a mixture of DCM (9.5 mL) and DIPEA (0.50 mL). 2-Chlorobenzaldehyde (0.12 mmol, 1.2 eq.) was added, followed by sodium triacetoxyborohydride 97% (55.8 mg, 0.25 mmol, 2.5 eq.). The mixtures were stirred at RT overnight. Aqueous NaHCO_3_ and DCM were added, the products was extracted 3× with DCM. Finally, the product was isolated by reverse phase preparative HPLC, polar gradient. LC-HRMS: *t*_R_ = 0.516 min; [M + H]/*z* = 364.0983 found = 364.2700; ^1^H NMR: *δ*H(500 MHz, DMSO): 8.70 (1H, dd, *J* = 7.0, 2.4 Hz), 8.57 (1H, dd, *J* = 9.3, 1.8 Hz), 8.02 (1H, dt, *J* = 18.1, 2.1 Hz), 7.57–7.39 (2H, m), 7.38–7.23 (2H, m), 3.73 (1H, s), 3.69 (1H, s), 3.69–3.64 (2H, m), 3.41 (2H, td, *J* = 5.7, 2.8 Hz), 2.82–2.76 (1H, m), 2.74–2.66 (2H, m), 2.66–2.60 (1H, m), 1.89–1.81 (1H, m), 1.78–1.69 (1H, m).

### Compound 12; 1-(6,7-dichloro-3,4-dihydroisoquinolin-2(1*H*)-yl)-2-(5-methylpyridin-3-yl)ethan-1-one

DIPEA (0.0524 mL, 0.3 mmol, 3 eq.) was added to a solution of 6,7-dichloro-1,2,3,4-tetrahydroisoquinoline hydrochloride (0.1 mmol, 1 eq.) in DMF p.a (0.20 mL), followed by a solution of 2-(5-methylpyridin-3-yl)acetic acid hydrochloride (20.7 mg, 0.105 mmol, 1.05 eq.) and DIPEA (0.0349 mL, 0.2 mmol, 2 eq.) in DMF p.a (0.4 mL). Finally, a 0.5 M HATU stock solution was added to DMF (220 μL, 0.11 mmol, 1.1 eq.) and stirred overnight at RT. The product was isolated by basic preparative HPLC (30 mg, 90% yield). LC-HRMS: *t*_R_ = 0.738 min; [M + H]/*z* = 335.0718, found = 335.2100; ^1^H NMR: presence of 2 stable conformational isomers in DMSO at RT, ratio 6 : 4, *δ*H(500 MHz, DMSO): 8.30–8.22 (2H, m), 7.56 (0.6H, s), 7.50 (0.6H, s), 7.49 (0.4H, s), 7.48 (0.4H, s), 7.45 (0.6H, s), 7.42 (0.4H, s), 4.75 (0.8H, s), 4.62 (1.2H, s), 3.81 (1.2H, s), 3.80 (0.8H, s), 3.76 (1.2H, t, *J* = 5.9 Hz), 3.67 (0.8H, t, *J* = 6.0 Hz), 2.83 (1.2H, t, *J* = 5.9 Hz), 2.77 (0.8H, t, *J* = 6.0 Hz), 2.27 (1.8H, s), 2.25 (1.2H, s).

## Conflicts of interest

The authors declare no conflicts of interest.

## Supplementary Material

MD-015-D4MD00106K-s001

MD-015-D4MD00106K-s002
