# Supplementary material for: Identification of SARS-CoV-2 Mpro inhibitors through deep reinforcement learning for de novo drug design and computational chemistry approaches
Source: RSC Med Chem. 2024 Apr 29;15(6):2146–59. doi: 10.1039/d4md00106k (PMC11187573; doi:10.1039/d4md00106k)
Supplement: MD-015-D4MD00106K-s002 [file MD-015-D4MD00106K-s002.pdf]

Please do not adjust margins

ARTICLE

Supplementary Information

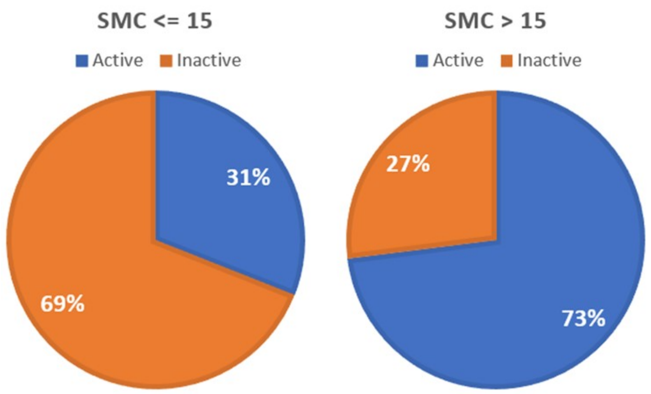

Figure 1. The pie charts represent the proportion of active versus inactive molecule for molecules with either a SMC <= 15 (n=815) or a SMC > 15 (n=125)

$$\mu_A(x) = \begin{cases} 0, & x < a \\ \frac{x-a}{b-a}, & a \le x \le b \\ 1, & x > b \end{cases}$$

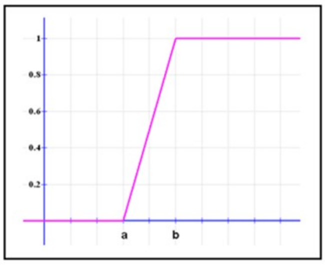

F

igure 2. Membership trapezoidal scoring function, Substructure match count on the x axis and score on the y axis, a = 0 and b = 15

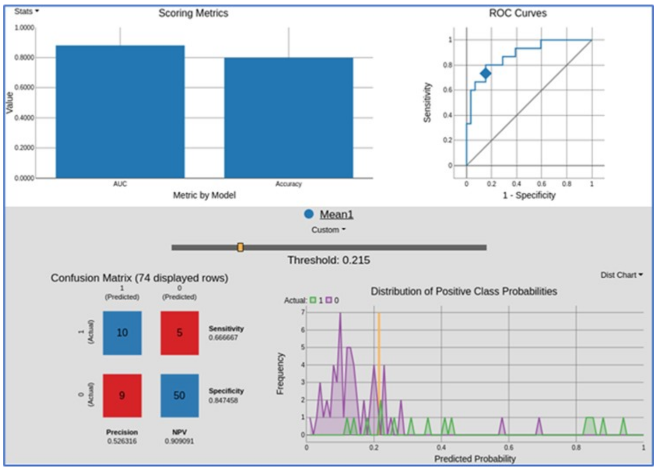

Figure 3. The figure was obtained from the Knime Binary Classification Inspector node. The view display performance of the RF-FPs, XGBoost-FPs and GCNN model ensemble on the validation set. On the top left the model's statistic bar chart: AUC and overall accuracy. On the top right the ROC curve. The confusion matrix on the bottom left and the distribution of positive class probabilities on the bottom right. The statistical metrics adapt according to the threshold value.

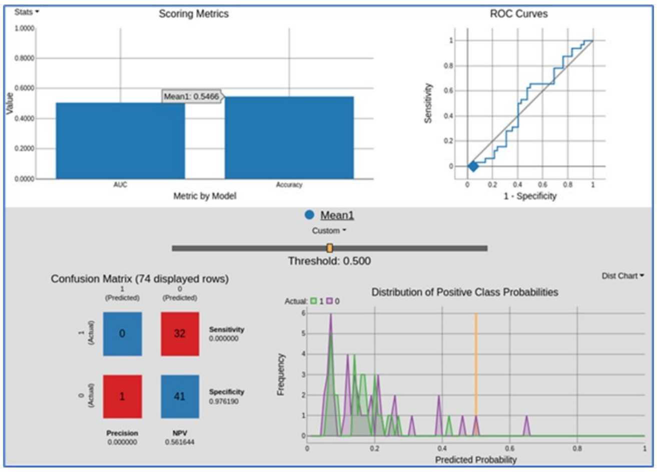

Figure 4. The figure was obtained from the Knime Binary Classification Inspector node. The view display performance of the RF-FPs, XGBoost-FPs and GCNN model ensemble on the test set. The view display performance of ML model predictions. On the top left the model's statistic bar chart: AUC and overall accuracy. On the top right the ROC curve. The confusion matrix on the bottom left and the distribution of positive class probabilities on the bottom right. The statistical metrics adapt according to the threshold value.

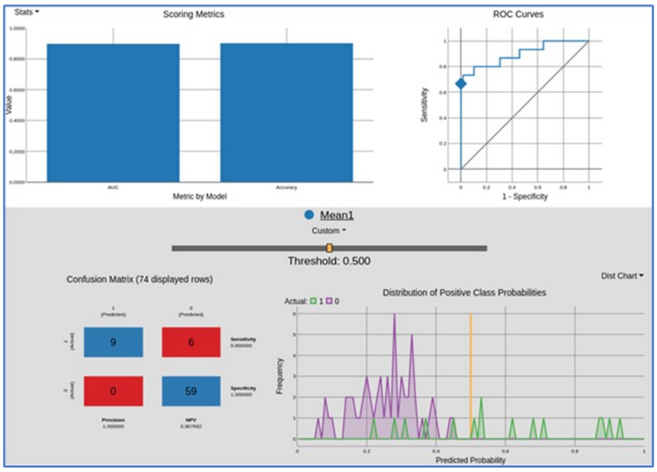

Figure 5. The view display performance of the RF-E3FP, XGBoost-E3FP model ensemble on the validation set. The view display performance of ML model predictions. On the top left the model's statistic bar chart: AUC and overall accuracy. On the top right the ROC curve. The confusion matrix on the bottom left and the distribution of positive class probabilities on the bottom right. The statistical metrics adapt according to the threshold value.

Please do not adjust margins

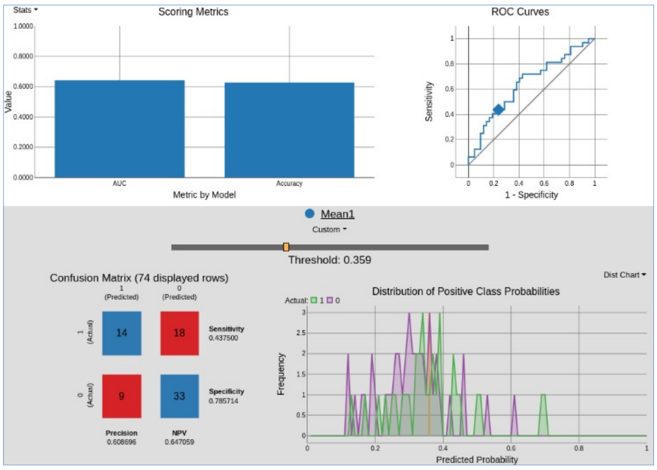

Figure 6. The view display performance of the RF-E3FP, XGBoost-E3FP model ensemble on the test set. The view display performance of ML model predictions. On the top left the model's statistic bar chart: AUC and overall accuracy. On the top right the ROC curve. The confusion matrix on the bottom left and the distribution of positive class probabilities on the bottom right. The statistical metrics adapt according to the threshold value.

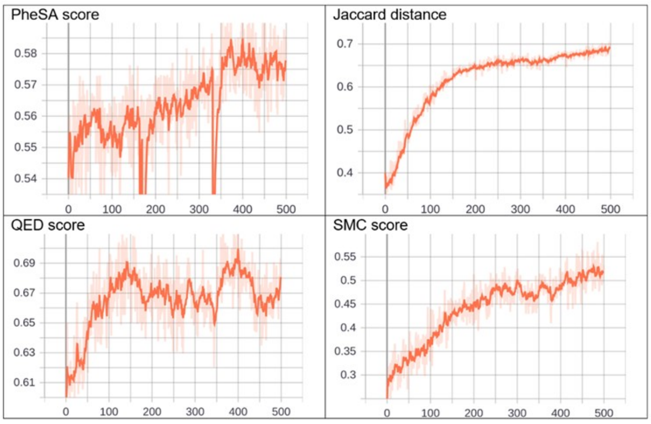

Figure 7. Tensorboard shows how the scores change over every epoch for the exploitation mode

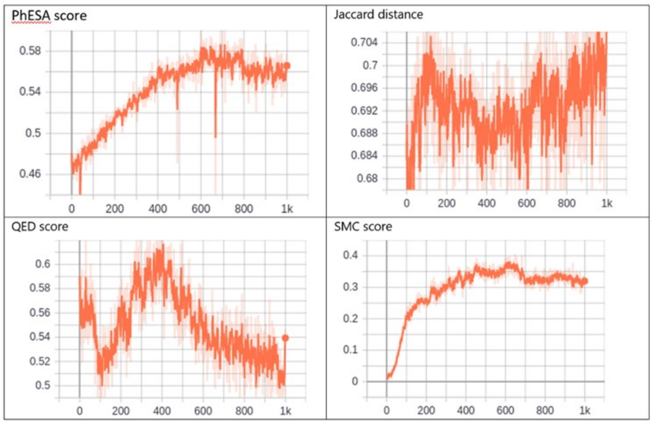

Figure 8. Tensorboard shows how the scores change over every epoch for the exploration mode

Fluorescence resonance energy transfer (FRET)-based Mpro proteolytic activity assay

The enzymatic activity of the recombinant SARS-CoV-2 main protease Mpro was determined by a fluorescence resonance energy transfer (FRET) assay using a custom synthesized peptide substrate with (7-Methoxycoumarin-4-yl)acetyl [MCA] as fluorophore and 2,4-Dinitrophenyl [DNP] as fluorescence quencher: MCA-Ala-Val-Leu-Gln-Ser-Gly-Phe-Arg-Lys(Dnp)-Lsy-NH2-trifluoroacetate salt (Bachem AG, Bubendorf CH). This peptide substrate amino acid sequence corresponds to the nsp4/nsp5 (Mpro) cleavage site. A substrate stock solution (10 mM) was prepared in 100 % DMSO. 40 µL of a 4 µM substrate solution prepared in H2O/TWEEN-20 0.01%) was added to a solution (40 µL) containing Mpro to start the enzymatic reaction. The final concentrations of the assay reaction ingredients (80 µl) are 5 nM [E] Mpro, 2 µM [S] peptide substrate (Km 3.17 µM), 1 mM DTT, 0.5 mM EDTA, 1.2 % DMSO, 0.01 % TWEEN-20, 25 mM TRIS pH 7.4. Mpro was diluted (10 nM) from aliquotes stored as stock solution (512 µM, -80 °C, storage buffer) in Mpro assay buffer (50 mM TRIS pH 7.4, 1 mM EDTA, 2 mM DTT, 0.01 % TWEEN-20). The rate of Mpro enzymatic activity (v) was determined by monitoring the increase in fluorescence intensity of reactions at room temperature in black microplates (NUNc 384-well F-bottom) with an Infinite M-1000 plate reader (Tecan) using 325 nm and 400 nm as wavelengths for excitation and emission, respectively. Test compounds were dissolved in DMSO and screened first at a 25 µM. 3-fold serial dilutions (125 µM – 6.35 nM) of small molecule test compounds are added to determine inhibitory potency. IC50 is determined by an in-house evaluation tool (IC50 Studio with 4-parametric fitting, Hill-equation).

Small molecule compounds showing putative inhibitory activity were tested in a separate assay for quenching potential of fluorescence emitted by the MCA fluorophore (Bioquest) to identify possible false positives.

Fluorimetric human liver Cathepsin L (hCatL) activity assay

To determine the effect of small molecule test compounds on the enzymatic activity of human Cathepsin L a fluorescence-based assay has been implemented according to a published protocol.[40]

Cathepsin L from human liver and the fluorogenic peptidomimetic substrate Z-Phe-Arg7-amido-4-methylcoumarin hydrochloride (Z-FR-AMC) were purchased from SIGMA (#219402, #C9521). The Cathepsin L enzyme buffer consisted of 50 mM Tris pH 6.5, 5 mM EDTA, 200 mM NaCl, 2 mM DTT. The hCatL enzymatic reaction was initiated by adding 40 µL of a solution containing the substrate at 4 µM (50 mM Tris pH 6.5, 5 mM EDTA, 200 mM NaCl, 0.005 % triton-X-100) to 40 µL solution consisting of enzyme in assay buffer (50 mM Tris pH 6.5, 5 mM EDTA, 200 mM NaCl , 2 mM DTT, 1 nM Ca). Test compounds dissolved in DMSO were dispensed as 2-fold serial dilutions (125 µM – 244 nM) to black well assay plates (384 well, NUNC).

This journal is © The Royal Society of Chemistry 20xx

Please do not adjust margins

Please do not adjust margins

Please do not adjust margins

Final assay reaction mixtures consisted of Cathepsin L 0.5 nM [E], Z-Phe-Arg7-amido-4-methylcoumarin [S], [cpds] 125 – 0.214 µM or 50 – 0.0977 µM, 50 mM Tris-HCL pH 6.5, 200 mM NaCl, DTT 1 mM, 2.5 mM EDTA, Triton-X100 0.0005%, DMS0 1.27 %.

Fluorescence emitted by AMC fluorophore liberated by hCatL cleavage of the substrate was measured with a Tecan infinite M-1000 plate reader with filters for excitation at 360/40 nm and emission at 460/40 nm at RT immediately after initiating the reaction t0 and 35 min incubation at RT.

Leupeptin (SIGMA L5793) a validated natural protease inhibitor shows the expected biochemical potency in this assay with an IC50 value of around 2 nM.

Cloning, protein expression and purification of SARS-CoV-2 Mpro

DNA encoding a recombinant fusion protein composed of SUMO with a N-terminal hexa-histidine tag and Mpro (NC_045512.2, Nsp5, YP_009742612, Wuhan-Hu-1) was codon optimized for expression in E. coli and synthesized at Genscript. The synthetic DNA was cloned into pET29a(+)::[NdeI, BamHI] (Genscript) and transformed into BL21(DE3) cells. The fusion protein was expressed overnight (Luria broth medium, 25 µg/ml Kanamycin) at 18 ºC after induction with 0.5 mM isopropyl-b-d-thiogalactoside IPTG at OD600~0.7. Overnight cultures were spun down and recovered cell paste was stored at -70 ºC. 12 g cell paste was resuspended in buffer (20 mM Tris-HCl, pH 7.8; 150 mM NaCl, 5 mM imidazole) and treated with lysozyme (0.1mg/ml; 30 min) and benzonase (2500 Units, 10 mM MgCl2; 15 min, RT). Bacterial cells were lysed by high pressure homogenization (29008 psi, Microfluidics MP110P, DIXC H10Z) and centrifuged 30 min at 16000 rpm (Fiberlite F21-8x50y). The hexa-histidine SUMO-Mpro fusion protein was purified by immobilized metal affinity chromatography (IMAC) with a HisTrap column (5 ml, Cytiva) connected to a FPLC Äkta purifier 100 system. His-tagged SUMO_Mpro fusion protein was eluted with a linear gradient of increasing imidazole concentration (elution buffer, 0-100 %, 20 column volumes, 20 mM Tris-HCl pH 7.8, 150 mM NaCl, 500 mM imidazole). Eluate fractions containing the target protein were combined and concentrated (Amicon, 10 kDa). The fusion protein was treated with SUMO protease (Sigma SAE0067, 5 U/ mg target protein) to liberate Mpro with authentic N-(Ser1) and C- termini (Q306)-C. The mixture of SUMO protease cleavage products was dialyzed overnight (4 ºC, 4 L, 20 mM Tris-HCl, 150 mM NaCl, Slide-A-Lyzer® cassette, 10 kDa, Thermo Scientific). The hexa-histidine tagged SUMO protein was separated from non-tagged authentic Mpro present in the dialysate by IMAC and collecting Mpro in the flow through. Mpro was further purified by size exclusion chromatography (SEC, Hiload 26/60 Superdex 200) with storage buffer (20 mM Tris-HCl, 150 mM NaCl, 1 mM TCEP, 1 mM EDTA). The SEC elution volume for Mpro indicated a dimer as the oligomeric state. Pure (97 %, LC MS) Mpro was concentrated (Amicon, 10 kDa) to a concentration of 17 mg/ml (512 µM) Mpro and stored at -70 ºC.

Surface plasmon resonance (SPR) binding analysis

The SPR experiments were performed using a Biacore T200 equipped with a Series S Sensor Chip SA (GE Healthcare #BR-1005-31). Biotinylated COVID-19 Mpro C145A Q306E.AVI (35731 Da, >85% pure, only 21% biotinylated based on MS, DNA sequence available in the SI) was immobilized to the streptavidin covalently attached to a carboxymethyldextran matrix. The initial conditioning of the surfaces on flow cell 1 and 2 was performed by three 1-minute pulses of 1 M NaCl, 50 mM NaOH solution. The ligand at a concentration of 0.27mg/ml in immobilization buffer (10 mM HEPES, 150 mM NaCl, 1mM TCEP, 0.05% P20, pH 7.4) was immobilized at a density of 4300 RU on flow cell 2 at a flow rate of 5 µl/min and flow cell 1 was left blank to serve as a reference surface. Surfaces were stabilized with 3 hours injection at a flow rate of 40 µL/min of running buffer (10 mM HEPES, 150 mM NaCl, 1mM TCEP, 0.05% P20, 5% DMSO, pH 7.4).

To collect kinetic binding data, sample in 10 mM HEPES, 150 mM NaCl, 0.05% P20, 5%DMSO, pH 7.4, was injected over the two flow cells at ascending concentrations (0, 0.27, 0.82, 2.5, 7.5, 22.2, 66.6 and 200 µM) at a flow rate of 40 µl/min and at a temperature of 25°C. The complex was allowed to associate and dissociate for 40 and 100 s, respectively for each sample concentration.

A DMSO correction curve was performed before/after every 104 cycles.

Data were collected at a rate of 10 Hz and fitted to a simple 1:1 interaction model using the global data analysis option available within Biacore T200 Evaluation software.

Crystallization and crystallography

Sitting drop co-crystallization was carried out by adding 0.3 µl each of Mpro (17 mg/ml, buffer as described above) and crystallization solution in 96-well plates (Intelliplate, Art Robbins). The inhibitors were added to the protein approximately one hour before crystallization. Compound 9 and compound 7 were added to Mpro at a final concentration of 1 mM from a 10 mM stock solution in DMSO, while compound 11 was added at a final concentration of 10 mM from a 100 mM stock solution in DMSO.

The inhibitors compound 11 and compound 7 were co-crystallized using 10% (w/v) polyethylene glycol (PEG) 4000, 20% (w/v) glycerol, 0.03 M each of sodium fluoride, sodium bromide and sodium iodide, 0.1 M MES/imidazole pH 6.5 (condition B3 of "Morpheus I" screen, Molecular Dimensions Ltd.). Compound 9 was co-crystallized using 0.1 M MES/imidazole pH 6.5, 10% (w/v) polyethylene glycol 4000, 20% (w/v) glycerol, 0.03 M each of magnesium chloride and calcium chloride (condition A3 of Morpheus I screen, Molecular Dimensions Ltd).

X-ray diffraction experiments were carried out at 100 K at beamline X06SA-PXI of the Swiss Light Source (SLS), Villigen, Switzerland. Measurements were made at the SLS with crystal rotation steps of 0.2 º using an EIGER 16M detector (Dectris).

The data were processed and scaled using AutoProc[41] and XSCALE[42], respectively. Automated molecular replacement

Please do not adjust margins

Please do not adjust margins

was carried out using Dimple[43] (Collaborative Computational Project 1994) with the 3CLpro structure as template. COOT[44] was used for model building. Phenix.refine[45], Buster[45] and Refmac[46] were used for refinement of the structures. Data collection and refinement statistics are reported in Table 3 and Figures of molecular structures were generated with PyMOL(Schrodinger 2015)

This journal is © The Royal Society of Chemistry 20xx

Please do not adjust margins

Please do not adjust margins

Table1. Delivered AI molecules and their biological data

Structure	Compound #	Mpro_FRET_DTT_IC50 [umol/l]	Mpro_Biacore_KD [uM]	CathL_FRET_IC50 [umol/l]	Mpro_MCA_IC50[umol/l]
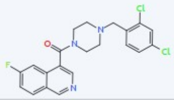	1	3.27		>125	>125
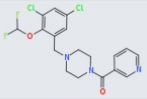	2	63.0		>125	>125
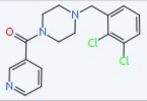	3	63.5		>125	>125
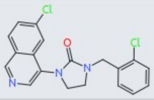	4	NA	185	>125	>125
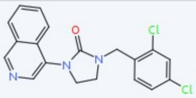	5	NA	368	>125	>125
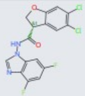	6	22.6	32.8	103	>125
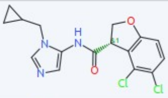	13	>125		>125	>125
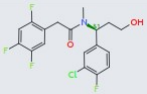	14	>125	>400	>125	
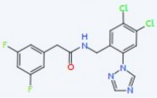	15	>125	>400	>125	
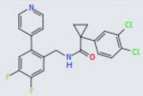	16	>125		>125	>125
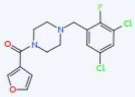	17	>125		>125	>125
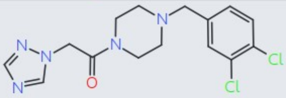	18	>125		>125	>125
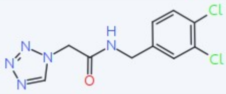	19	>125		>125	>125
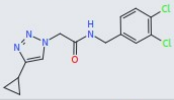	20	>125		>125	>125
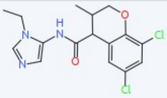	21	66.9		>125	>125
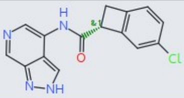	22	>125		>125	>125

Please do not adjust margins

Please do not adjust margins

Table 2. Compounds identified in the frame of the hit expansion and follow-up optimized compounds

Structure	Compound #	Mpro_FRET_DTT_IC50 [umol/l]	Mpro_Biacore_KD [uM]	CathL_FRET_IC50 [umol/l]	Mpro_MCA_IC50 [umol/l]
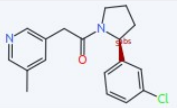	7	2.10		>125	>125
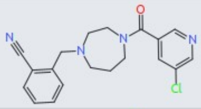	8	24.228 (mean, n=4)	49.8	>124.8 (mean, n=5)	>96.625 (mean, n=4)
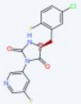	9	26.2	37.8	>125	>125
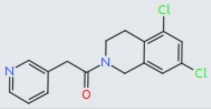	10	62.6	324	>125	>125
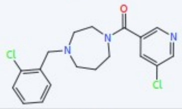	11	1.26		>125	
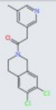	12	2.31 (mean, n=2)		>125 (mean, n=2)	>125 (mean, n=2)

This journal is © The Royal Society of Chemistry 20xx

Please do not adjust margins

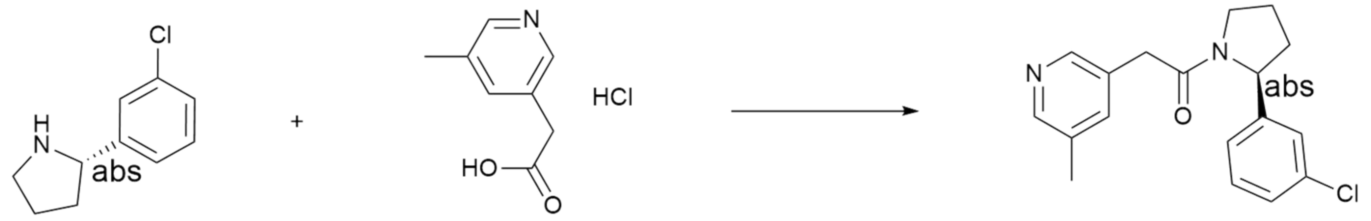

Scheme 1 Synthetic route to compound 7. Reagents and conditions: HATU, DIPEA, DMF, RT

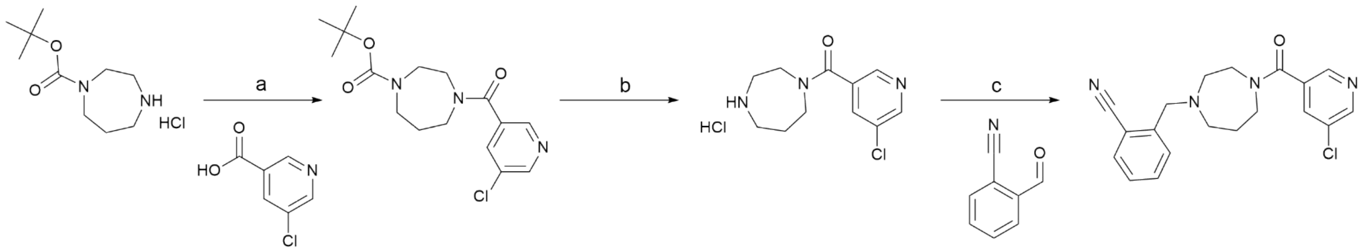

Scheme 2 Synthetic route to compound 8. Reagents and conditions: (a) HATU, DIPEA, DMF, RT; (b) HCl, MeOH, RT; (c) $NaBH(OAc)_3$, DIPEA, DCM, RT

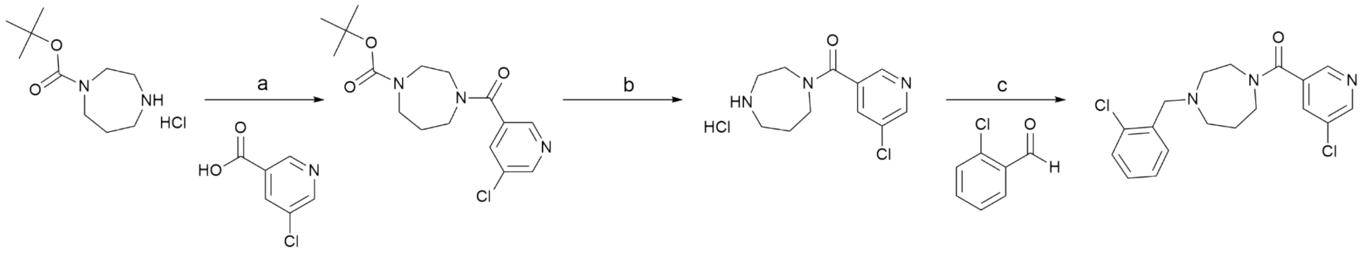

Scheme 3 Synthetic route to compound 11. Reagents and conditions: (a) HATU, DIPEA, DMF, RT; (b) HCl, MeOH, RT; (c) $NaBH(OAc)_3$, DIPEA, DCM, RT

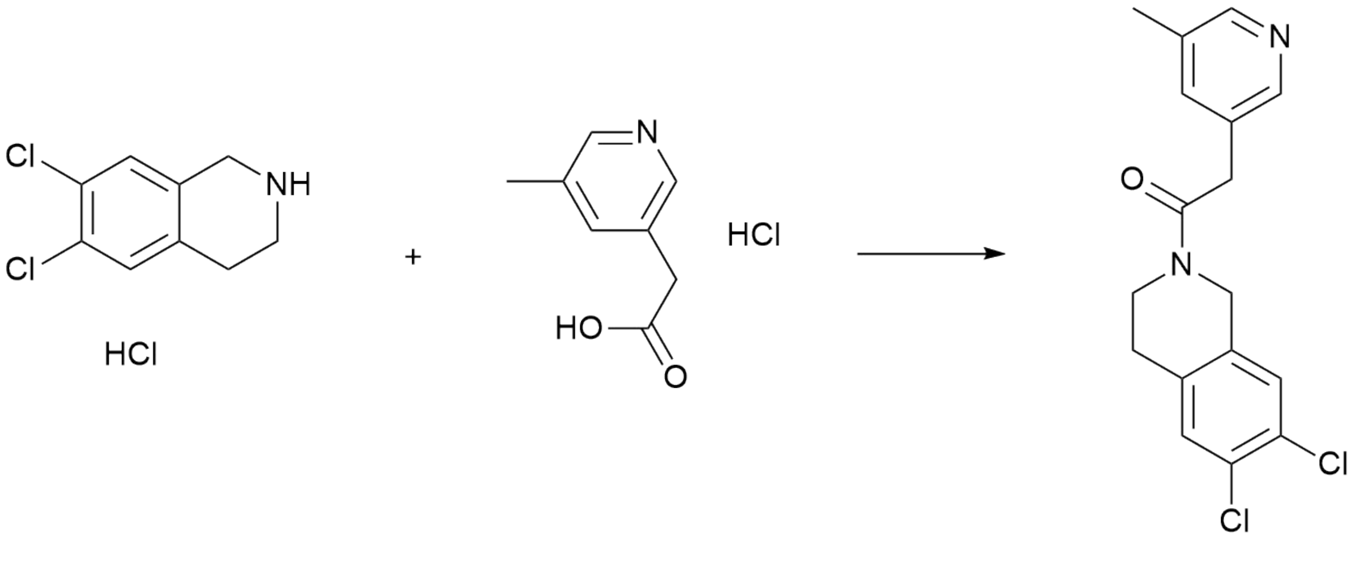

Scheme 4 Synthetic route to compound 12. Reagents and conditions: HATU, DIPEA, DMF, RT

Please do not adjust margins

Table 3. X-ray data collection and processing for compounds 7, 8, 11 and 12

Compound	Cpd 7	Cpd 8	Cpd 11	Cpd 12
Diffraction source	Swiss Light Source, X06DA (PXIII)	Swiss Light Source, X06SA (PXI)	Swiss Light Source, X06DA (PXII)	Swiss Light Source, X06DA (PXIII)
Wavelength (Å)	1.00	1.00	1.00	1.00
Temperature (K)	100	100	100	100
Detector	Eiger 16M	Eiger 16M	Eiger 16M	Eiger 16M
Crystal to detector distance (mm)	135	135	150	135
Total rotation range (°)	180	360	180	360
Rotation per image (°)	0.25	0.20	0.25	0.20
Exposure time per image (s)	0.120	0.015	0.250	0.125
Space group	P 21 21 21	P 21 21 21	P 21 21 21	P 21 21 21
a, *b*, *c* (Å)	68.1 102.0 104.7	68.0 100.3 104.6	67.8 101.2 103.8	68.0 101.8 105.0
α β, γ (°)	90 90 90	90 90 90	90 90 90	90 90 90
Mosaicity (°)	0.19	0.16	0.12	0.08
Resolution range (Å) (highest shell)	51.0-1.31 (1.40-1-31)	56.76-1.59 (1.76-1.59)	72.47-1.34 (1.42-1.34)	49.80-1.30 (1.33-1.30)
Total no. of reflections	777335 (34443)	538998 (26606)	897063 (36301)	1190708 (84732)
No. of unique reflections	142995 (7149)	81101 (4055)	136333 (6816)	179022 (13124)
Completeness (%) (spherical)	81.8 (21.9)	73.2 (15.9)	84.3 (25.4)	99.8 (99.7)
Completeness (%) (ellipsoidal)	93.7 (52.1)	94.8 (61.4)	96.1 (66.4)	Not calculated
Multiplicity	5.4 (4.8)	6.6 (6.6)	6.6 (5.3)	3.4 (3.3)
$\langle I/\sigma(I)\rangle$ from merged data	7.6 (1.6)	13.9 (1.8)	15.4 (1.7)	10.3 (1.1)
$CC_{1/2}$ (highest shell)	(50.2)	(54.4)	(69.0)	(36.5)

This journal is © The Royal Society of Chemistry 20xx

Please do not adjust margins

Please do not adjust margins

HTS QC Analysis Report

Sample Name: LCMS384-20221005-1_E11

Compound 1

Instrument: ICHALW-DL00012-TQD

Acquisition time: 10/6/2022 12:12 AM

Expression	Result
Auto-Comments	
Auto-Summary	GOOD
Purity	96.4000
Target Mass	418.3000
Target RT	0.8750

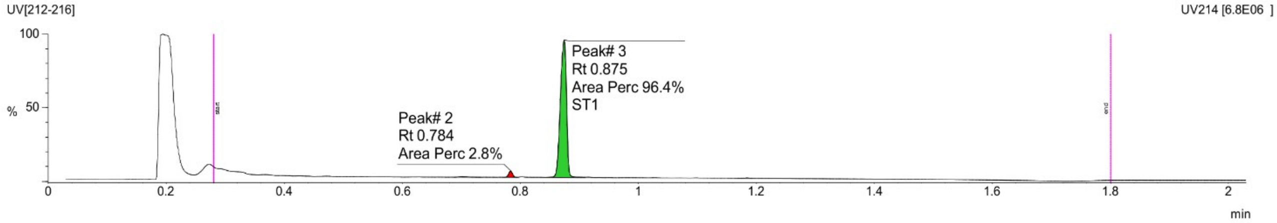

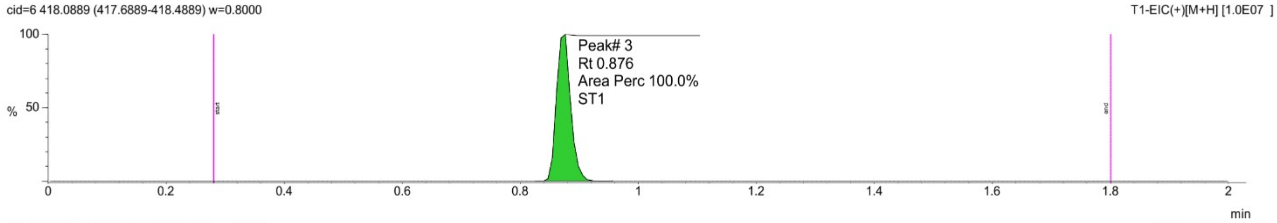

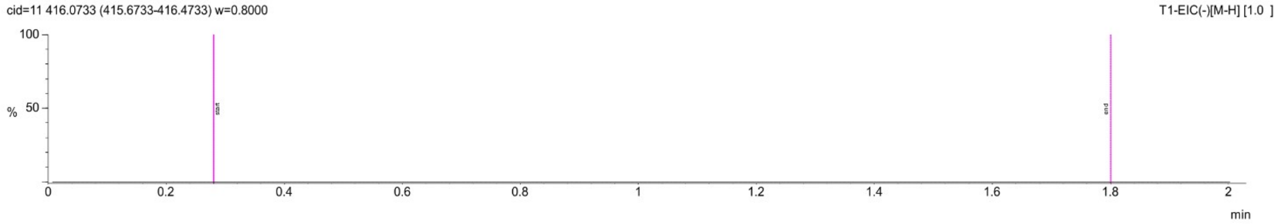

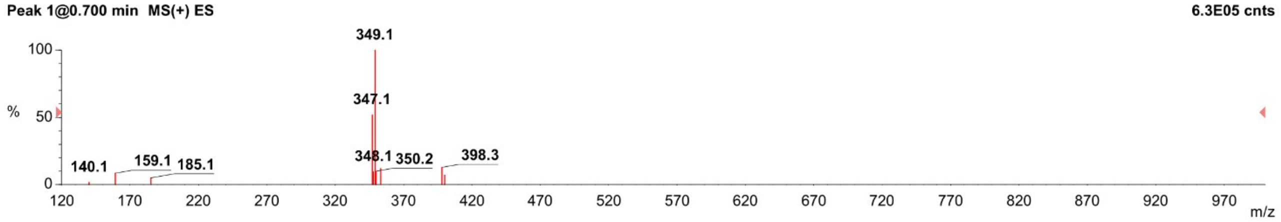

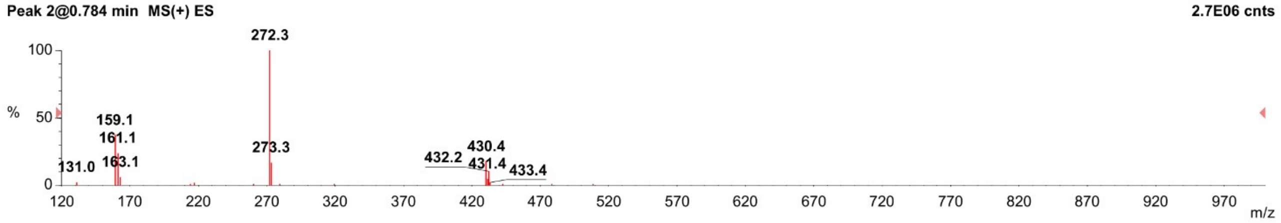

Printed: 10/6/2022 1:49 PM

Please do not adjust margins

Page 2 of 6

HTS QC Analysis Report

Sample Name: LCMS384-20221005-1_E11

Compound 1

Instrument: ICHALW-DL00012-TQD **Acquisition time:** 10/6/2022 12:12 AM

Peak 3@0.876 min MS(+) ES **1.4E07 cnts**

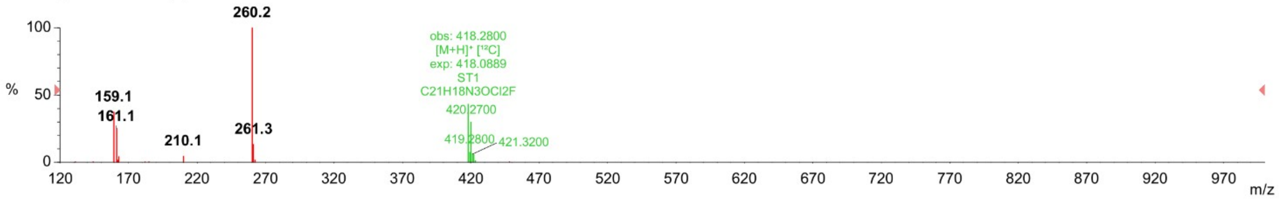

Peak 4@1.185 min MS(+) ES **2.7E05 cnts**

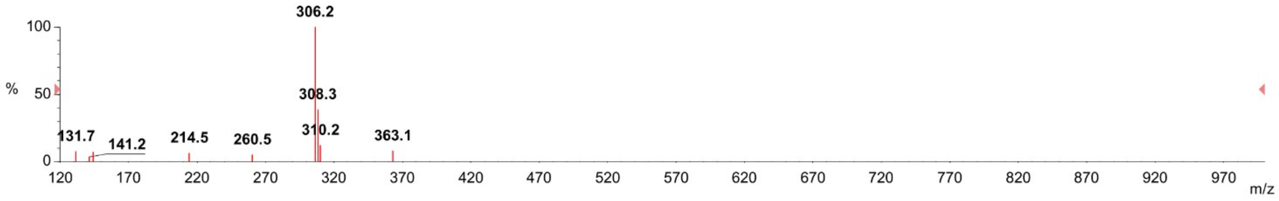

Printed: 10/6/2022 1:49 PM

This journal is © The Royal Society of Chemistry 20xx

Please do not adjust margins

idorsia HTS QC Analysis Report

Sample Name: LCMS384-20221005-1_C13

Compound 2

Instrument: ICHALW-DL00012-TQD

Acquisition time: 10/6/2022 12:32 AM

Expression	Result
Auto-Comments	
Auto-Summary	GOOD
Purity	97.0000
Target Mass	416.3000
Target RT	0.9160

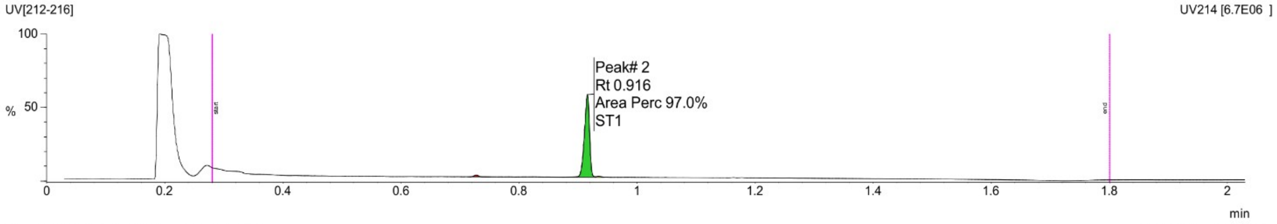

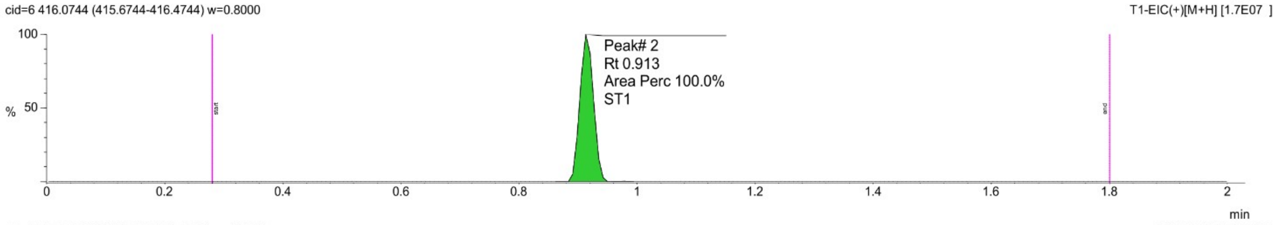

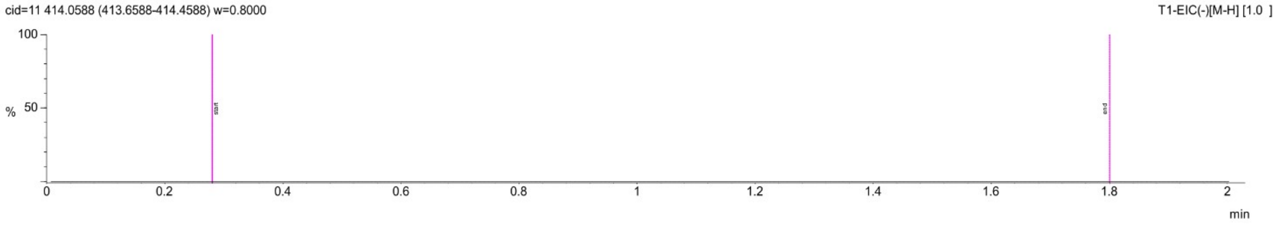

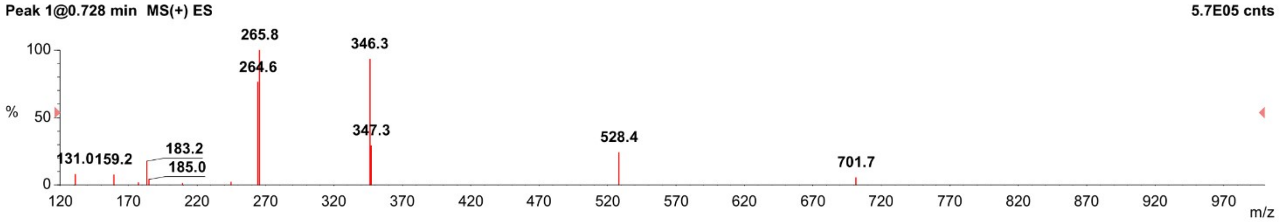

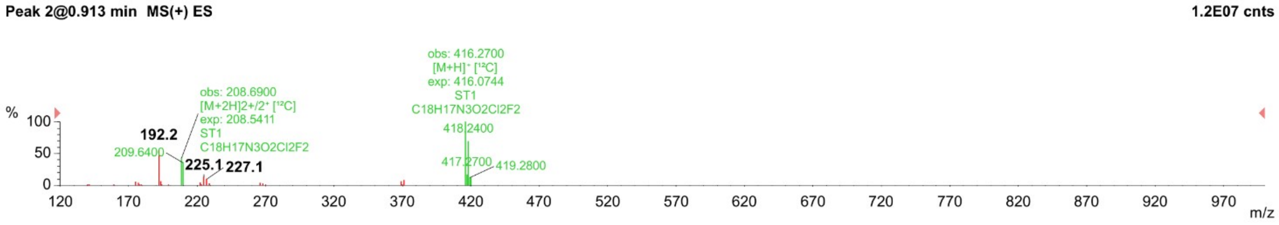

Printed: 10/6/2022 1:49 PM

Please do not adjust margins

Please do not adjust margins

Page 1 of 5

HTS QC Analysis Report

Sample Name: LCMS384-20221005-1_M11

Compound 3

Instrument: ICHALW-DL00012-TQD

Acquisition time: 10/6/2022 12:23 AM

**Expression**	**Result**
Auto-Comments	
Auto-Summary	GOOD
Purity	99.4000
Target Mass	350.2000
Target RT	0.6310

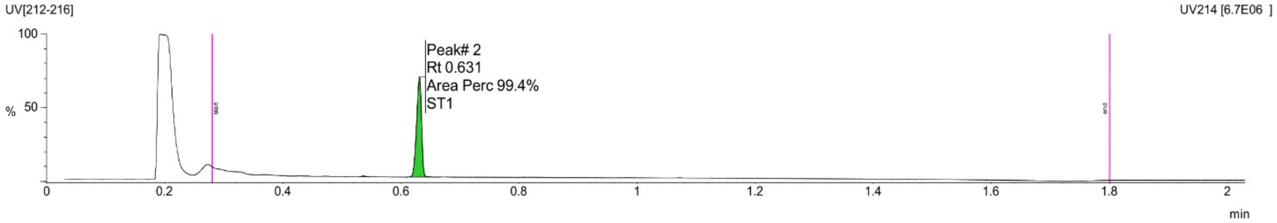

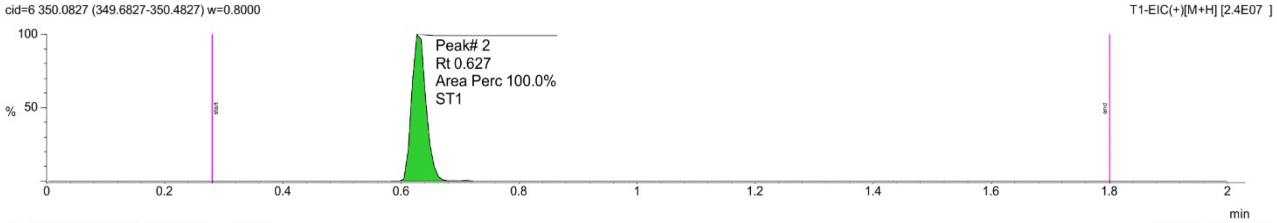

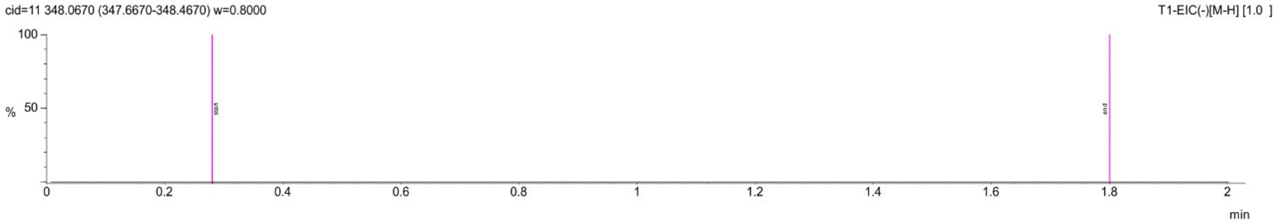

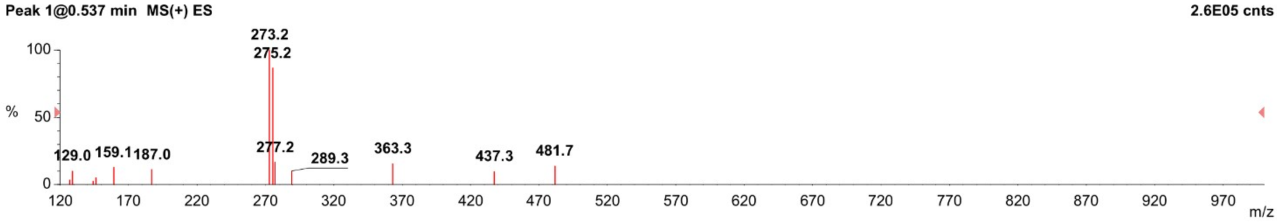

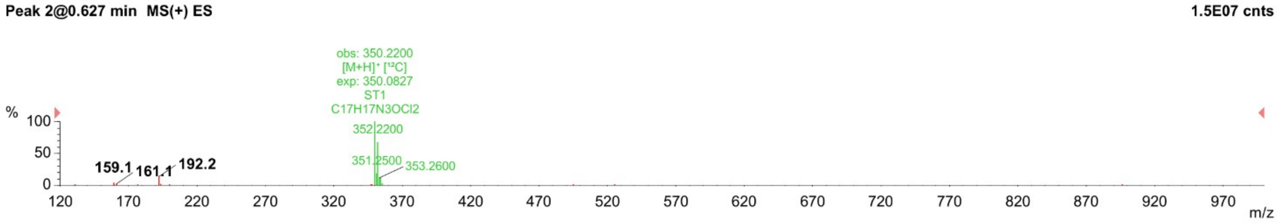

Printed: 10/6/2022 1:48 PM

This journal is © The Royal Society of Chemistry 20xx

Please do not adjust margins

idorsia HTS QC Analysis Report

Sample Name: LCMS384-20221005-1_E13

Compound 4

Instrument: ICHALW-DL00012-TQD

Acquisition time: 10/6/2022 12:35 AM

Expression	Result
Auto-Comments	
Auto-Summary	GOOD
Purity	89.9000
Target Mass	372.3000
Target RT	1.1020

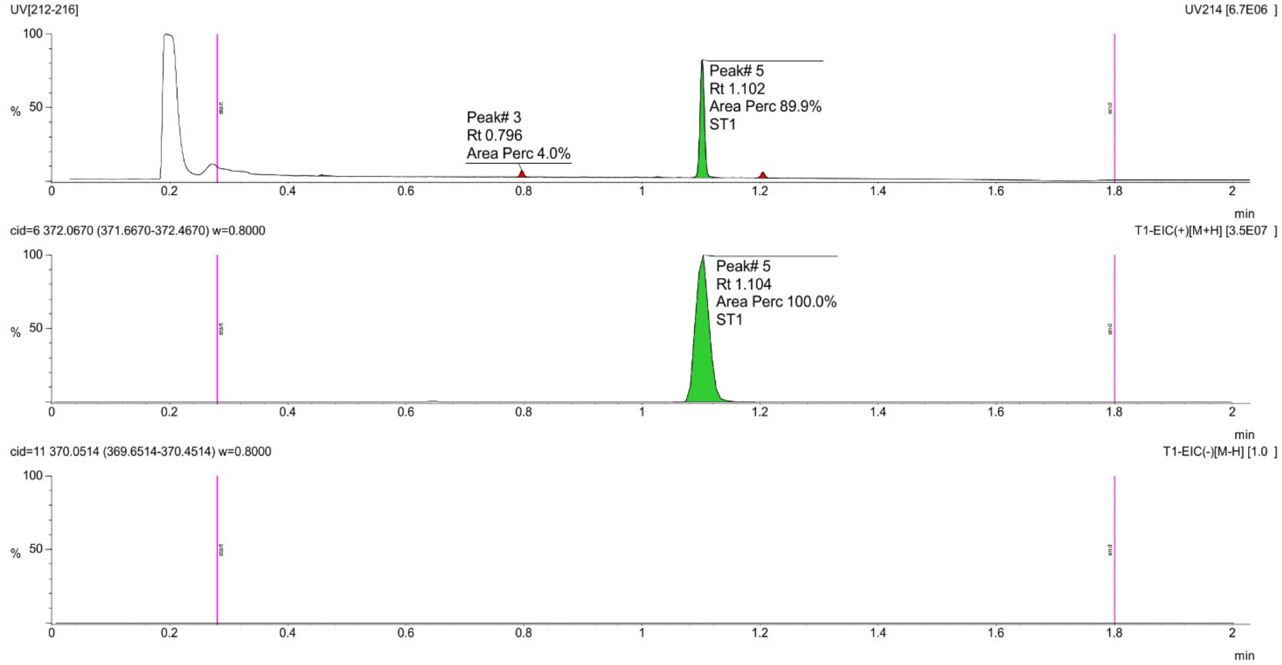

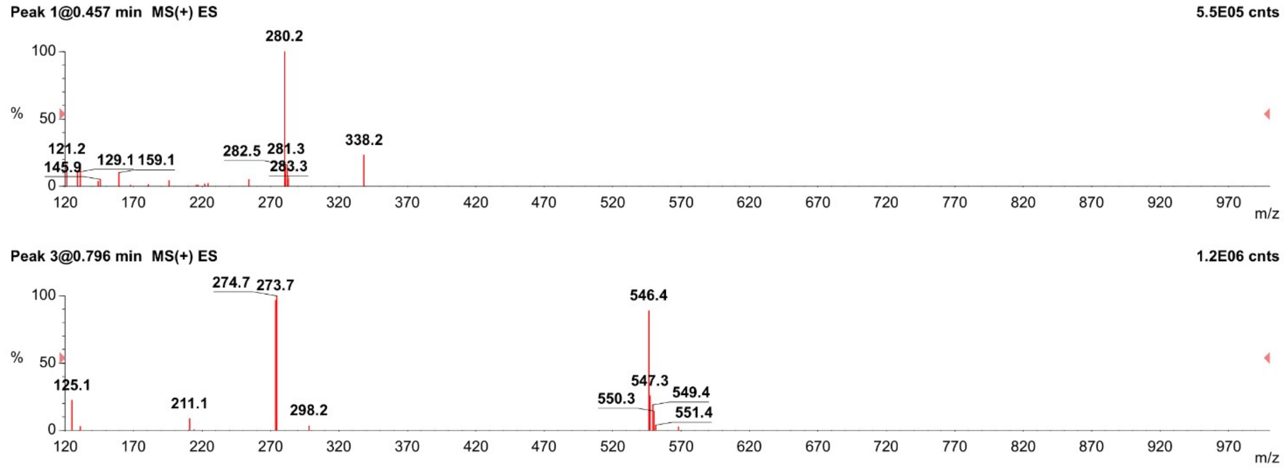

Printed: 10/6/2022 1:48 PM

HTS QC Analysis Report

Sample Name: LCMS384-20221005-1_E13

Compound 4

Instrument: ICHALW-DL00012-TQD

Acquisition time: 10/6/2022 12:35 AM

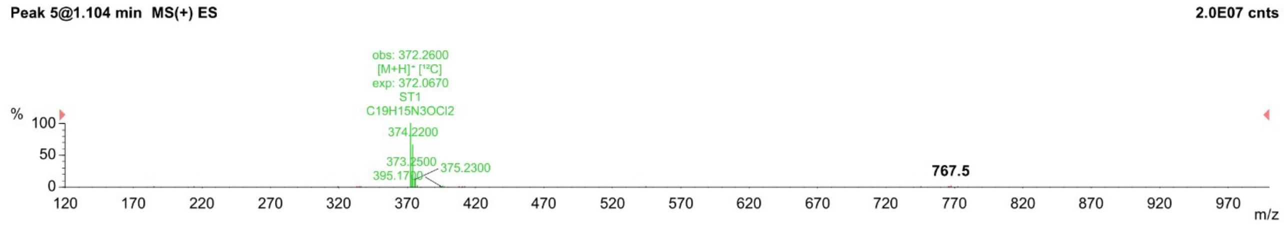

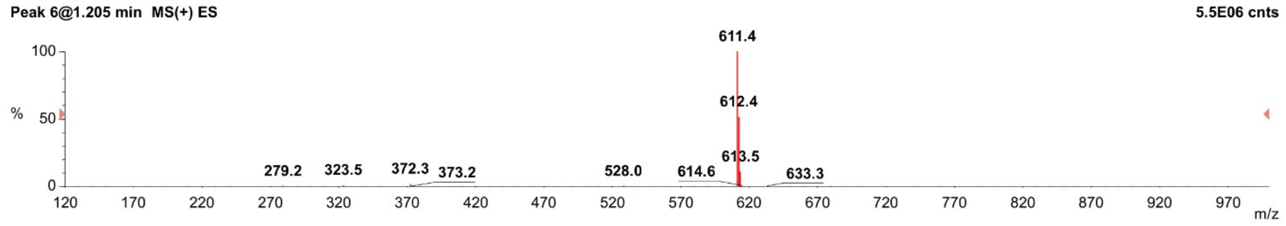

Printed: 10/6/2022 1:48 PM

This journal is © The Royal Society of Chemistry 20xx

Please do not adjust margins

Please do not adjust margins

Page 1 of 6

idorsia **HTS QC Analysis Report**

Sample Name: LCMS384-20221005-1_I11

Compound 5

Instrument: ICHALW-DL00012-TQD

Acquisition time: 10/6/2022 12:17 AM

Expression	Result
Auto-Comments	
Auto-Summary	GOOD
Purity	97.2000
Target Mass	372.2000
Target RT	1.0370

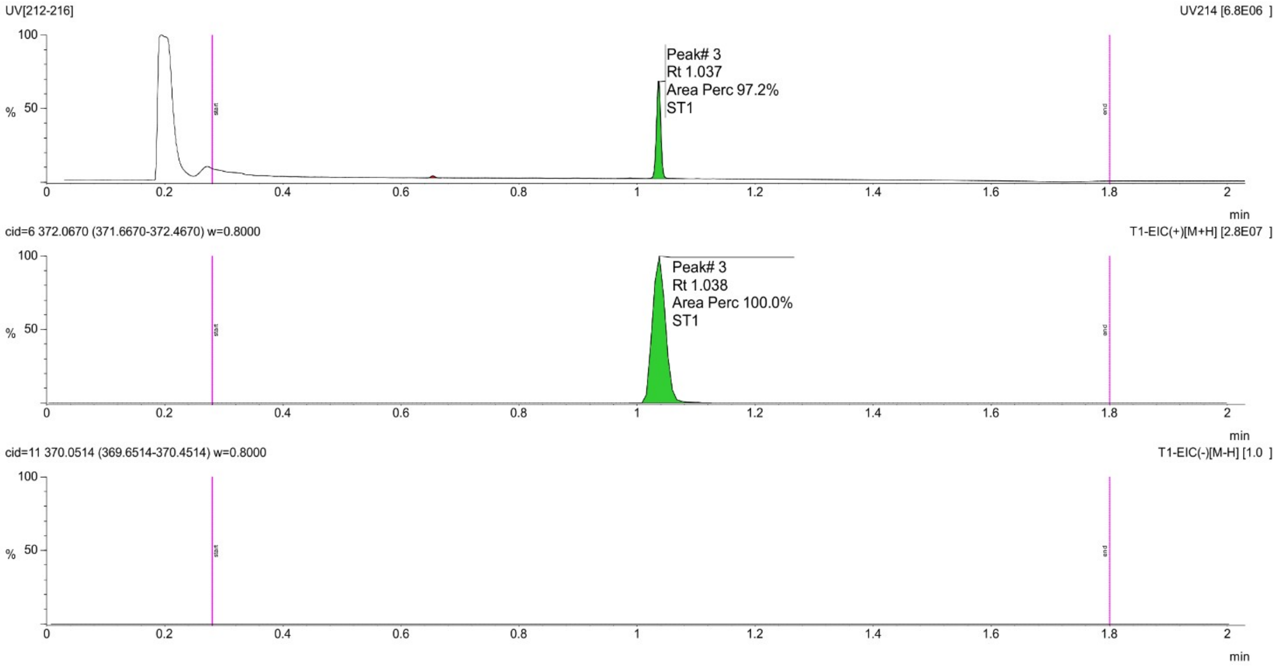

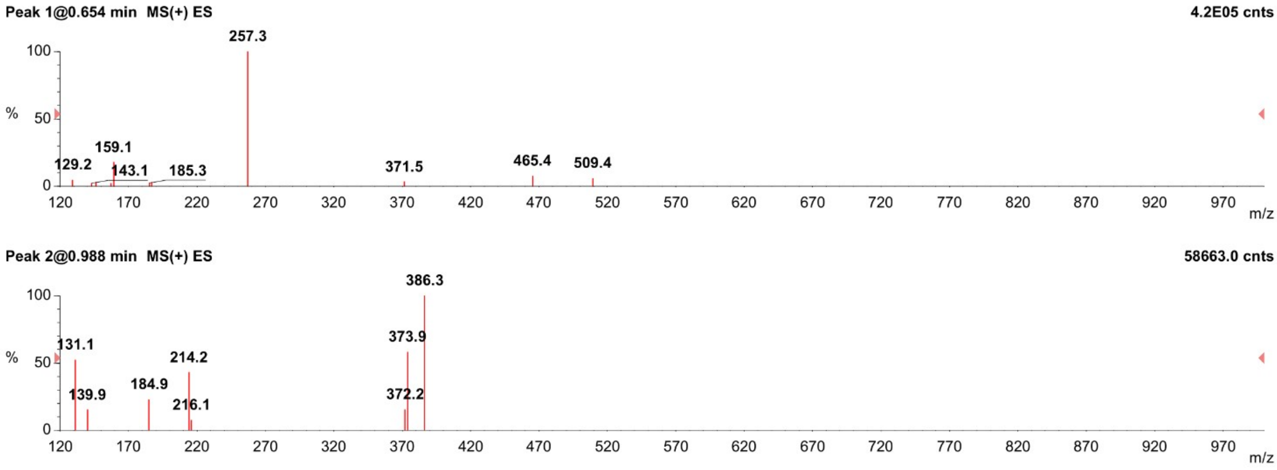

Printed: 10/6/2022 1:50 PM

This journal is © The Royal Society of Chemistry 20xx

Please do not adjust margins

Page 2 of 6

HTS QC Analysis Report

Sample Name: LCMS384-20221005-1_I11

Compound 5

Instrument: ICHALW-DL00012-TQD

Acquisition time: 10/6/2022 12:17 AM

Peak 3@1.038 min MS(+) ES **1.9E07 cnts**

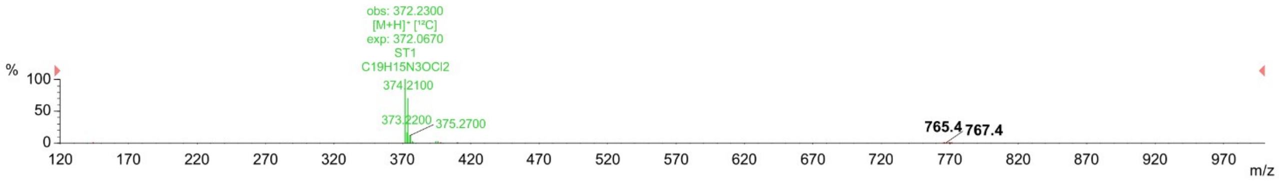

Printed: 10/6/2022 1:50 PM

This journal is © The Royal Society of Chemistry 20xx

Please do not adjust margins

idorsia HTS QC Analysis Report

Sample Name: LCMS384-20221005-1_I11

Compound 6

Instrument: ICHALW-DL00012-TQD

Acquisition time: 10/6/2022 12:17 AM

Expression	Result
Auto-Comments	
Auto-Summary	GOOD
Purity	97.2000
Target Mass	372.2000
Target RT	1.0370

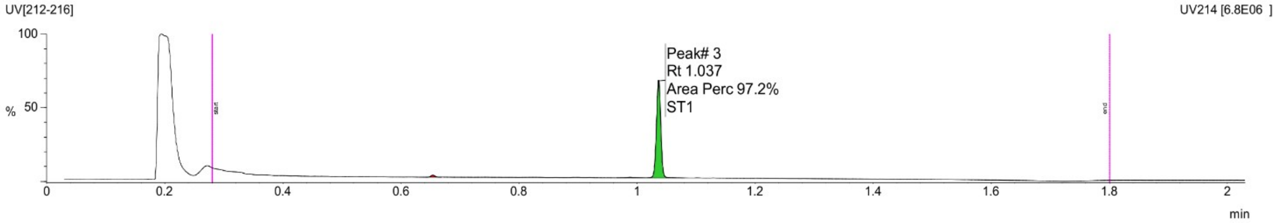

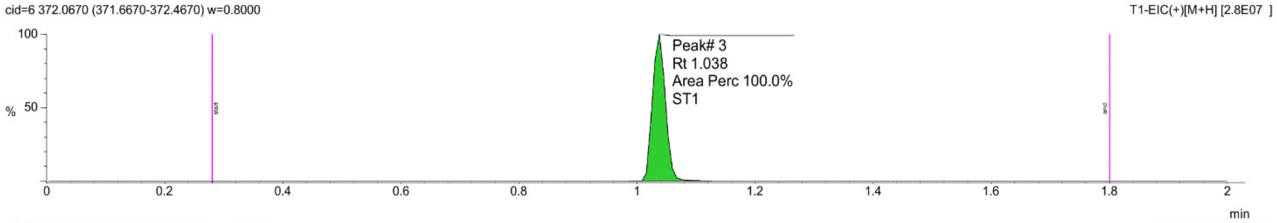

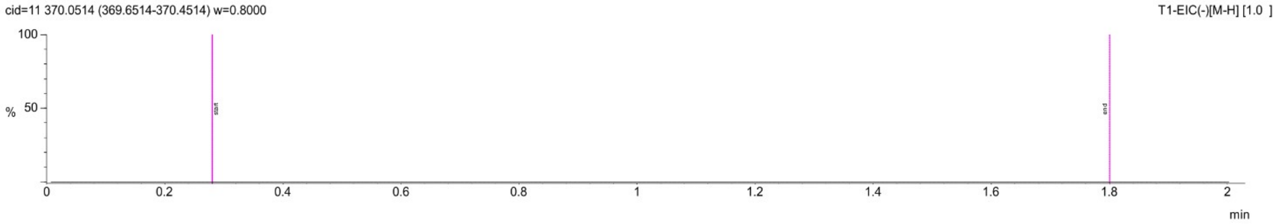

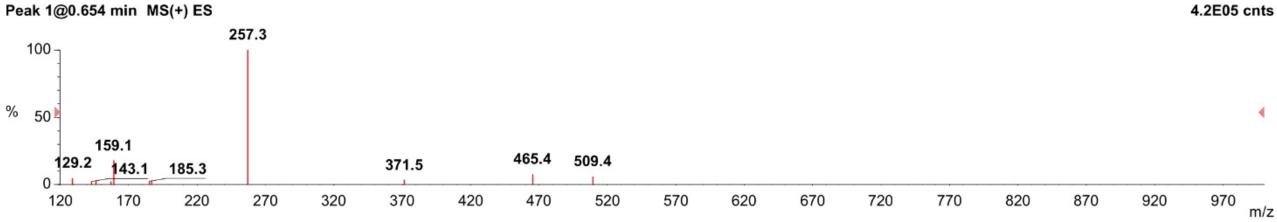

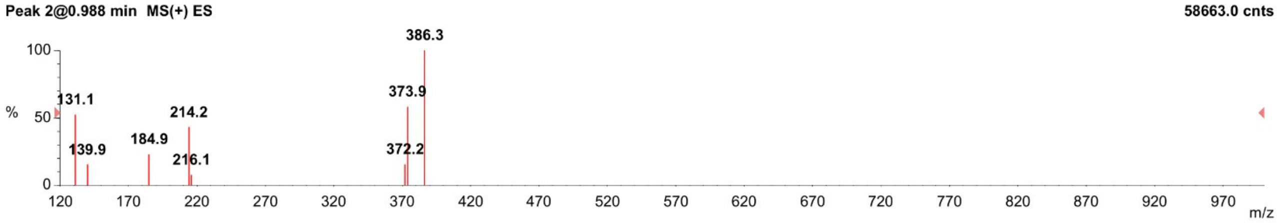

Printed: 10/6/2022 1:50 PM

Please do not adjust margins

idorsia **HTS QC Analysis Report**

Sample Name: LCMS384-20221005-1_I11

Compound 6

Instrument: ICHALW-DL00012-TQD

Acquisition time: 10/6/2022 12:17 AM

Peak 3@1.038 min MS(+) ES **1.9E07 cnts**

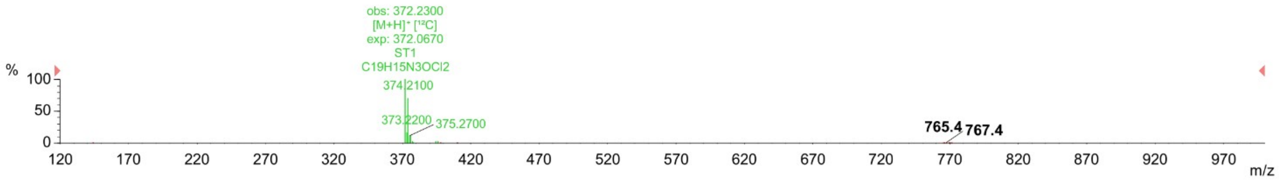

Printed: 10/6/2022 1:50 PM

This journal is © The Royal Society of Chemistry 20xx

Please do not adjust margins

HTS QC Analysis Report

Sample Name: LCMS384-20220927-4_M01

Compound 7

Instrument: ICHALW-DL00021-SQD

Acquisition time: 9/27/2022 1:46 PM

Expression	Result
Auto-Comments	
Auto-Summary	GOOD
Purity	100.0000
Target Mass	315.4000
Target RT	0.6330

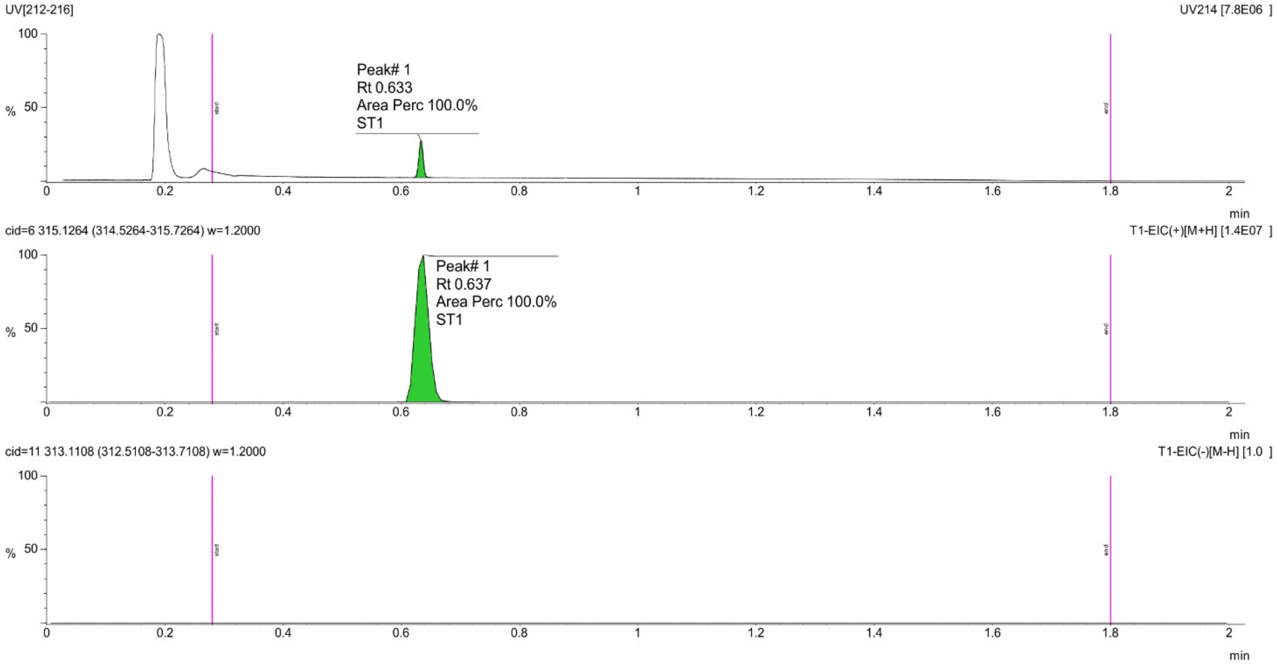

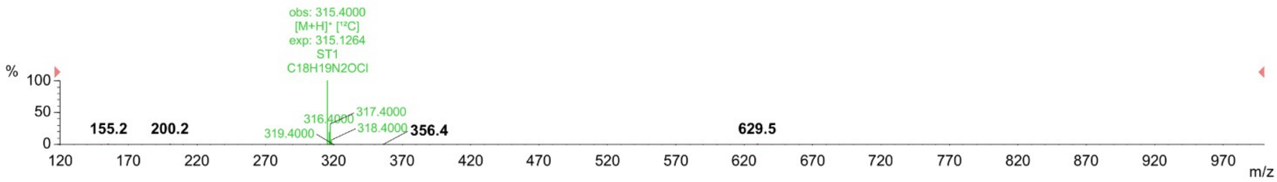

Printed: 9/28/2022 8:44 AM

Page 1 of 5

idorsia HTS QC Analysis Report

Sample Name: LCMS384-20220518-2_C13

Compound 8

Instrument: ICHALW-DL00021-SQD

Acquisition time: 5/18/2022 4:55 PM

Expression	Result
Auto-Comments	
Auto-Summary	GOOD
Purity	97.7000
Target Mass	355.4000
Target RT	0.5270

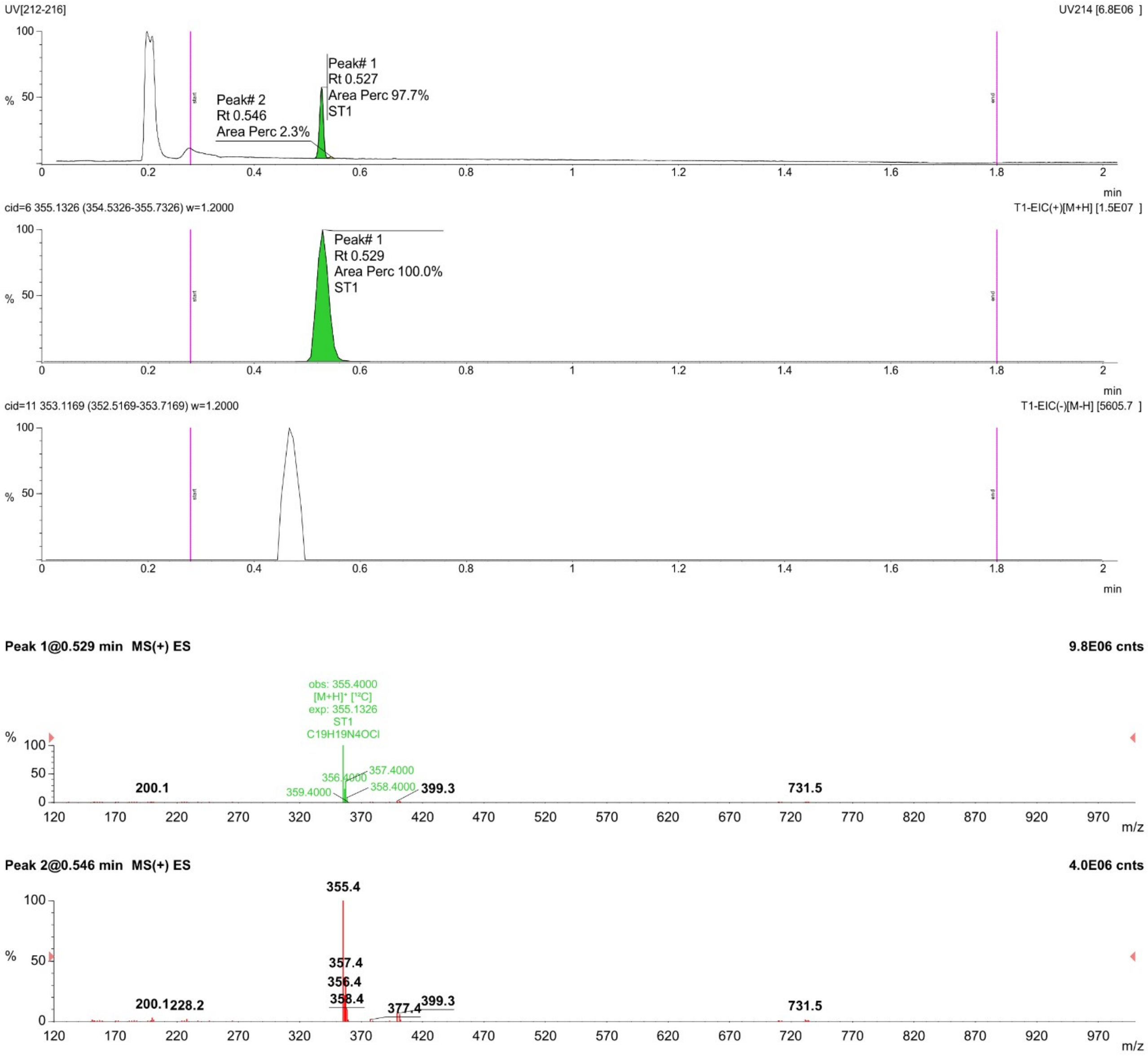

Printed: 5/19/2022 5:07 PM

This journal is © The Royal Society of Chemistry 20xx

Please do not adjust margins

Please do not adjust margins

Page 1 of 5

HTS QC Analysis Report

Sample Name: LCMS384-20220718-2_G09

Compound 9

Instrument: ICHALW-DL00021-SQD

Acquisition time: 7/18/2022 2:04 PM

Expression	Result
Auto-Comments	
Auto-Summary	GOOD
Purity	98.2000
Target Mass	338.4000
Target RT	0.9260

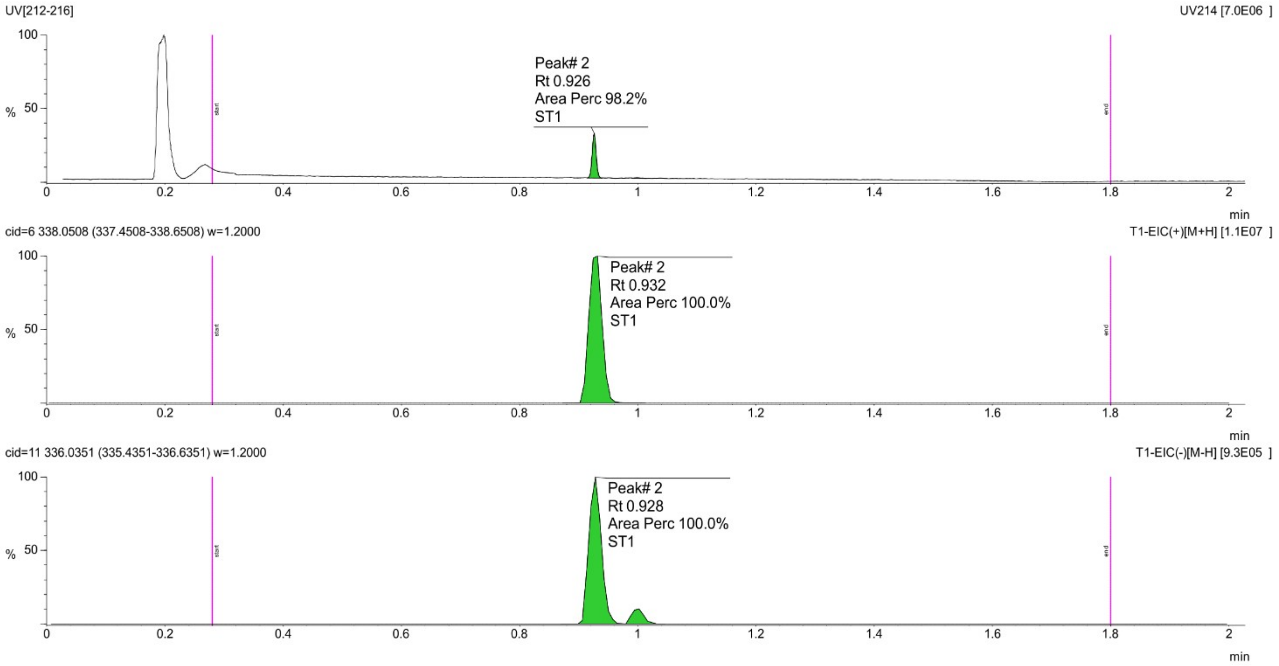

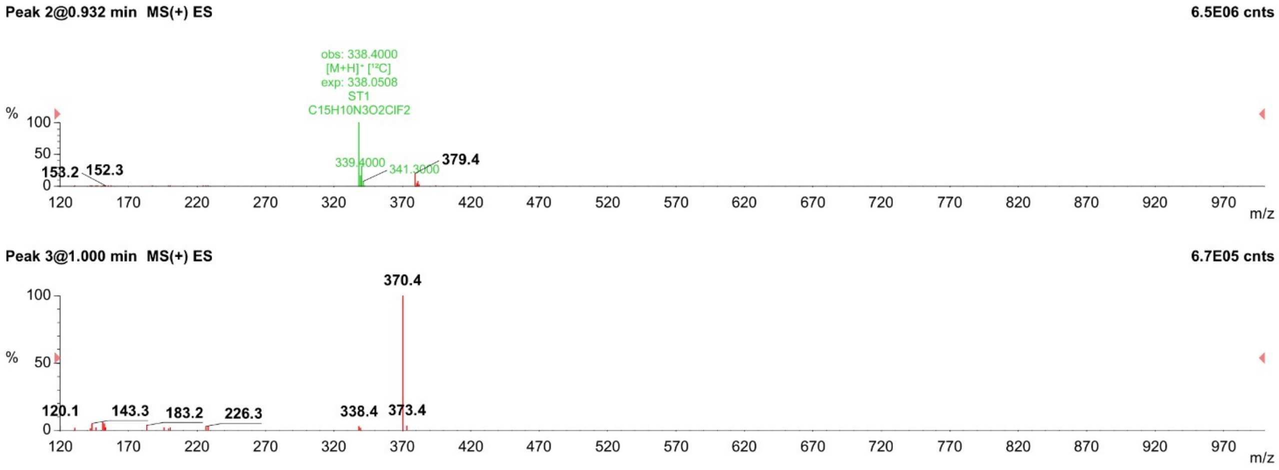

Printed: 7/18/2022 4:39 PM

idorsia HTS QC Analysis Report

Sample Name: LCMS384-20220718-2_E09

Compound 10

Instrument: ICHALW-DL00021-SQD

Acquisition time: 7/18/2022 2:01 PM

**Expression**	**Result**
Auto-Comments	
Auto-Summary	GOOD
Purity	95.1000
Target Mass	321.3000
Target RT	0.7460

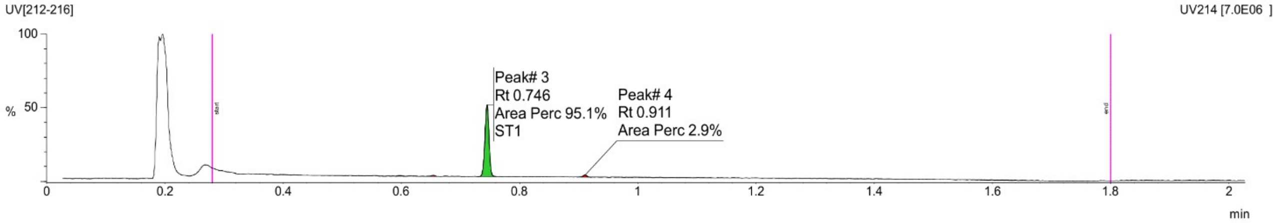

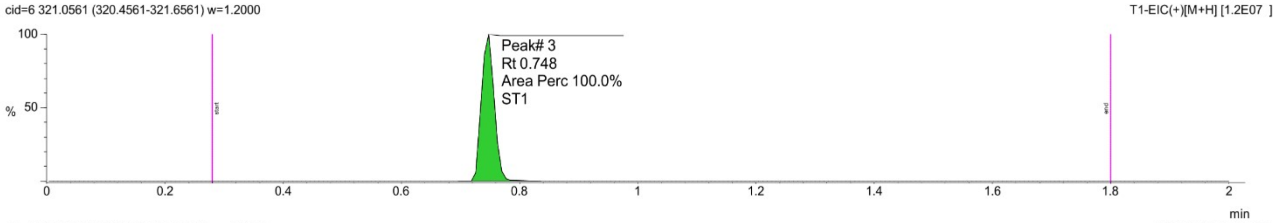

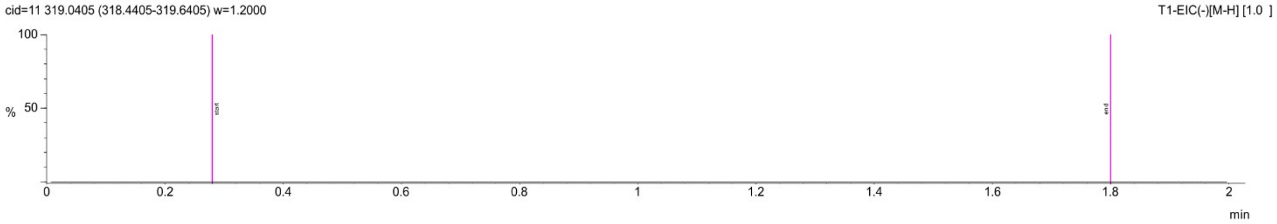

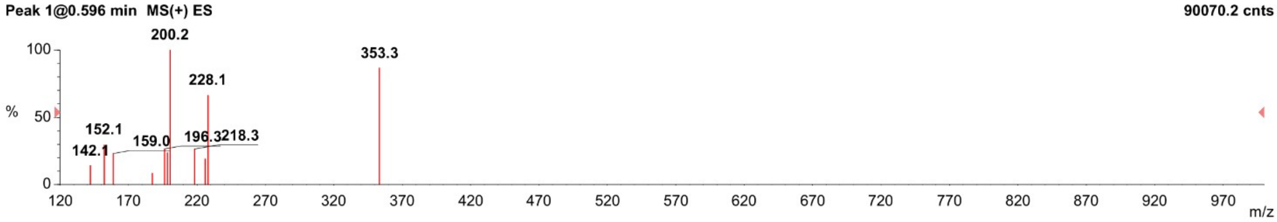

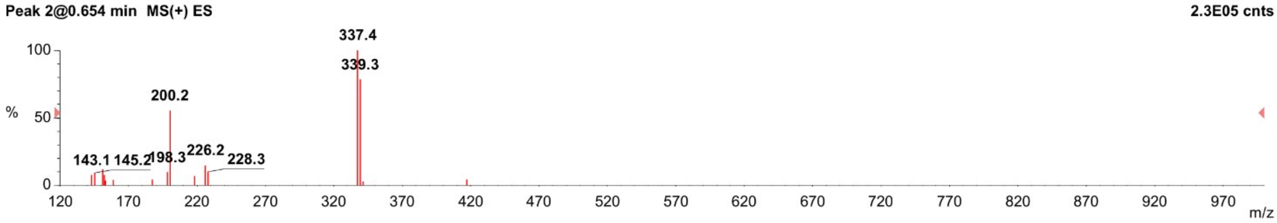

Printed: 7/18/2022 4:38 PM

This journal is © The Royal Society of Chemistry 20xx

Please do not adjust margins

idorsia HTS QC Analysis Report

Sample Name: LCMS384-20220718-2_E09

Compound 10

Instrument: ICHALW-DL00021-SQD

Acquisition time: 7/18/2022 2:01 PM

Peak 3@0.748 min MS(+) ES **7.7E06 cnts**

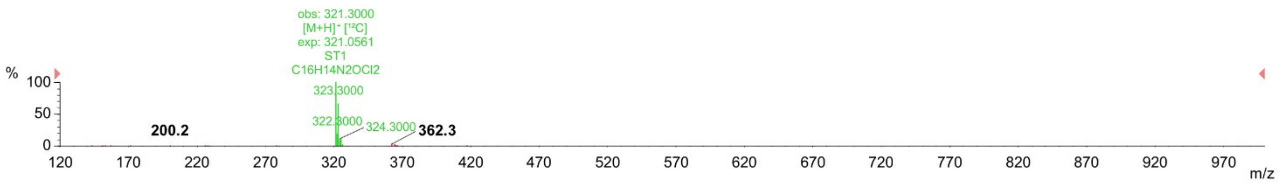

Peak 4@0.911 min MS(+) ES **93590.7 cnts**

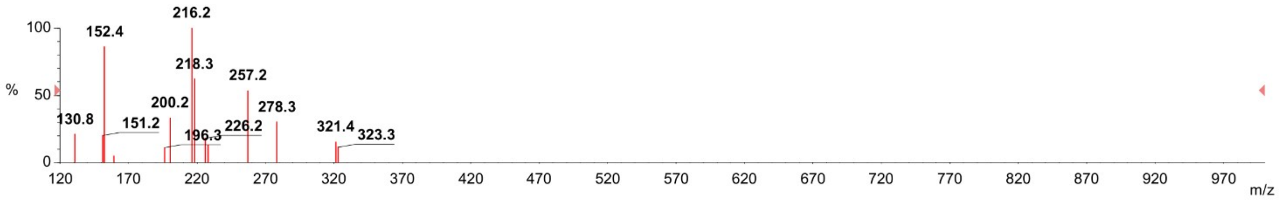

Printed: 7/18/2022 4:38 PM

idorsia HTS QC Analysis Report

Sample Name: LCMS384-20221011-4_M05

Compound 11

Instrument: ICHALW-DL00012-TQD

Acquisition time: 10/11/2022 3:46 PM

Expression	Result
Auto-Comments	
Auto-Summary	GOOD
Purity	97.7000
Target Mass	364.3000
Target RT	0.5160

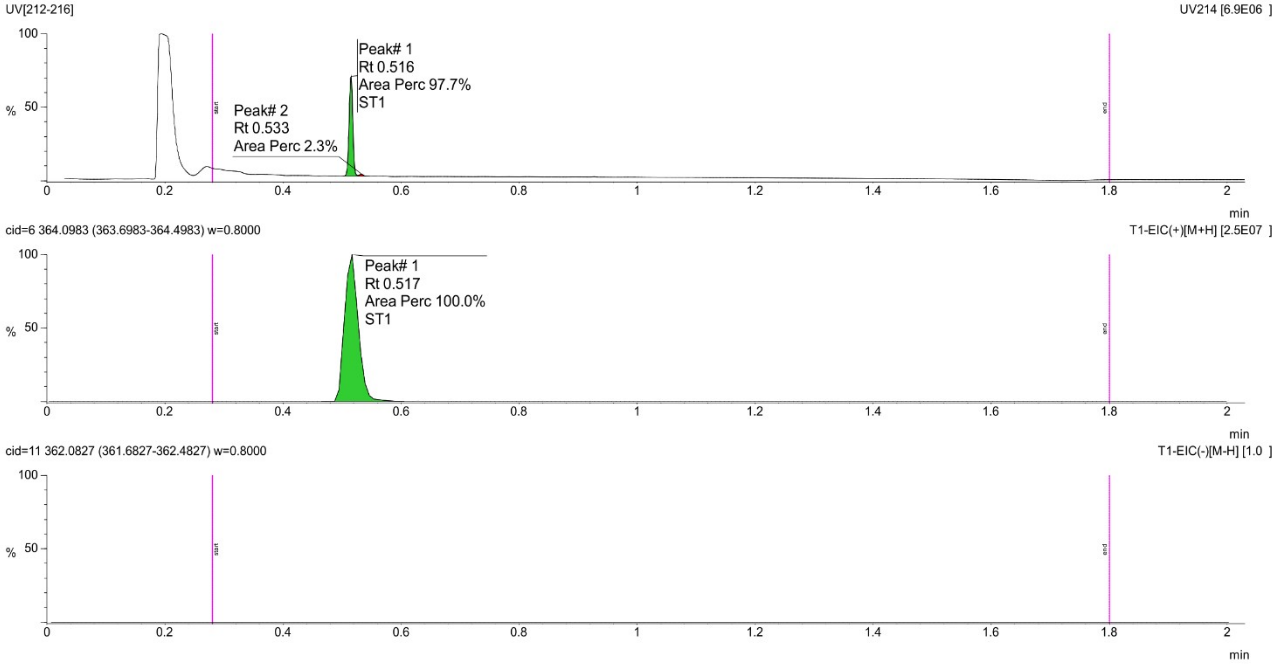

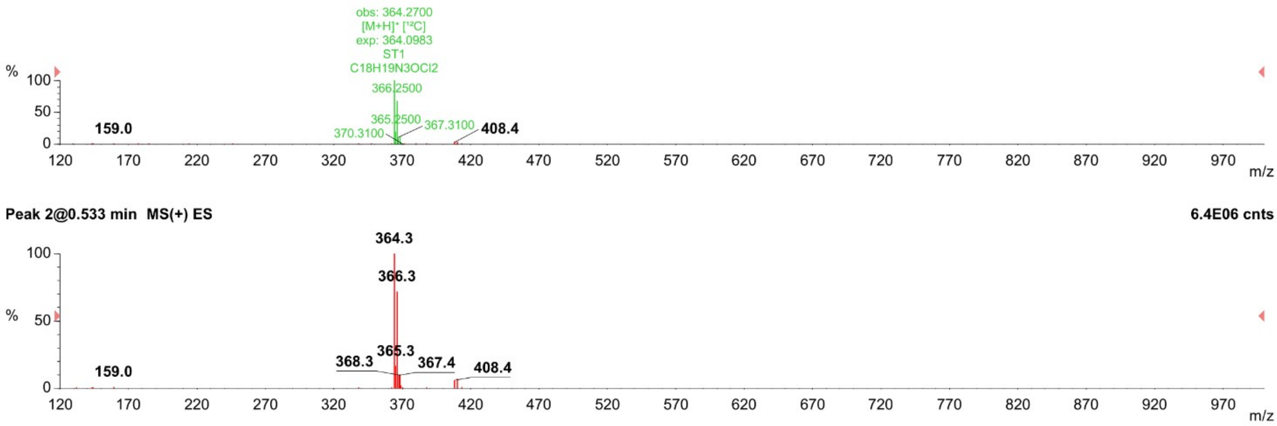

Printed: 10/12/2022 9:24 AM

This journal is © The Royal Society of Chemistry 20xx

Please do not adjust margins

idorsia HTS QC Analysis Report

Sample Name: LCMS384-20220822-2_I05

Compound 12

Instrument: ICHALW-DL00012-TQD

Acquisition time: 8/22/2022 2:12 PM

Expression	Result
Auto-Comments	
Auto-Summary	GOOD
Purity	91.6000
Target Mass	335.2000
Target RT	0.7380

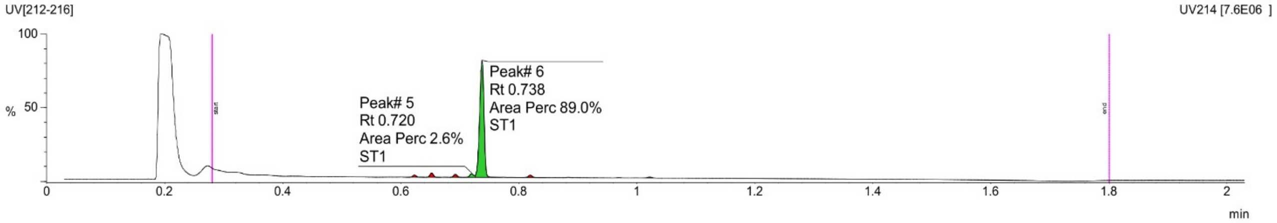

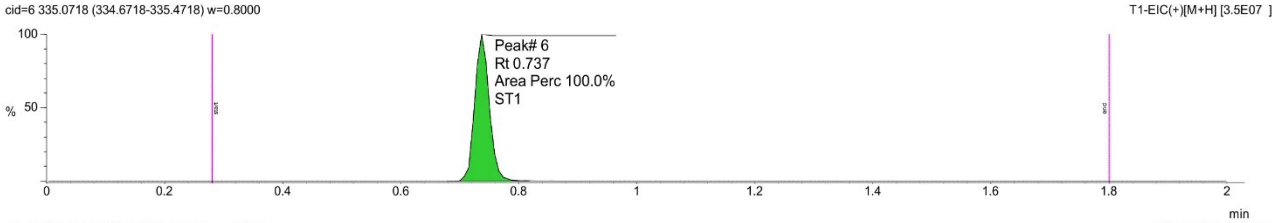

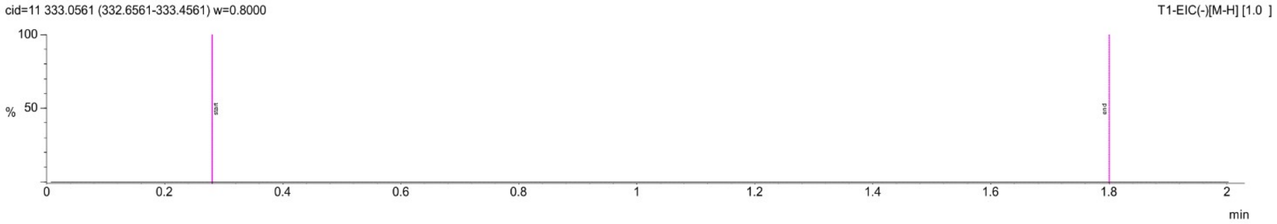

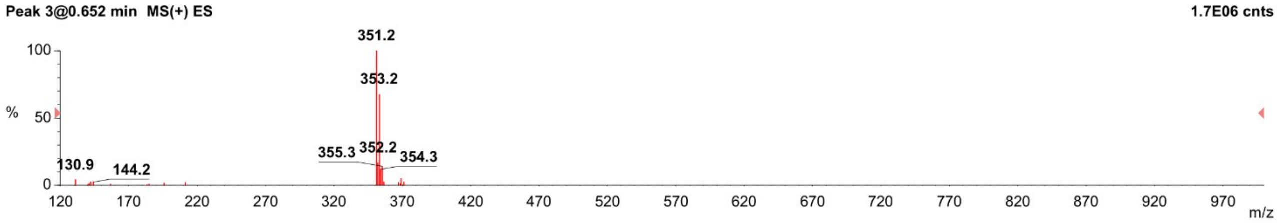

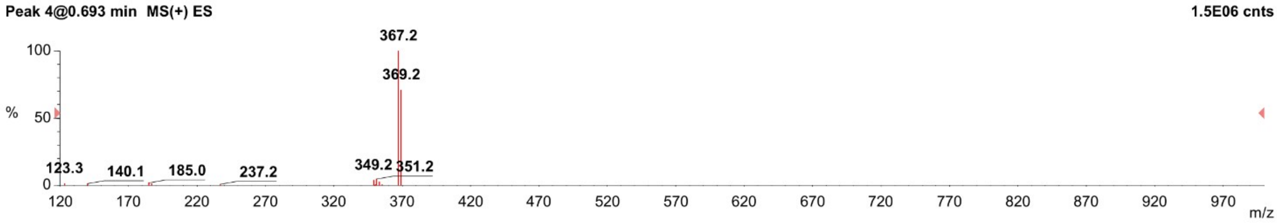

Printed: 8/23/2022 11:49 AM

Please do not adjust margins

Page 2 of 6

HTS QC Analysis Report

Sample Name: LCMS384-20220822-2_I05

Compound 12

Instrument: ICHALW-DL00012-TQD

Acquisition time: 8/22/2022 2:12 PM

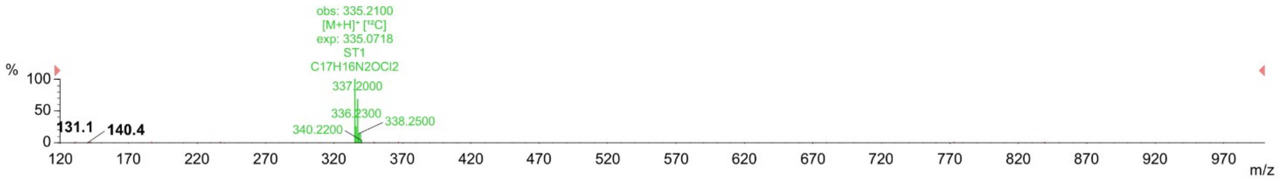

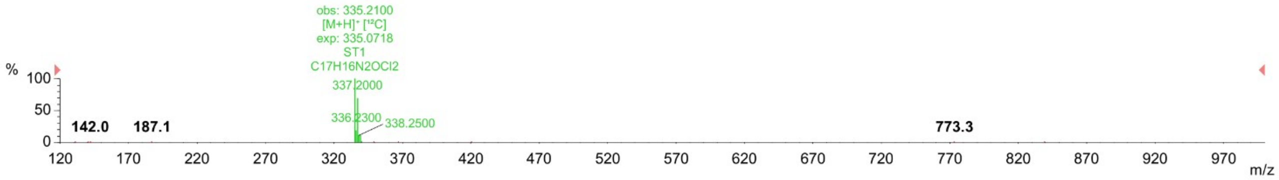

Printed: 8/23/2022 11:49 AM

This journal is © The Royal Society of Chemistry 20xx

HTS QC Analysis Report

Sample Name: LCMS384-20230119-3_G03

Compound 13

Instrument: ICHALW-DL00012-TQD

Acquisition time: 1/20/2023 9:38 AM

Expression	Result
Auto-Comments	
Auto-Summary	GOOD
Purity	97.6000
Target Mass	352.2000
Target RT	0.6160

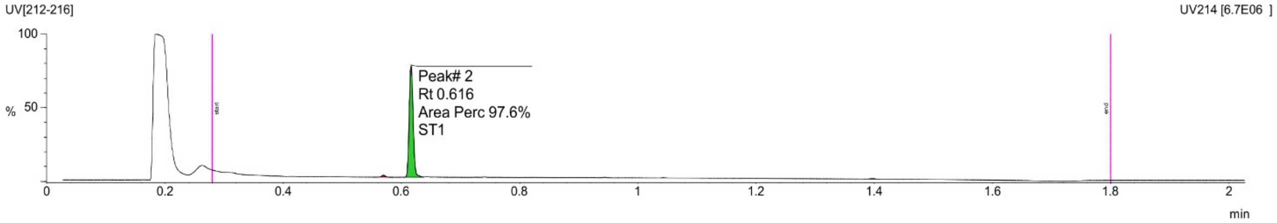

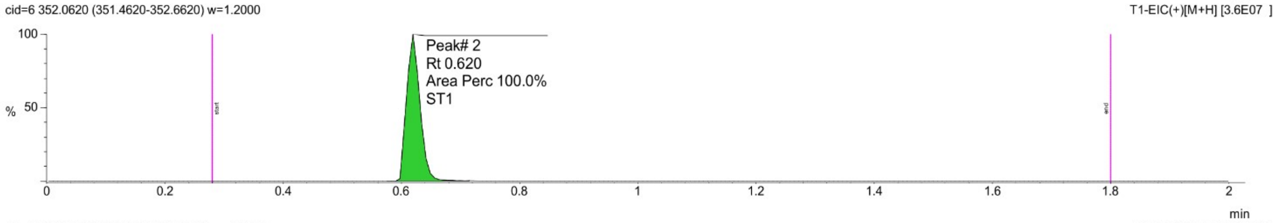

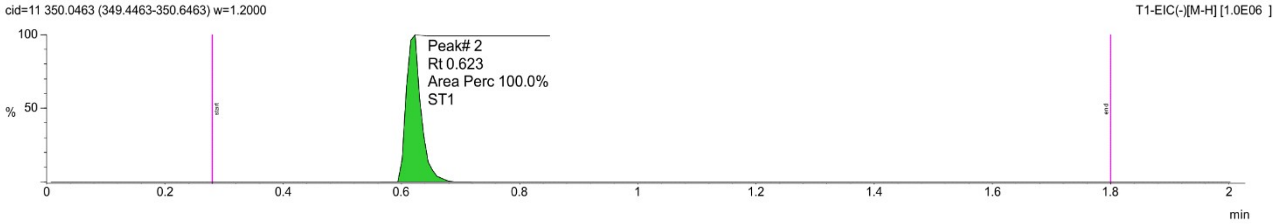

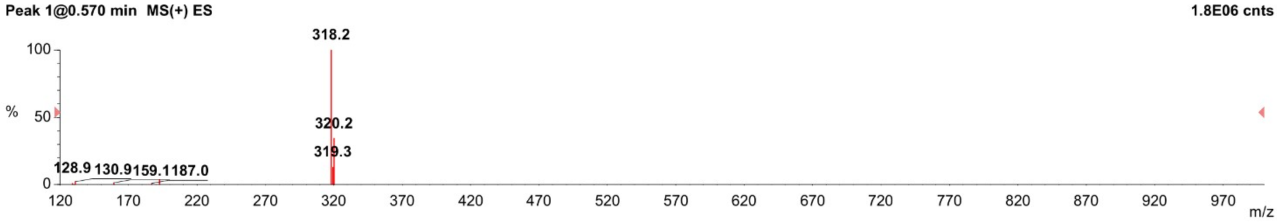

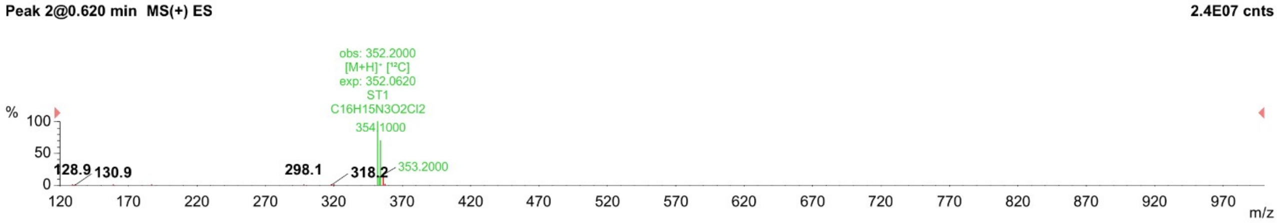

Printed: 1/20/2023 2:50 PM

idorsia HTS QC Analysis Report

Sample Name: LCMS384-20220530-2_G03

Compound 14

Instrument: ICHALW-DL00012-TQD

Acquisition time: 5/30/2022 7:58 PM

Expression	Result
Auto-Comments	
Auto-Summary	GOOD
Purity	99.2000
Target Mass	390.2000
Target RT	1.1040

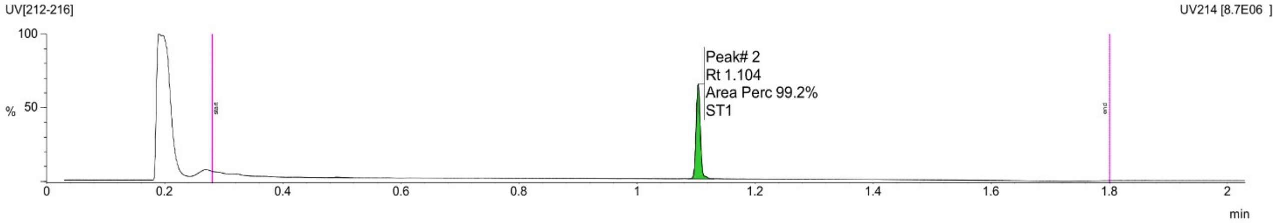

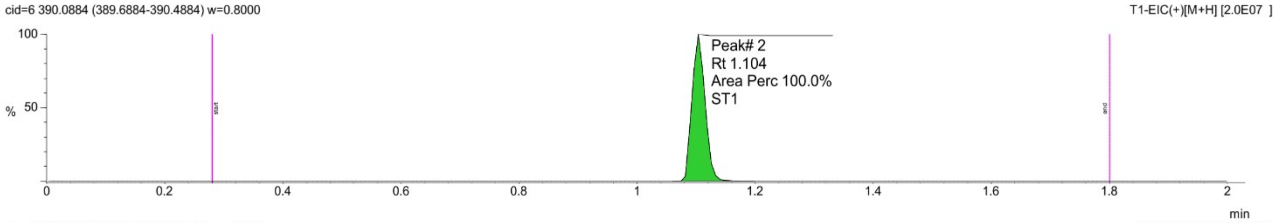

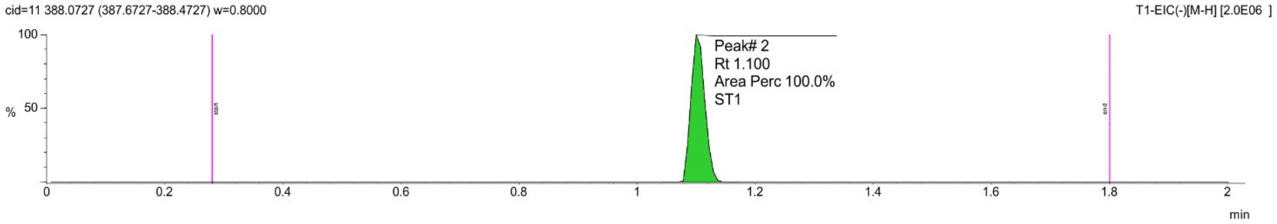

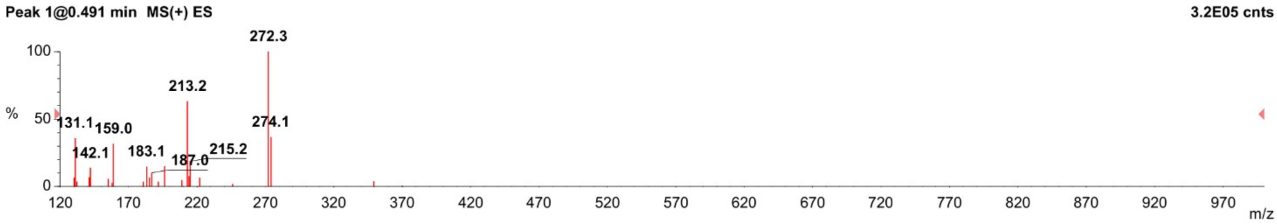

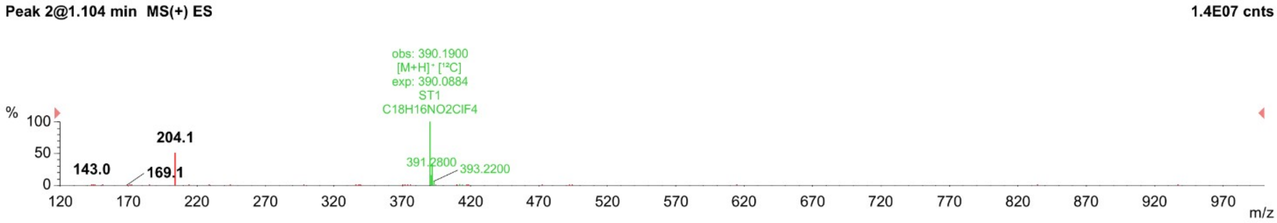

Printed: 5/31/2022 9:41 AM

 This journal is © The Royal Society of Chemistry 20xx

idorsia **HTS QC Analysis Report**

Sample Name: LCMS384-20220530-2_K01

Compound 15

Instrument: ICHALW-DL00012-TQD

Acquisition time: 5/30/2022 7:41 PM

**Expression**	**Result**
Auto-Comments	
Auto-Summary	GOOD
Purity	100.0000
Target Mass	397.1000
Target RT	1.0430

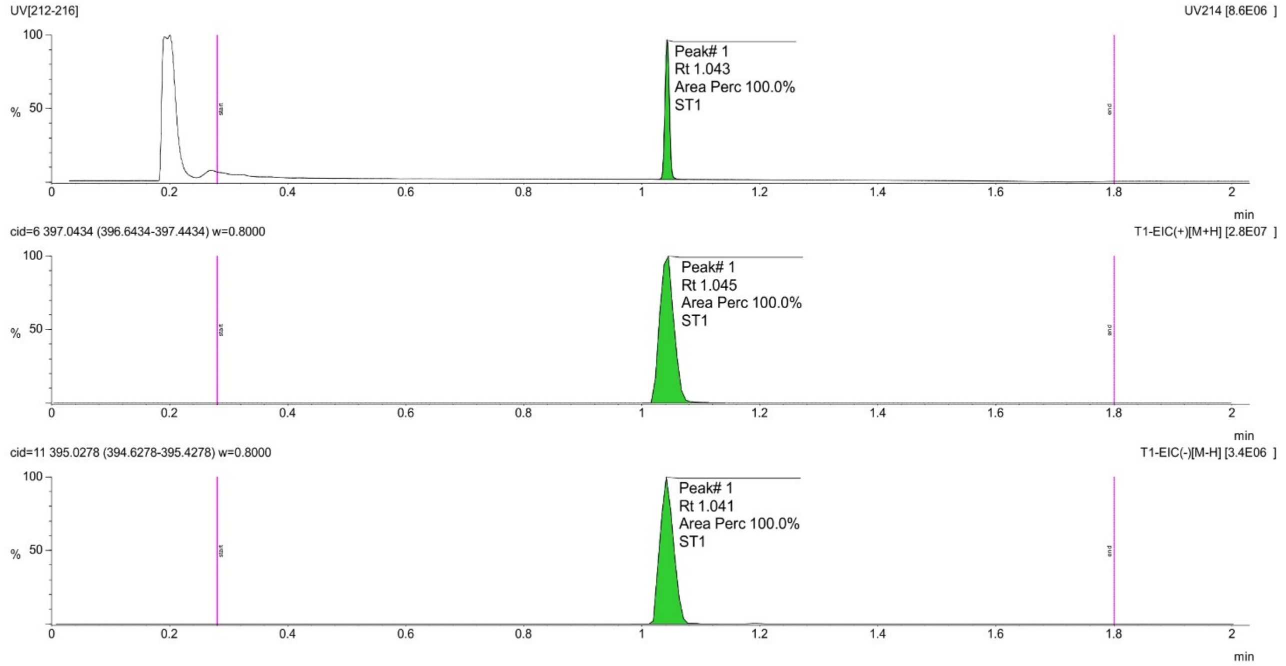

Peak 1@1.045 min MS(+) ES **2.1E07 cnts**

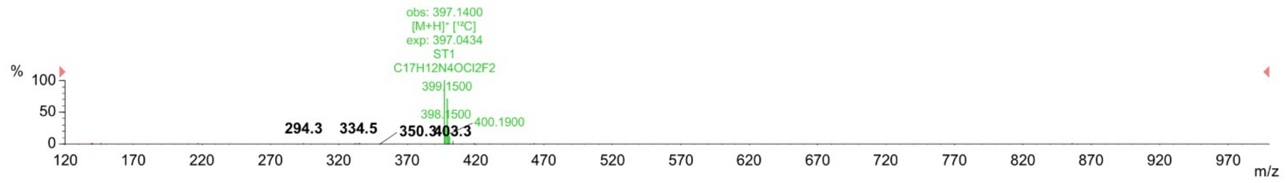

Printed: 5/31/2022 9:41 AM

Please do not adjust margins

HTS QC Analysis Report

Sample Name: LCMS384-20221005-1_G11

Compound 16

Instrument: ICHALW-DL00012-TQD

Acquisition time: 10/6/2022 12:14 AM

Expression	Result
Auto-Comments	
Auto-Summary	GOOD
Purity	94.9000
Target Mass	433.3000
Target RT	1.1260

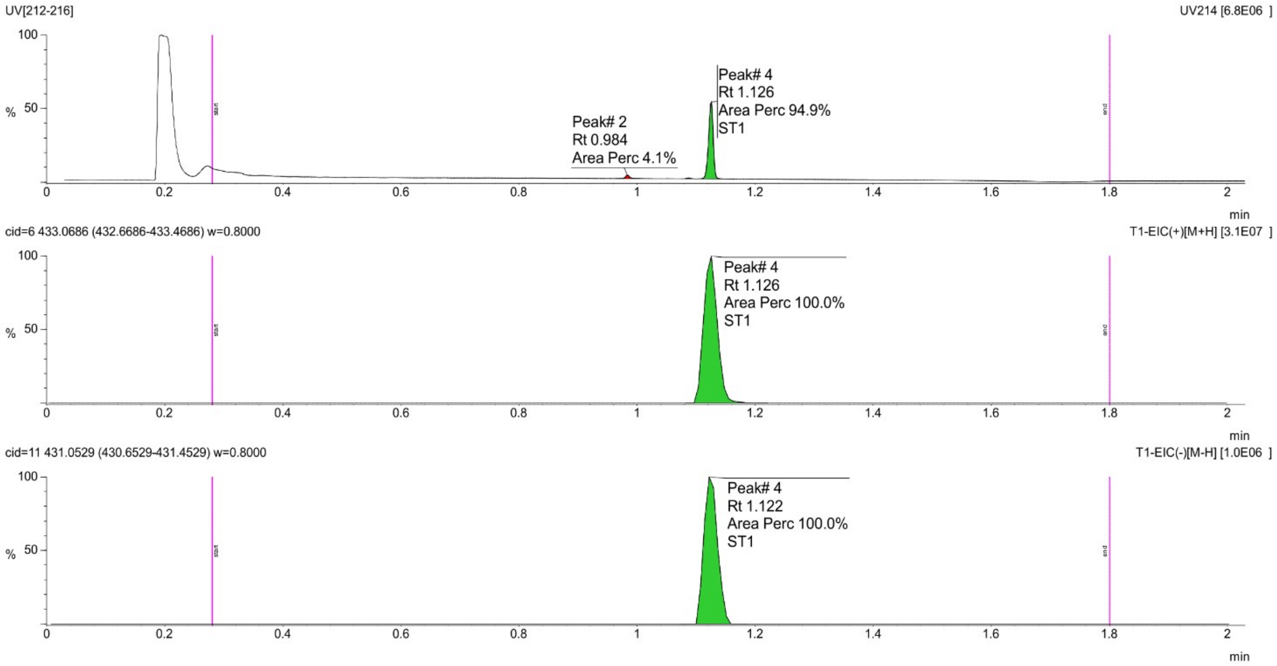

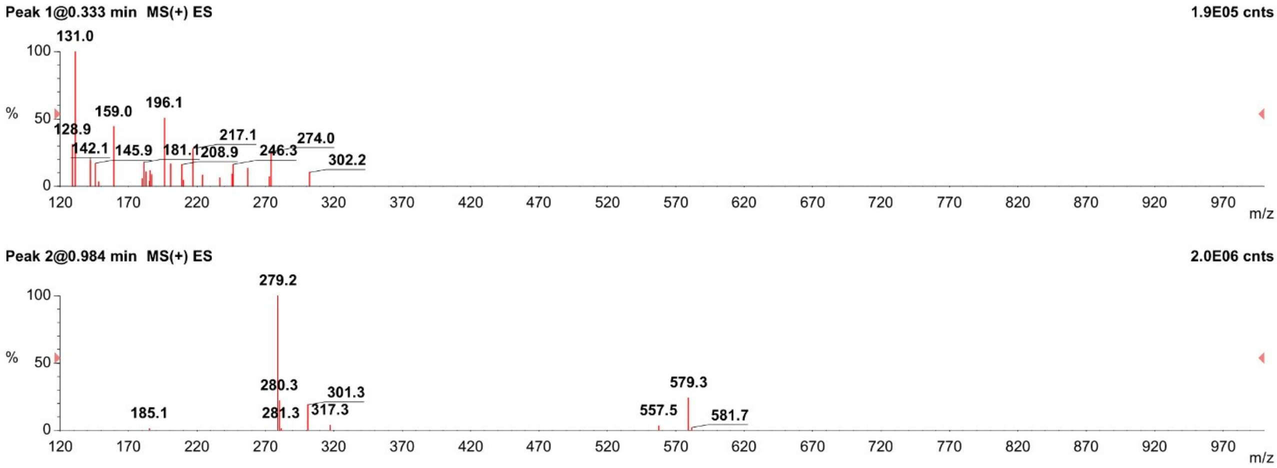

Printed: 10/6/2022 1:49 PM

This journal is © The Royal Society of Chemistry 20xx

Page 2 of 6

HTS QC Analysis Report

Sample Name: LCMS384-20221005-1_G11

Compound 16

Instrument: ICHALW-DL00012-TQD

Acquisition time: 10/6/2022 12:14 AM

Peak 3@1.087 min MS(+) ES **74956.8 cnts**

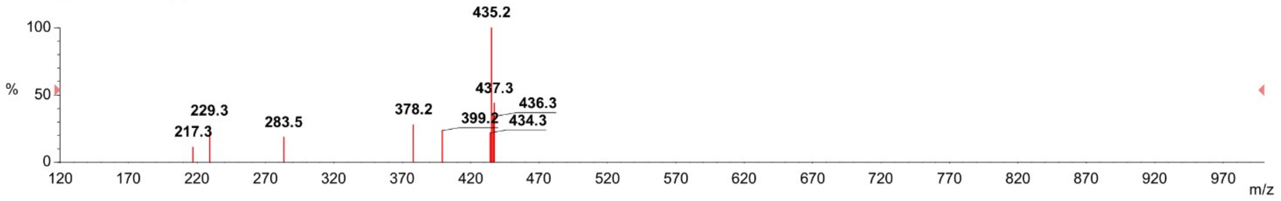

Peak 4@1.126 min MS(+) ES **2.2E07 cnts**

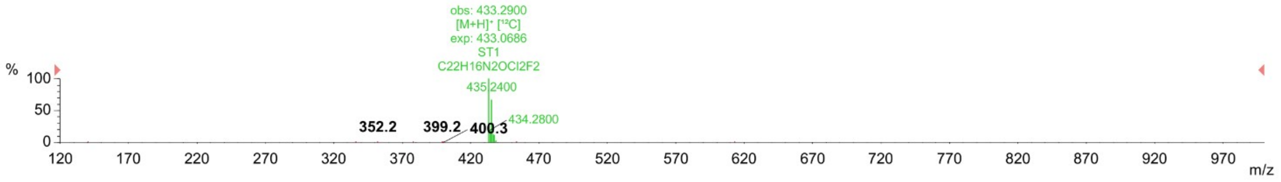

Printed: 10/6/2022 1:49 PM

HTS QC Analysis Report

Sample Name: LCMS384-20221005-1_A13

Compound 17

Instrument: ICHALW-DL00012-TQD

Acquisition time: 10/6/2022 12:29 AM

Expression	Result
Auto-Comments	
Auto-Summary	GOOD
Purity	99.1000
Target Mass	357.2000
Target RT	0.8540

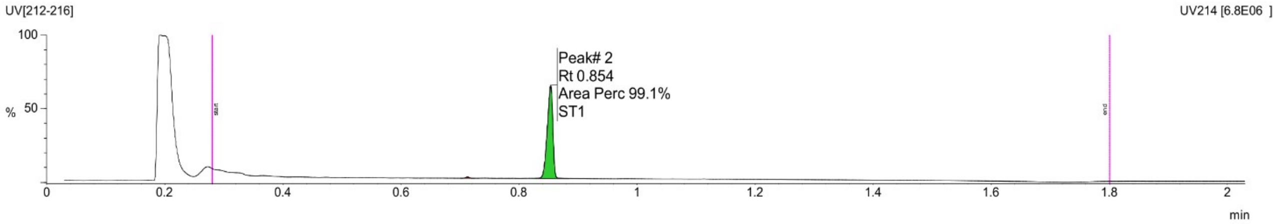

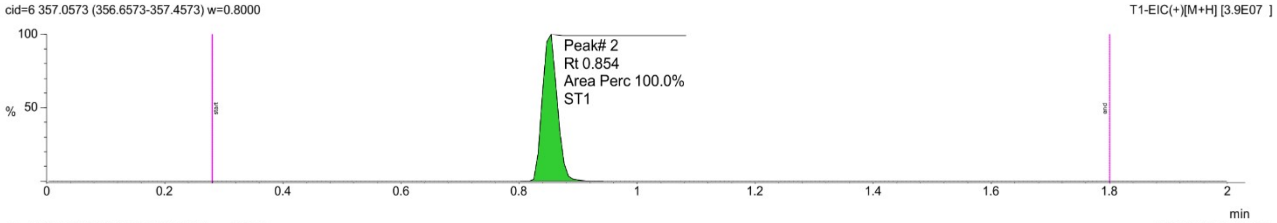

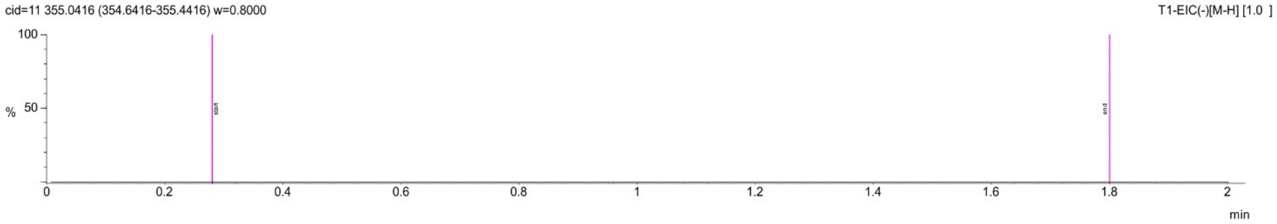

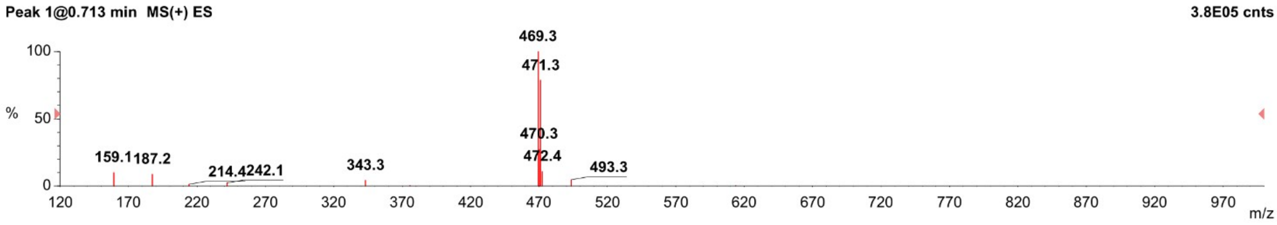

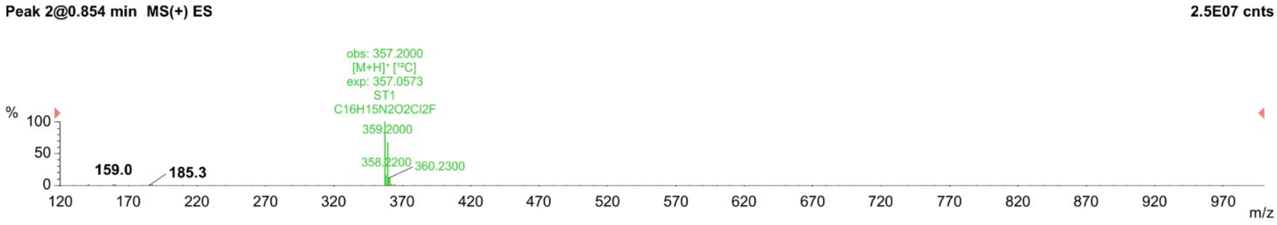

Printed: 10/6/2022 1:49 PM

This journal is © The Royal Society of Chemistry 20xx

HTS QC Analysis Report

Sample Name: LCMS384-20221005-1_G13

Compound 18

Instrument: ICHALW-DL00012-TQD

Acquisition time: 10/6/2022 12:38 AM

Expression	Result
Auto-Comments	
Auto-Summary	GOOD
Purity	97.9000
Target Mass	354.2000
Target RT	0.5260

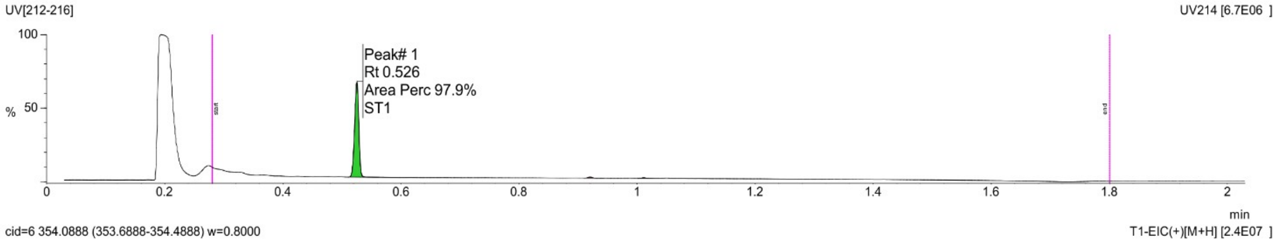

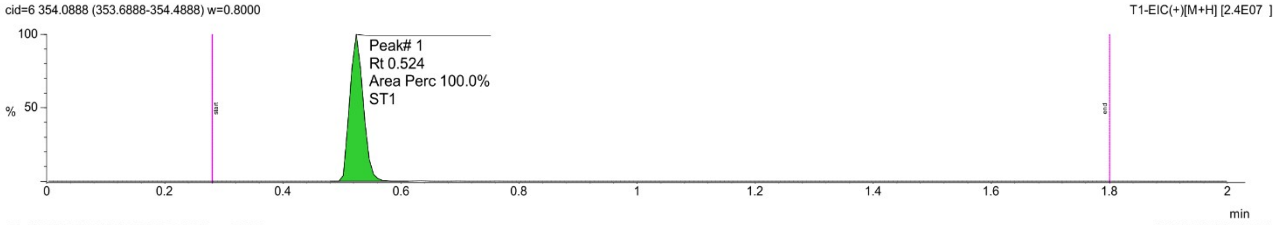

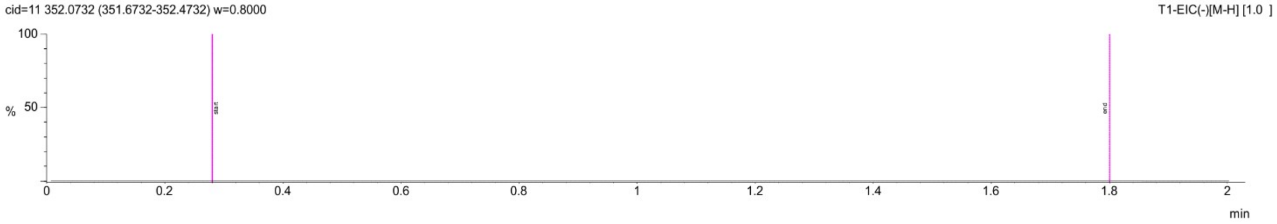

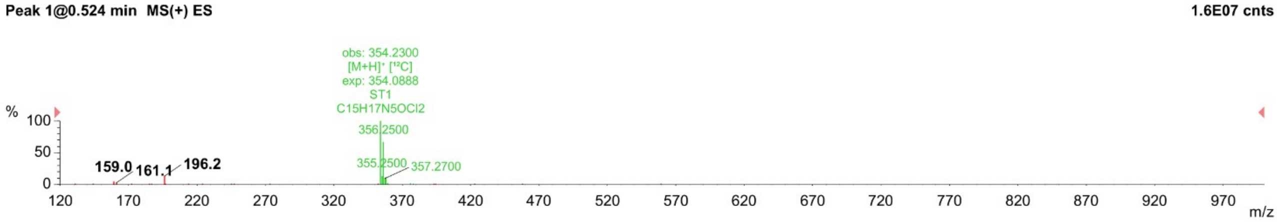

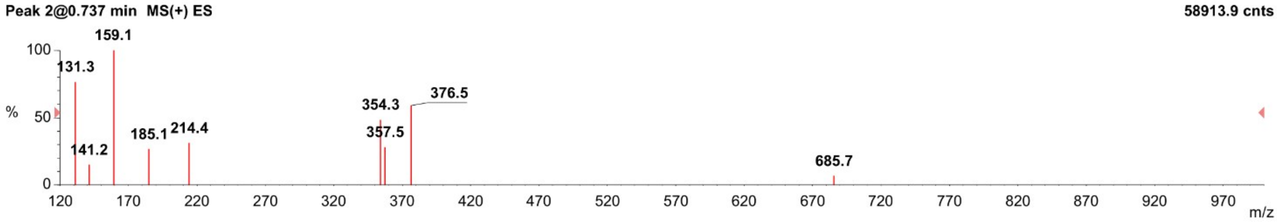

Printed: 10/6/2022 1:48 PM

Please do not adjust margins

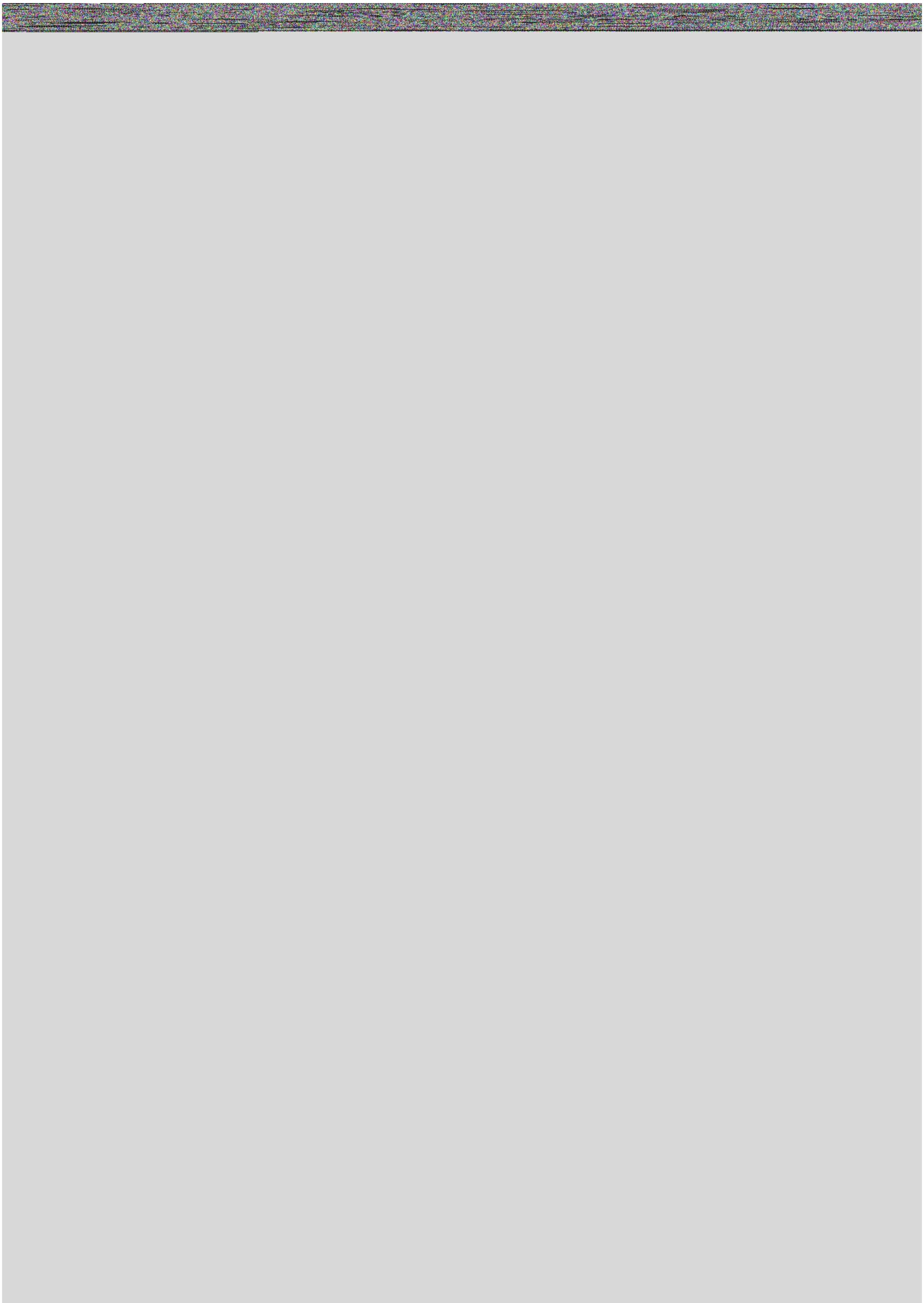

This journal is © The Royal Society of Chemistry 20xx

Please do not adjust margins

Please do not adjust margins

idorsia HTS QC Analysis Report

Sample Name: LCMS384-20221005-1_I13

Compound 19

Instrument: ICHALW-DL00012-TQD

Acquisition time: 10/6/2022 12:41 AM

Expression	Result
Auto-Comments	
Auto-Summary	GOOD
Purity	97.4000
Target Mass	286.2000
Target RT	0.8540

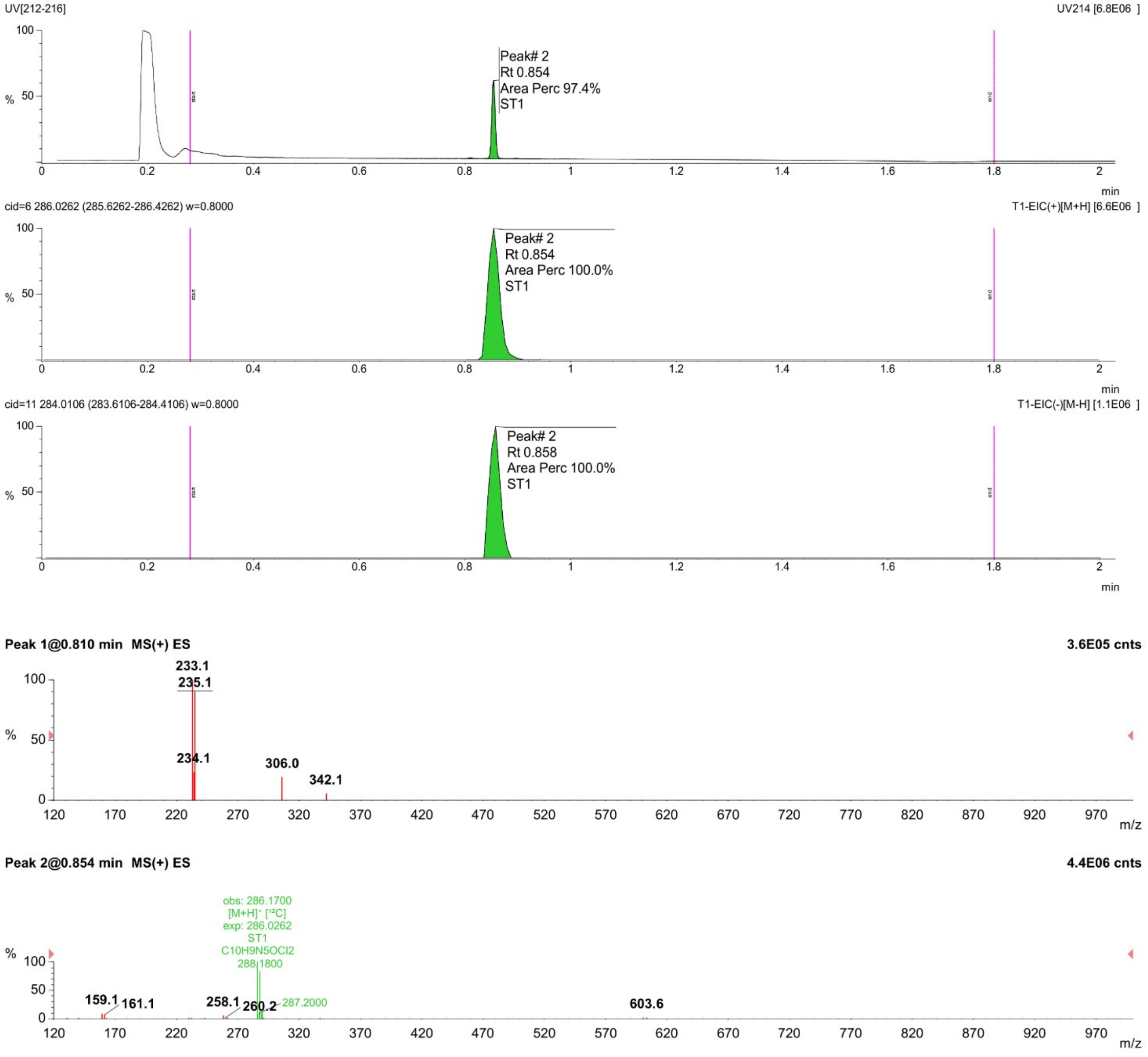

Printed: 10/6/2022 1:48 PM

Please do not adjust margins

idorsia HTS QC Analysis Report

Sample Name: LCMS384-20221005-1_K11

Compound 20
Instrument: ICHALW-DL00012-TQD

Acquisition time: 10/6/2022 12:20 AM

Expression	Result
Auto-Comments	
Auto-Summary	GOOD
Purity	98.2000
Target Mass	325.2000
Target RT	0.9660

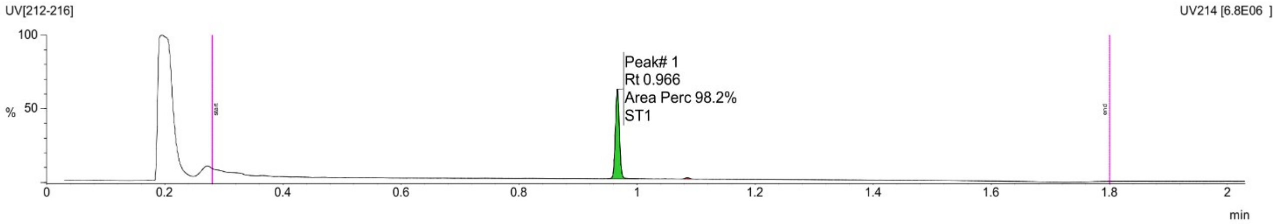

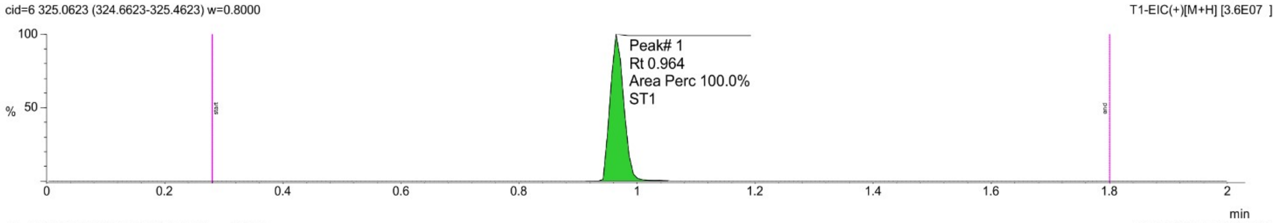

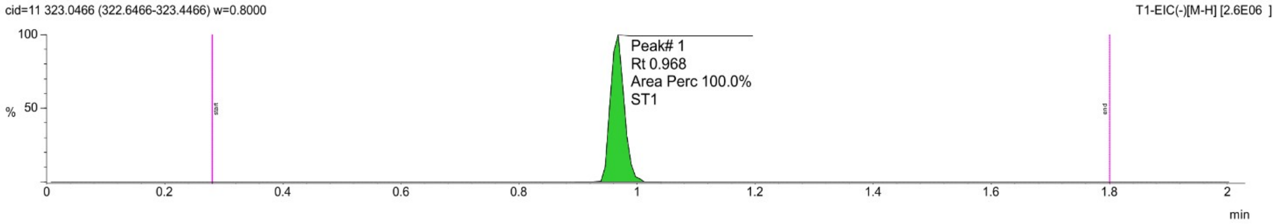

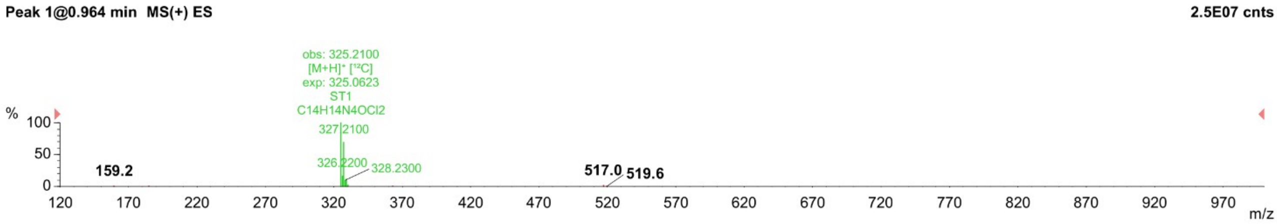

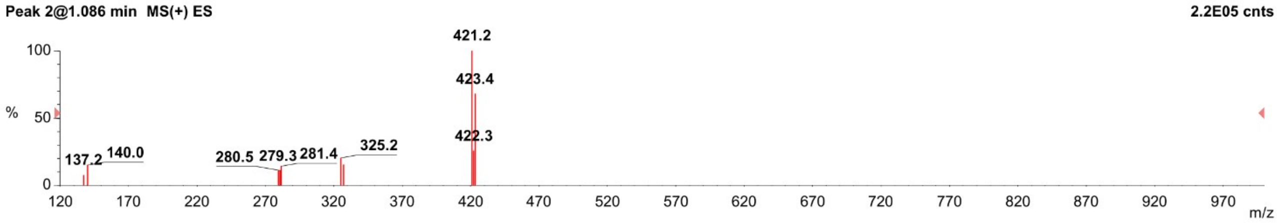

Printed: 10/6/2022 1:49 PM

This journal is © The Royal Society of Chemistry 20xx

Please do not adjust margins

HTS QC Analysis Report

Sample Name: LCMS384-20221020-3_I07

Compound 21

Instrument: ICHALW-DL00021-SQD

Acquisition time: 10/20/2022 4:14 PM

Expression	Result
Auto-Comments	
Auto-Summary	GOOD
Purity	98.9000
Target Mass	354.4000
Target RT	0.6700

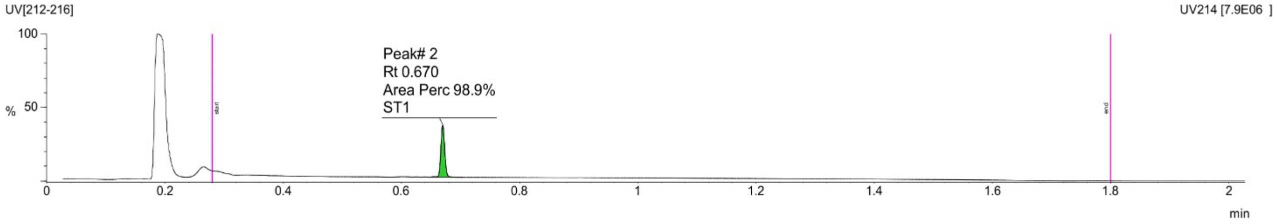

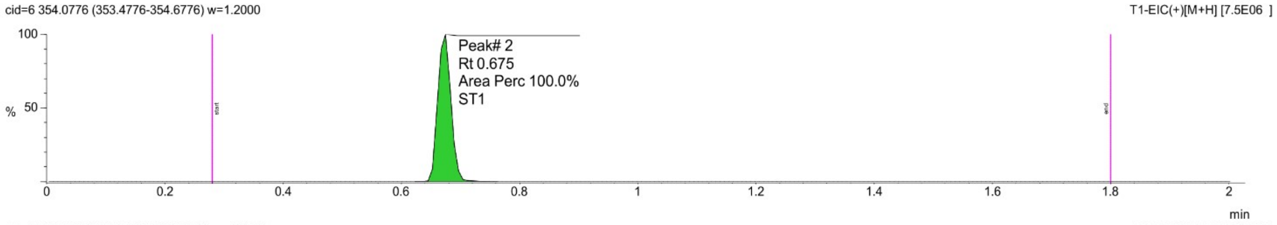

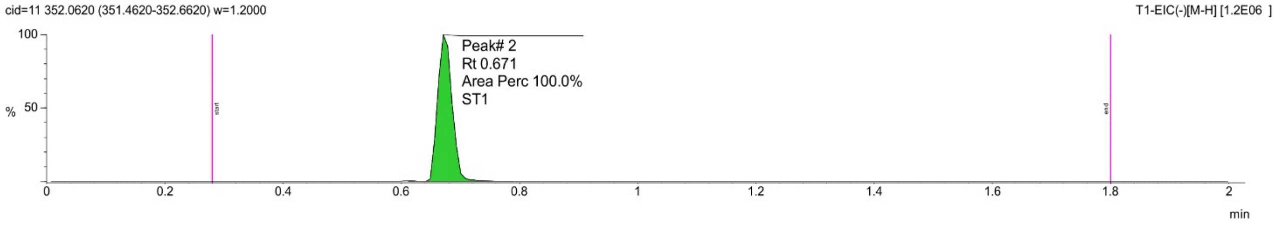

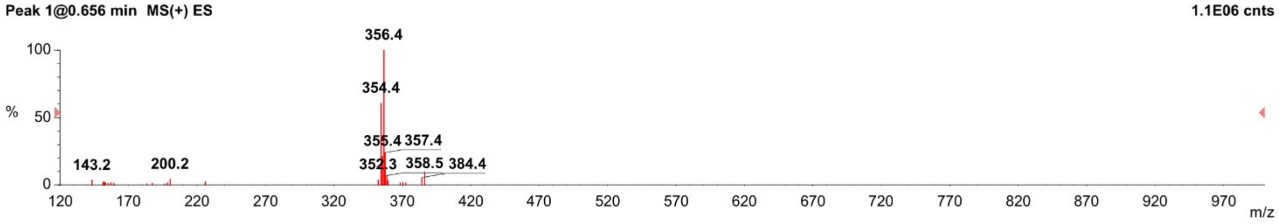

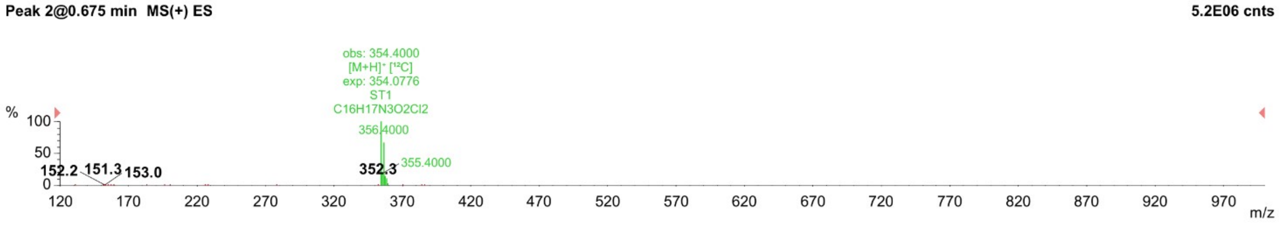

Printed: 10/21/2022 9:40 AM

idorsia **HTS QC Analysis Report**

Sample Name: LCMS384-20221109-4_K01

Compound 22

Instrument: ICHALW-DL00012-TQD

Acquisition time: 11/9/2022 3:51 PM

Expression	Result
Auto-Comments	
Auto-Summary	GOOD
Purity	91.3000
Target Mass	299.2000
Target RT	0.6560

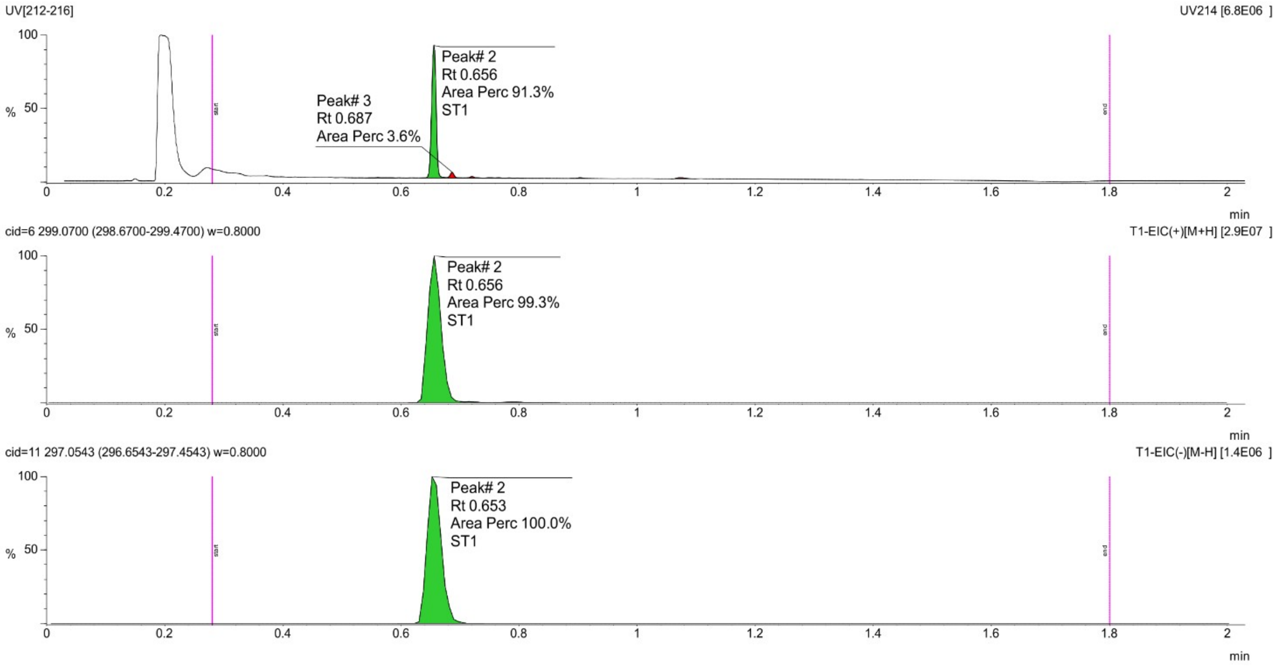

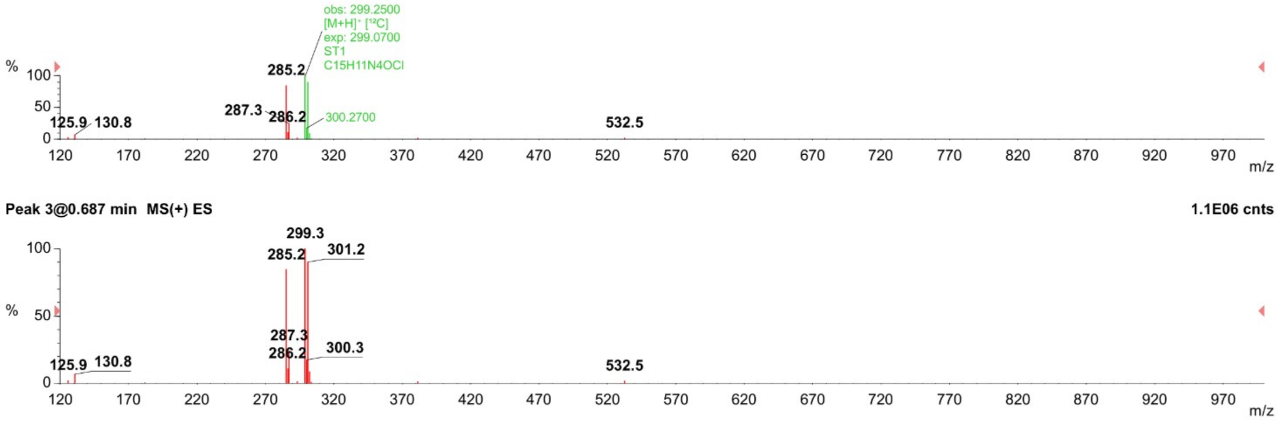

Printed: 11/10/2022 8:45 AM

This journal is © The Royal Society of Chemistry 20xx

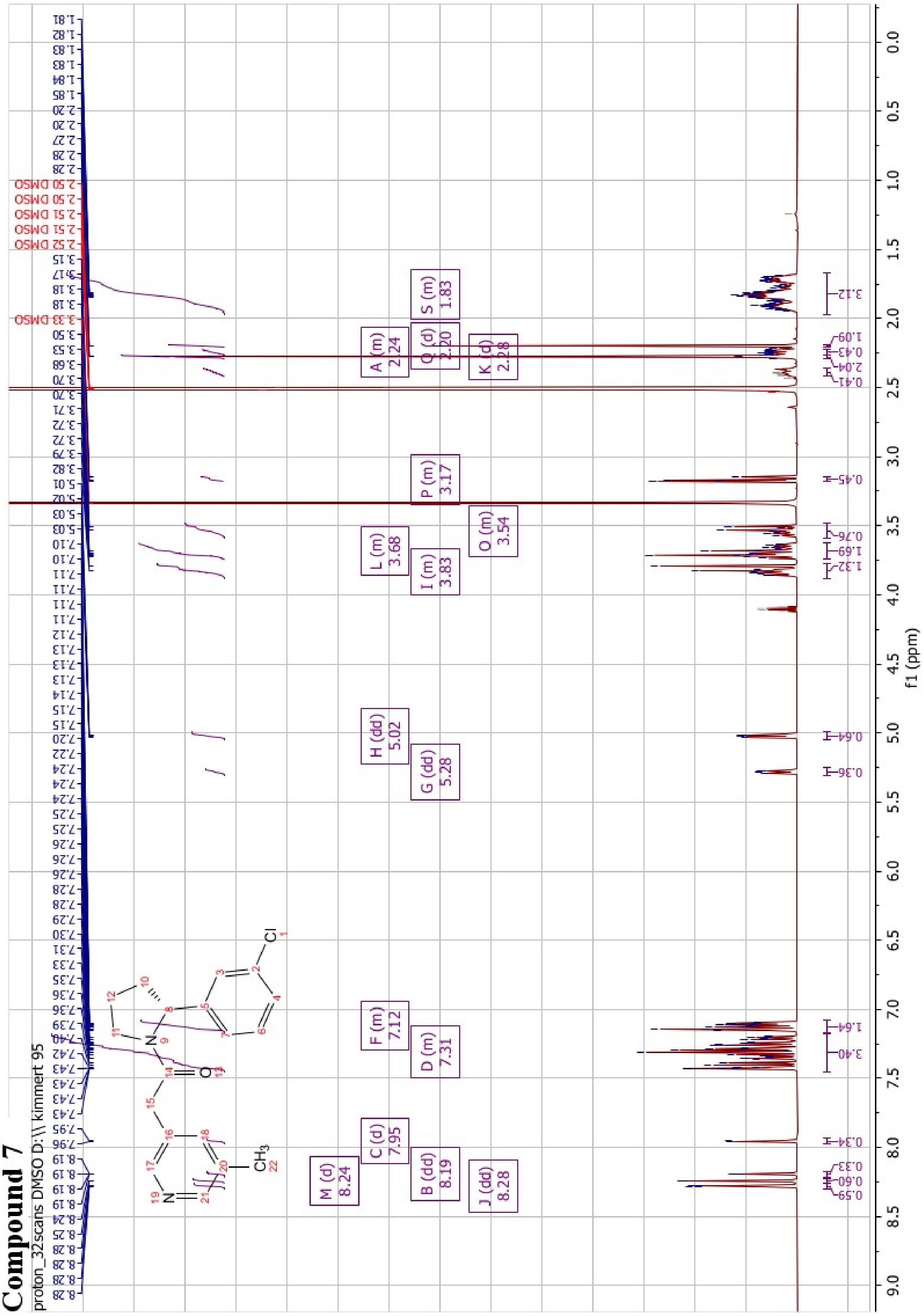
Compound 7
proton_32scans DMSO D:\\ kimmert 95
f1 (ppm)

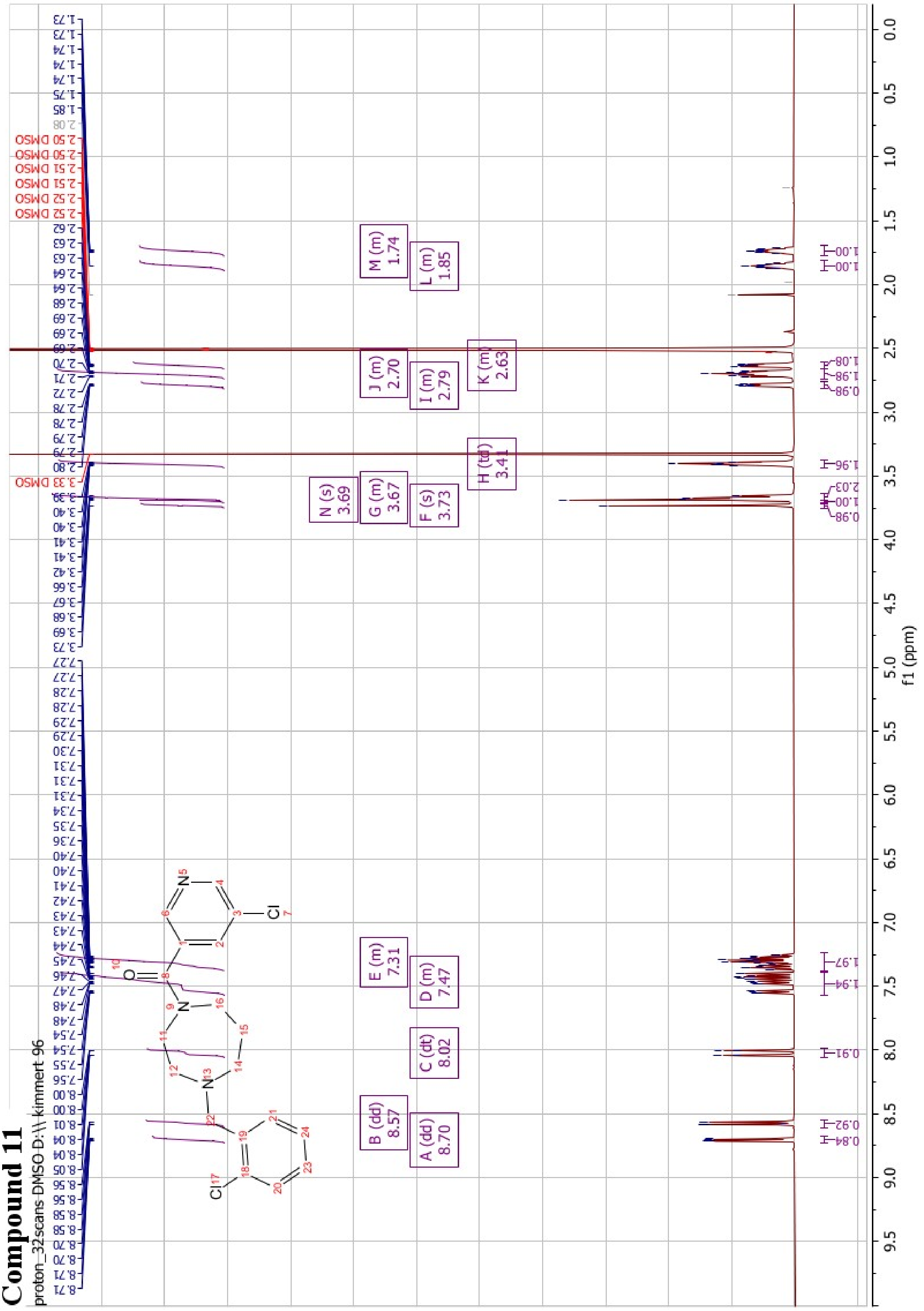

This journal is © The Royal Society of Chemistry 20xx

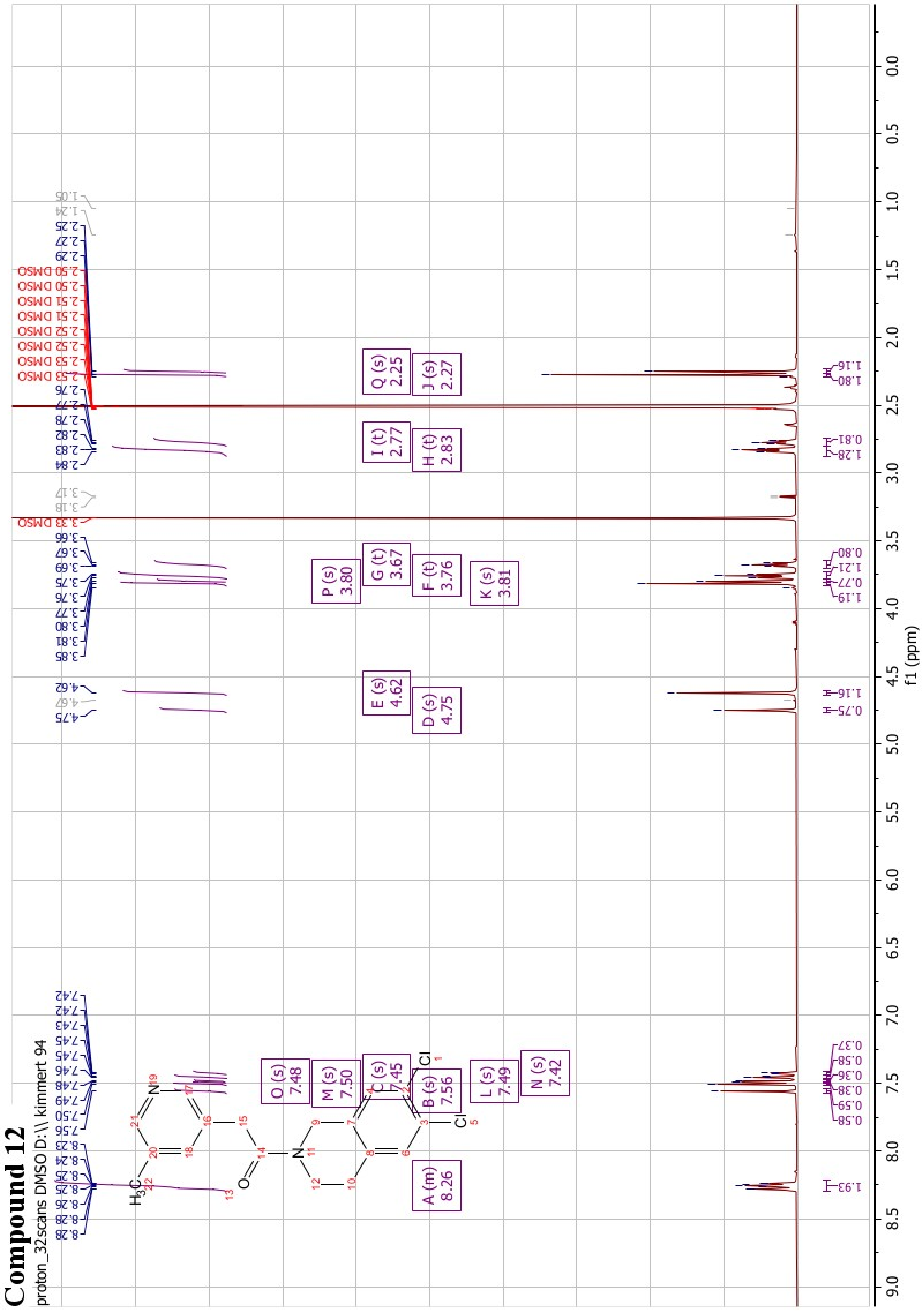
Compound 12
proton_32scans DMSO D:\\ kimmert 94
f1 (ppm)

Protein sequence pCG 163: 442 aa excl. stop codon
→ Q306E mutation destroys occurring N-terminal selfcleavage site to maintain AVI-tag

H6.SUMO.Covid-19 Mpro C145A Q306E.AVI
(Thrombin cleavage site, SUMO cleavage site, residue Mpro autocleavage site)

```
        10         20         30         40         50         60
MGSSHHHHHH GSGLVPRGSA SMSDSEVDQE AKPEVKPEVK PETHINLKVS DGSSEIFFKI
        70         80         90        100        110        120
KKTTPLRRLM EAFAKRQGKE MDSLRFLYDG IRIQADQTPE DLDMEDNDII EAHREQIGG↓S
       130        140        150        160        170        180
GFRKMAFPSG KVEGCMVQVT CGTTTLNGLW LDDVVYCPRH VICTSEDMLN PNYEDLLIRK
       190        200        210        220        230        240
SNHNFLVQAG NVQLRVIGHS MQNCVLKLKV DTANPKTPKY KFVRIQPGQT FSVLACYNGS
       250        260        270        280        290        300
PSGVYQCAMR PNFTIKGSFL NGSAGSVGFN IDYDCVSFCY MHHMELPTGV HAGTDLEGNF
       310        320        330        340        350        360
YGPFVDRQTA QAAGTDTTIT VNVLAWLYAA VINGDRWFLN RFTTTLNDFN LVAMKYNYEP
       370        380        390        400        410        420
LTQDHVDILG PLSAQTGIAV LDMCASLKEL LQNGMNGRTI LGSALLEDEF TPFDVVRQCS
       430        440  442
GVTFEGSGLN DIFEAQKIEW HE *
```


This journal is © The Royal Society of Chemistry 20xx

Please do not adjust margins
Please do not adjust margins
